# Supplementary material for: Simple discrete-time self-exciting models can describe complex dynamic processes: A case study of COVID-19
Source: PLoS One. 2021 Apr 9;16(4):e0250015. doi: 10.1371/journal.pone.0250015 (PMC8034752; doi:10.1371/journal.pone.0250015)
Supplement: S1 Appendix — (PDF) [file pone.0250015.s001.pdf]

S1 Appendix: Justification for Hawkes model on deaths

In this section we provide some interpretation of our model on the number of deaths, relating it to the underlying number of infections. This allows us to interpret the parameters in terms of driving parameters for the contamination process.

Hawkes processes are natural objects to model causality through time. Hence, they are well adapted to model the infections, since infections are causing other infections. Assume that at time t (say on the daily basis) N_t^I are newly infected and assume that given the past N_t^I follows a distribution Poisson with rate λ_t^I such that,

$$N_t^I \sim \mathcal{P}(\lambda_t^I), \quad \lambda_t^I = \mu_I + \alpha\beta \sum_{k>0} N_{t-k}^I (1-\beta)^{k-1})$$

where μ_I is the background rate of arrivals with respect to infections.

Consider the simplifying assumption that it takes the same amount of time Δ for a person to die, i.e. people infected at time t will die - given that they will die - at time $t+\Delta$. Hence given that N_t^I people were infected at time t the number of deaths at time $t+\Delta$, $N_{t+\Delta}^D$, is a Binomial (p, N_t^I). When p is small and N_t^I is large this is approximately a Poisson with parameter pN_t^I. This assumption is reasonable since the global death rate is small. Moreover if λ_t^I is large, $N_t^I = \lambda_t^I \Big(1+O(1/\sqrt{\lambda_t^I})\Big)$. Hence, conditionnally on the past, $N_{t+\Delta}^D$ approximately follows a Poisson distribution with parameter

$$\lambda_{t+\Delta}^D = p\lambda_t^I (1 + 1/\sqrt{\lambda_t^I}) \approx [p\mu_I + \alpha\beta \sum_{k>0} pN_{t-k}^I (1-\beta)^{k-1}](1+o(1)).$$

In a regime where pN_{t-k}^I is large, $N_{t+\Delta-k}^D \approx pN_{t-k}^I$ and to the first order

$$\lambda_{t+\Delta}^D = p\lambda_t^I (1 + 1/\sqrt{\lambda_t^I}) \approx [p\mu_I + \alpha\beta \sum_{k>0} N_{t+\Delta-k}^D (1+o(1))(1-\beta)^{k-1}](1+o(1)).$$

Hence the parameters α and β in the death model have the same meaning as in the infection model. In particular α can be interpreted to the first order as the average number of people contaminated by an infected person and the external arrival rate μ in the death model is roughly the external infection rate μ_I multiplied by the probability of dying given that one is infected.
