# Supplementary material for: Simple discrete-time self-exciting models can describe complex dynamic processes: A case study of COVID-19
Source: PLoS One. 2021 Apr 9;16(4):e0250015. doi: 10.1371/journal.pone.0250015 (PMC8034752; doi:10.1371/journal.pone.0250015)
Supplement: S2 Appendix — (PDF) [file pone.0250015.s002.pdf]

S2 Appendix: About the average excitation duration

Under the link established in S1 Appendix with the infection model, then what we have is that an infected individual will in the future infect individuals at different times following this model: let us consider case 0, an infected individual,

$$\sum_{t=1}^{\infty} \zeta_t, \quad \zeta_t \sim \mathcal{P}(\alpha\beta(1-\beta)^{t-1})$$

where ζ_t corresponds to the number of individuals infected by case 0. The average number of individuals infected by case 0 is α. One can define the average time of infections from patient 0 as $\bar{T} = \sum_{t=1}^{\infty} t\zeta_t/\alpha$ and its expectation is given by

$$E(\bar{T}) = \sum_{t=1}^{\infty} t\beta(1-\beta)^{t-1} = \frac{1}{\beta}.$$

Hence $1/\beta$ can be interpreted as the expectation of the average time of infections created by patient 0 over the average number of infections. On the other hand, the maximal duration of effective infectiousness is given by $T_0 = \max\{q; \sum_{t\geq q} \zeta_t > 0\}$ so that

$$P(T_0 < q) = P\Big(\sum_{t\geq q} \zeta_t = 0\Big) = e^{-\alpha(1-\beta)^{q-1}}, \quad q \geq 1$$

and

$$E(T_0) = \sum_{q\geq 1} P(T_0 \geq q) = \sum_{q\geq 0} [1 - e^{-\alpha(1-\beta)^q}].$$

Using $u - \frac{u^2}{2} \leq 1 - e^{-u} \leq u, \quad u \geq 0$, we can bound

$$1 - e^{-\alpha} + \frac{\alpha(1-\beta)}{\beta} - \frac{\alpha^2(1-\beta)^2}{4\beta(1-\beta/2)} \leq E(T_0) \leq 1 - e^{-\alpha} + \frac{\alpha(1-\beta)}{\beta}.$$

So that if β is close to 1 (which is the case in the first phase of our analysis),

$$E(T_0) \approx 1 - e^{-\alpha}$$

while if β is close to 0 (and α is not too large),

$$E(T_0) \approx \frac{c(\alpha)}{\beta}, \quad \alpha(1-\alpha/4) \leq c(\alpha) \leq \alpha.$$
