# Supplementary material for: Simple discrete-time self-exciting models can describe complex dynamic processes: A case study of COVID-19
Source: PLoS One. 2021 Apr 9;16(4):e0250015. doi: 10.1371/journal.pone.0250015 (PMC8034752; doi:10.1371/journal.pone.0250015)
Supplement: S3 Appendix — Top left hand panel: compares the observed number of deaths (black dots) with the 95% posterior interval for the estimated expected number of events (solid red ribbon). Top right hand panel: shows pairwise correlation between all parameters in the lower triangle, corresponding correlation values in the upper triangle, and the marginal posterior densities for each parameter on the diagonal. Bottom panel: shows trace plots on the top row and the autocorrelation function on the bottom row for each parameter. All figures were generated after thinning the posterior samples. (PDF) [file pone.0250015.s003.pdf]

S3 Appendix: Convergence and diagnostic plots for initial and subsequent analysis

Initial analysis

Here we present diagnostic checks for the model where $\mu_1, \mu_2 \sim \text{Gamma}(5, 1)$. Similar results were obtained for all prior choices considered for μ_1, μ_2.

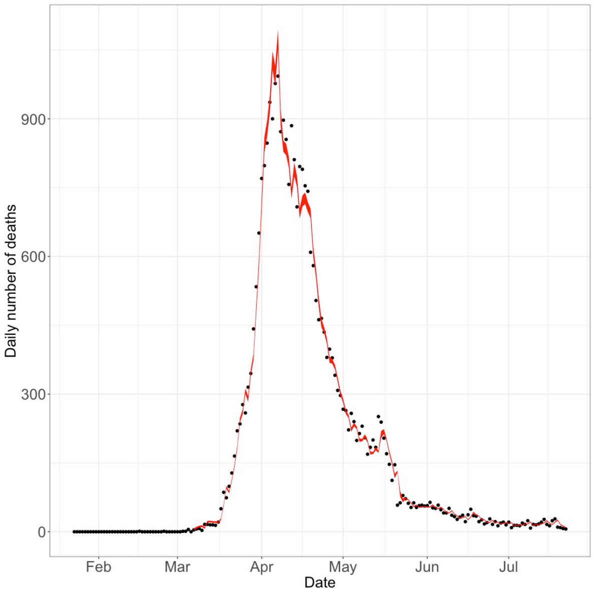

(a)

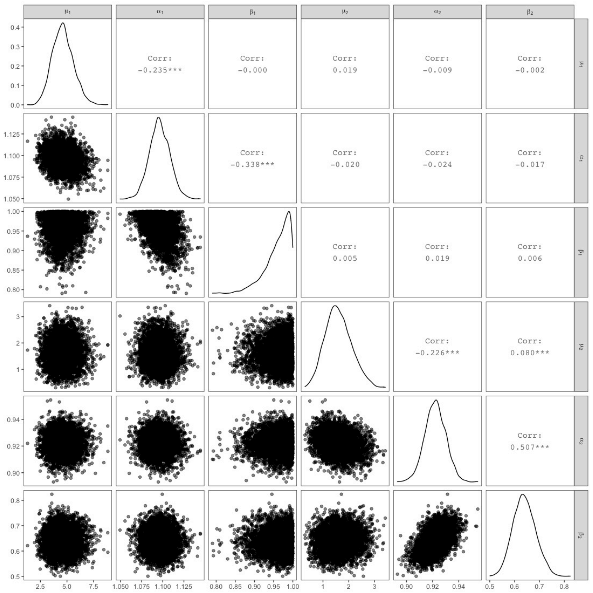

(b)

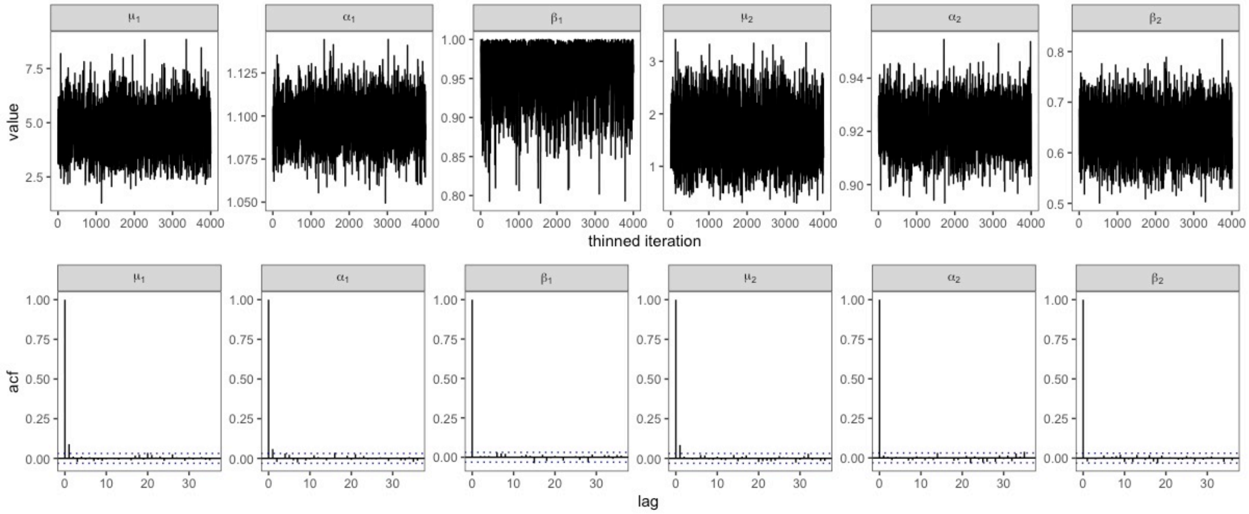

(c)

Fig 1. Convergence and diagnostic plots for France. Top left hand panel: compares the observed number of deaths (black dots) with the 95% posterior interval for the estimated expected number of events (solid red ribbon). Top right hand panel: shows pairwise correlation between all parameters in the lower triangle, corresponding correlation values in the upper triangle, and the marginal posterior densities for each parameter on the diagonal. Bottom panel: shows trace plots on the top row and the autocorrelation function on the bottom row for each parameter. All figures were generated after thinning the posterior samples.

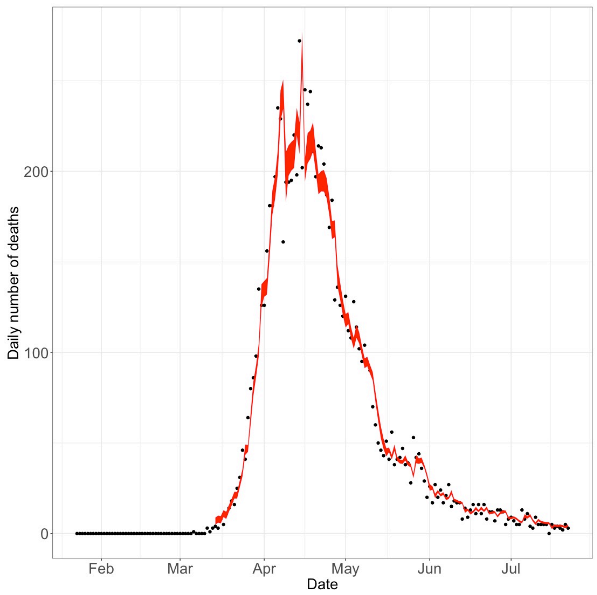

(a)

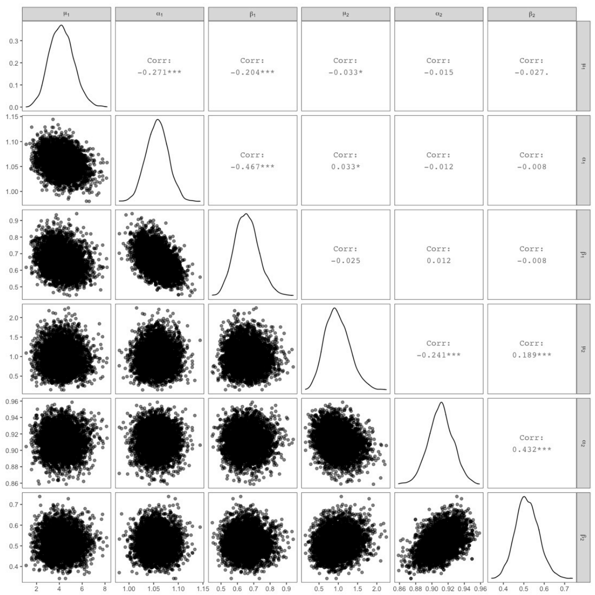

(b)

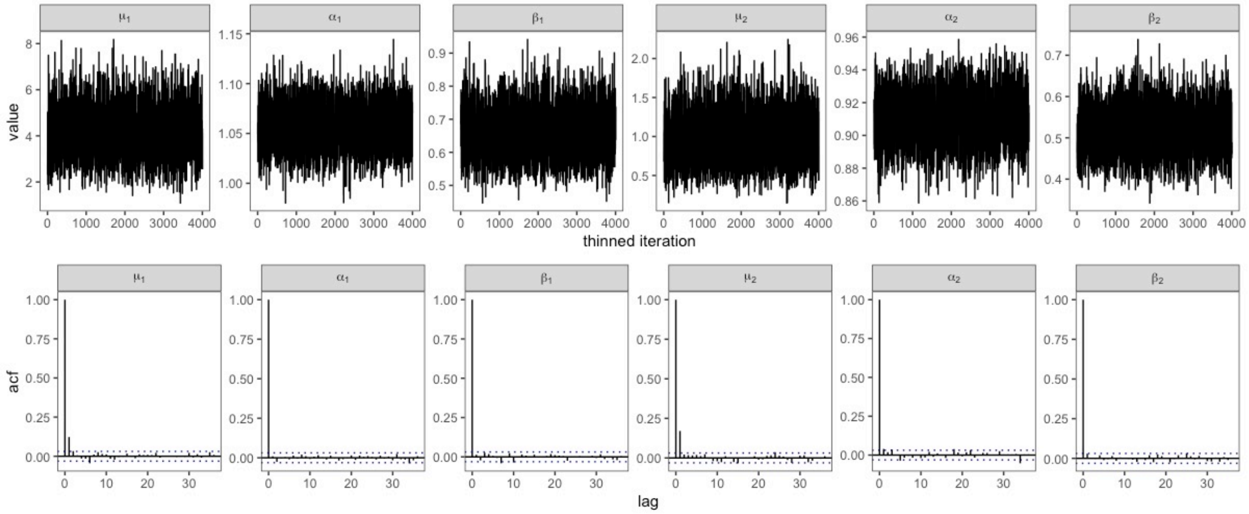

(c)

Fig 2. Convergence and diagnostic plots for Germany. Top left hand panel: compares the observed number of deaths (black dots) with the 95% posterior interval for the estimated expected number of events (solid red ribbon). Top right hand panel: shows pairwise correlation between all parameters in the lower triangle, corresponding correlation values in the upper triangle, and the marginal posterior densities for each parameter on the diagonal. Bottom panel: shows trace plots on the top row and the autocorrelation function on the bottom row for each parameter. All figures were generated after thinning the posterior samples.

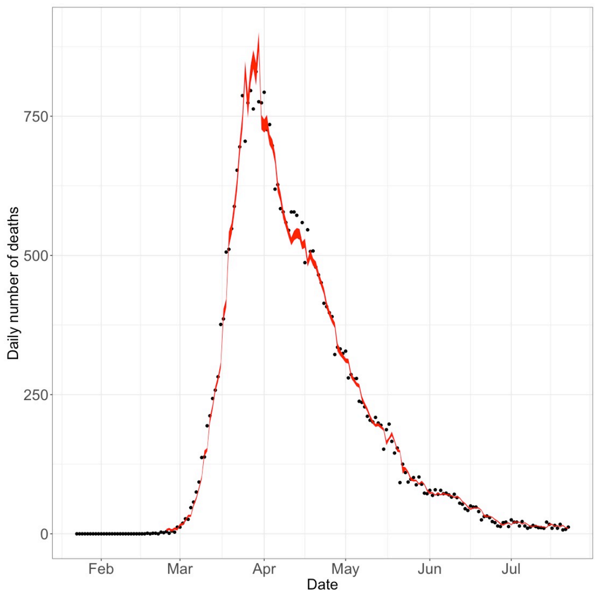

(a)

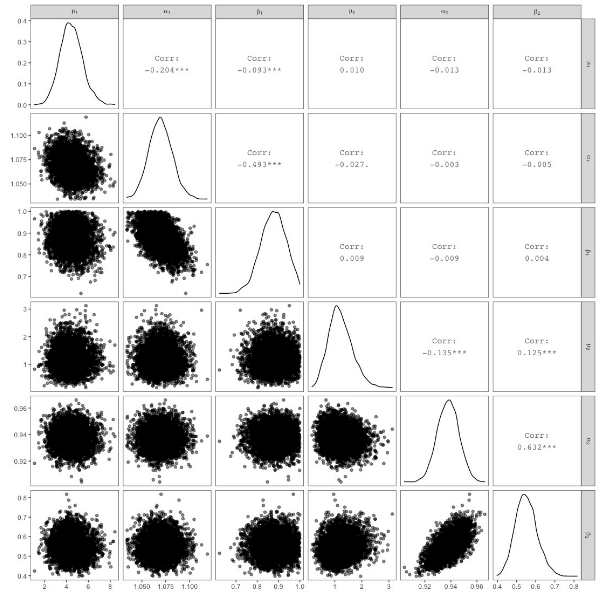

(b)

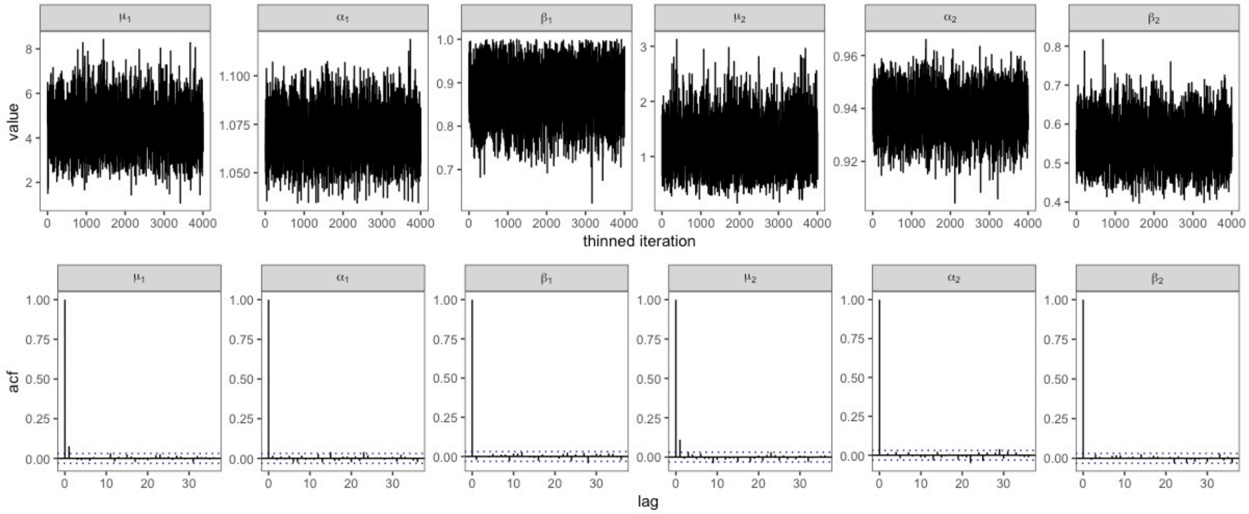

(c)

Fig 3. Convergence and diagnostic plots for Italy. Top left hand panel: compares the observed number of deaths (black dots) with the 95% posterior interval for the estimated expected number of events (solid red ribbon). Top right hand panel: shows pairwise correlation between all parameters in the lower triangle, corresponding correlation values in the upper triangle, and the marginal posterior densities for each parameter on the diagonal. Bottom panel: shows trace plots on the top row and the autocorrelation function on the bottom row for each parameter. All figures were generated after thinning the posterior samples.

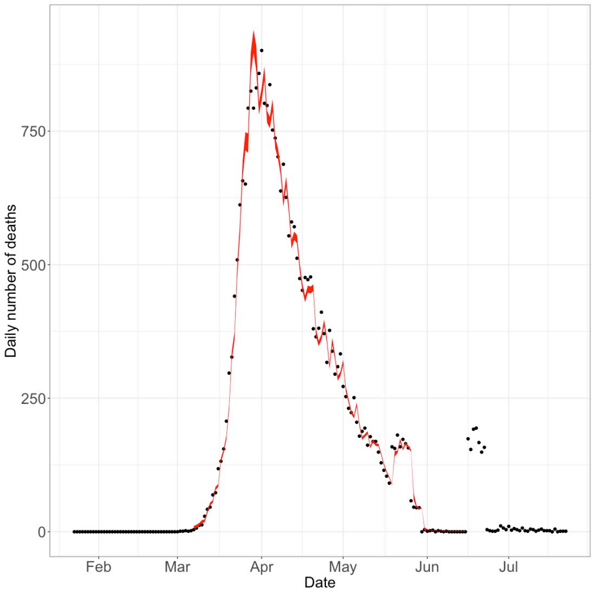

(a)

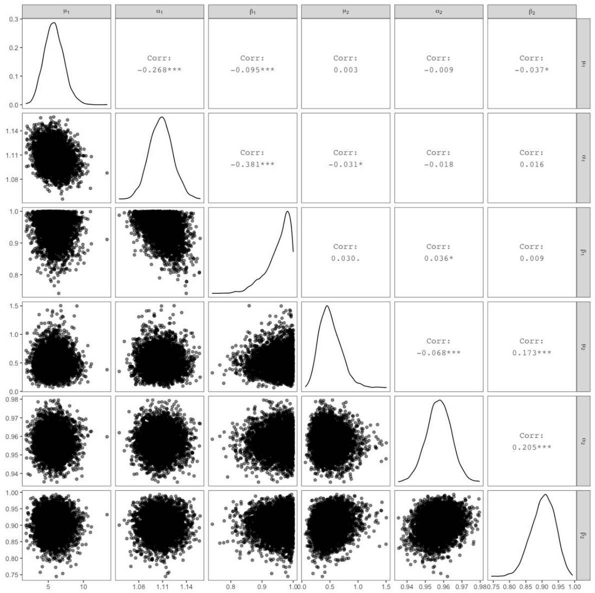

(b)

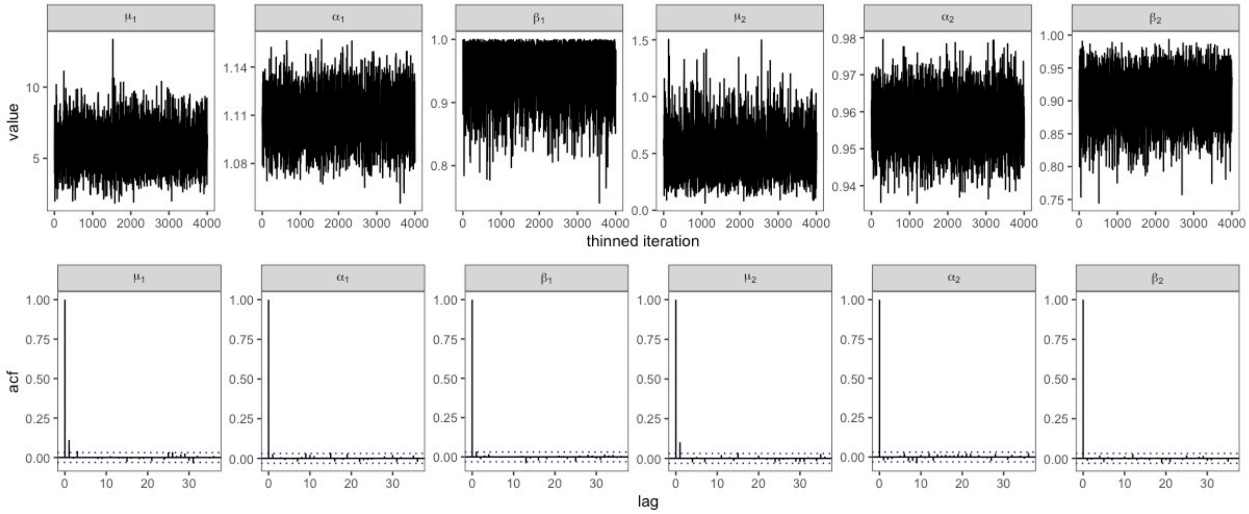

(c)

Fig 4. Convergence and diagnostic plots for Spain. Top left hand panel: compares the observed number of deaths (black dots) with the 95% posterior interval for the estimated expected number of events (solid red ribbon). Top right hand panel: shows pairwise correlation between all parameters in the lower triangle, corresponding correlation values in the upper triangle, and the marginal posterior densities for each parameter on the diagonal. Bottom panel: shows trace plots on the top row and the autocorrelation function on the bottom row for each parameter. All figures were generated after thinning the posterior samples.

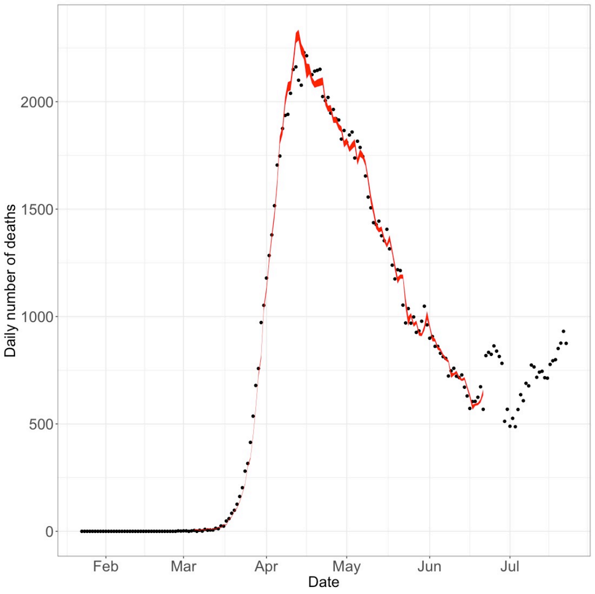

(a)

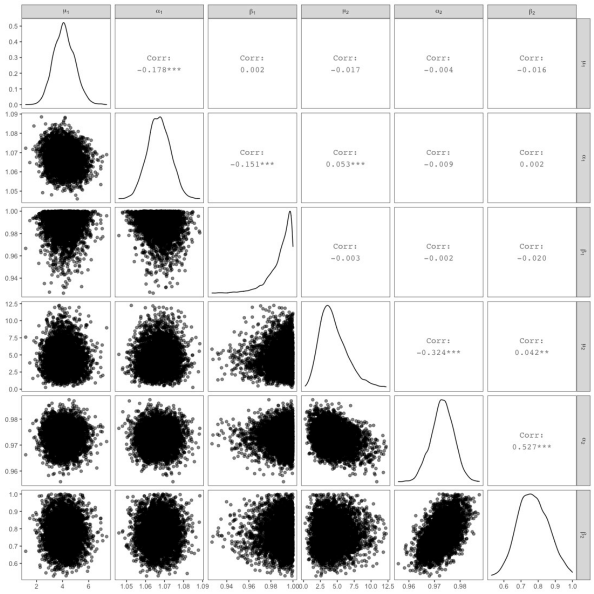

(b)

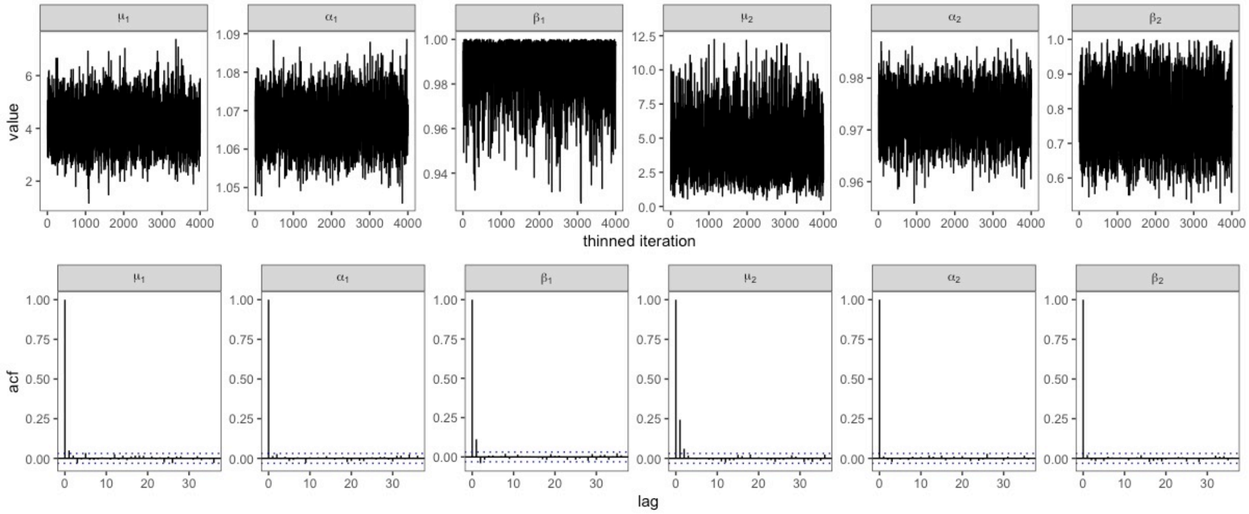

(c)

Fig 5. Convergence and diagnostic plots for the U.S. Top left hand panel: compares the observed number of deaths (black dots) with the 95% posterior interval for the estimated expected number of events (solid red ribbon). Top right hand panel: shows pairwise correlation between all parameters in the lower triangle, corresponding correlation values in the upper triangle, and the marginal posterior densities for each parameter on the diagonal. Bottom panel: shows trace plots on the top row and the autocorrelation function on the bottom row for each parameter. All figures were generated after thinning the posterior samples.

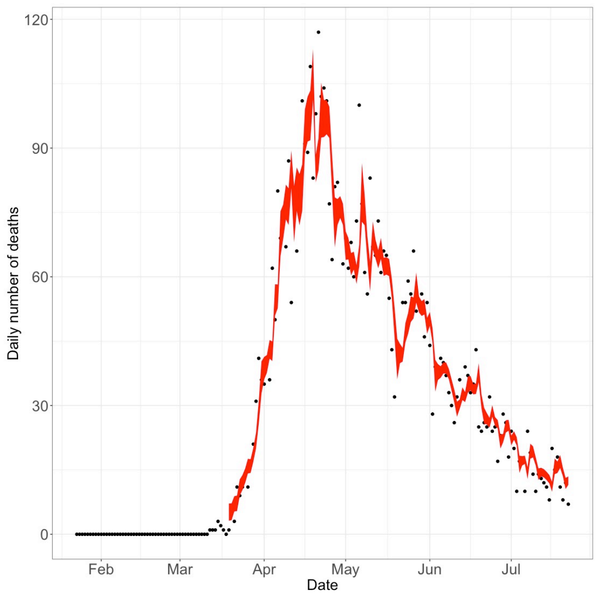

(a)

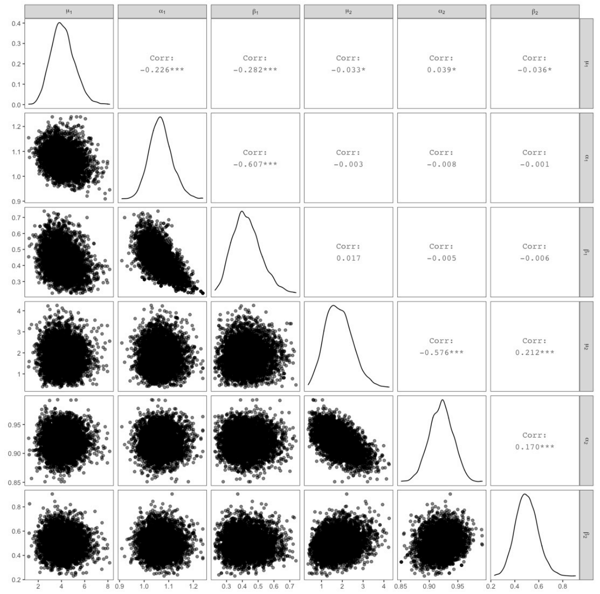

(b)

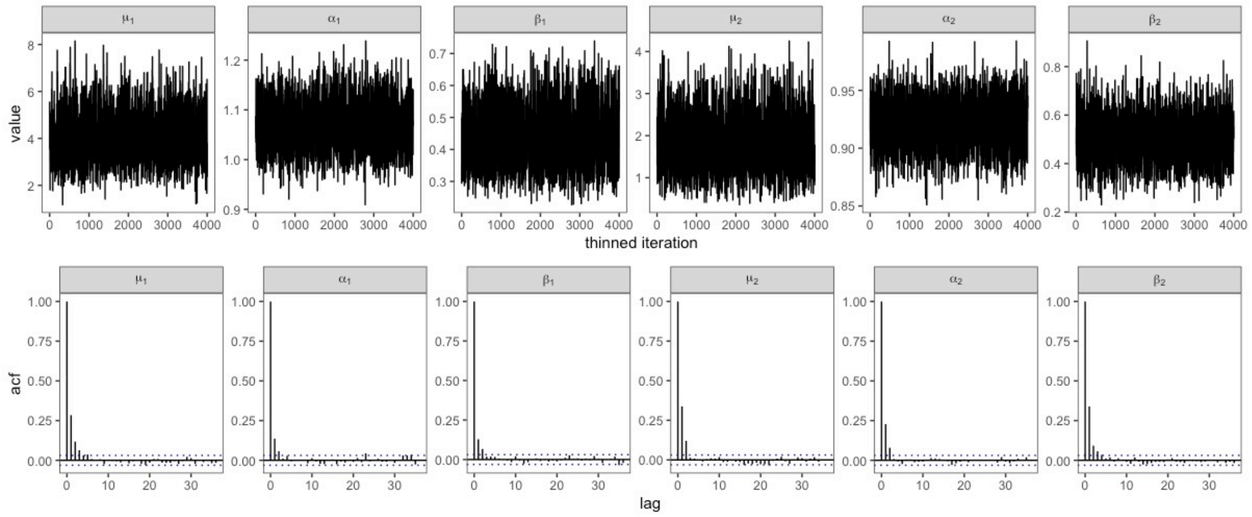

(c)

Fig 6. Convergence and diagnostic plots for Sweden. Top left hand panel: compares the observed number of deaths (black dots) with the 95% posterior interval for the estimated expected number of events (solid red ribbon). Top right hand panel: shows pairwise correlation between all parameters in the lower triangle, corresponding correlation values in the upper triangle, and the marginal posterior densities for each parameter on the diagonal. Bottom panel: shows trace plots on the top row and the autocorrelation function on the bottom row for each parameter. All figures were generated after thinning the posterior samples.

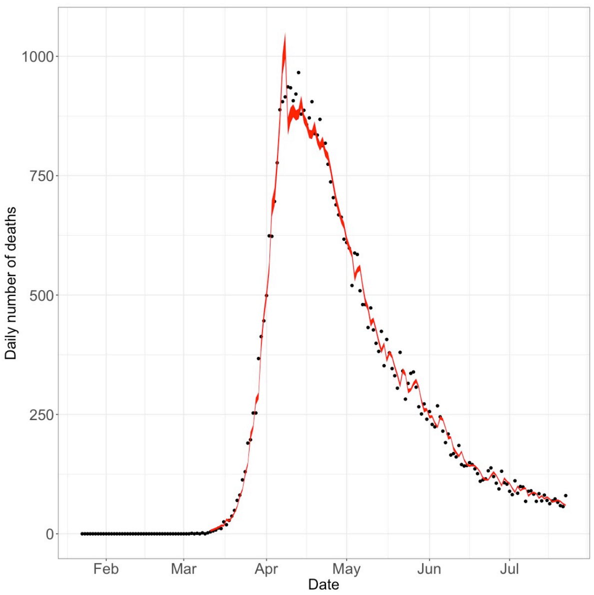

(a)

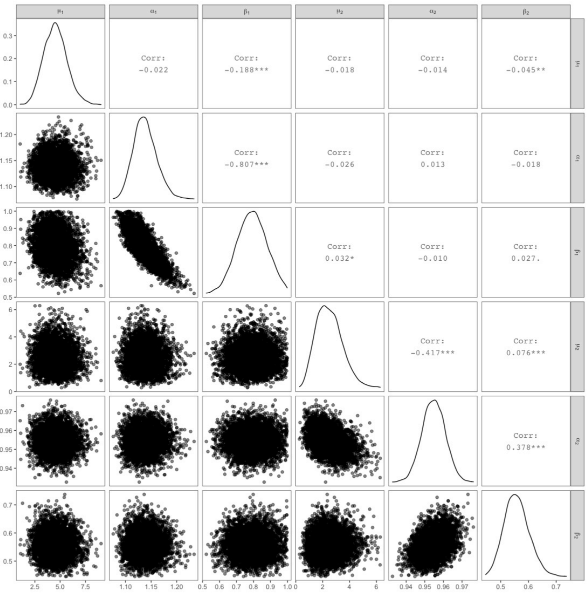

(b)

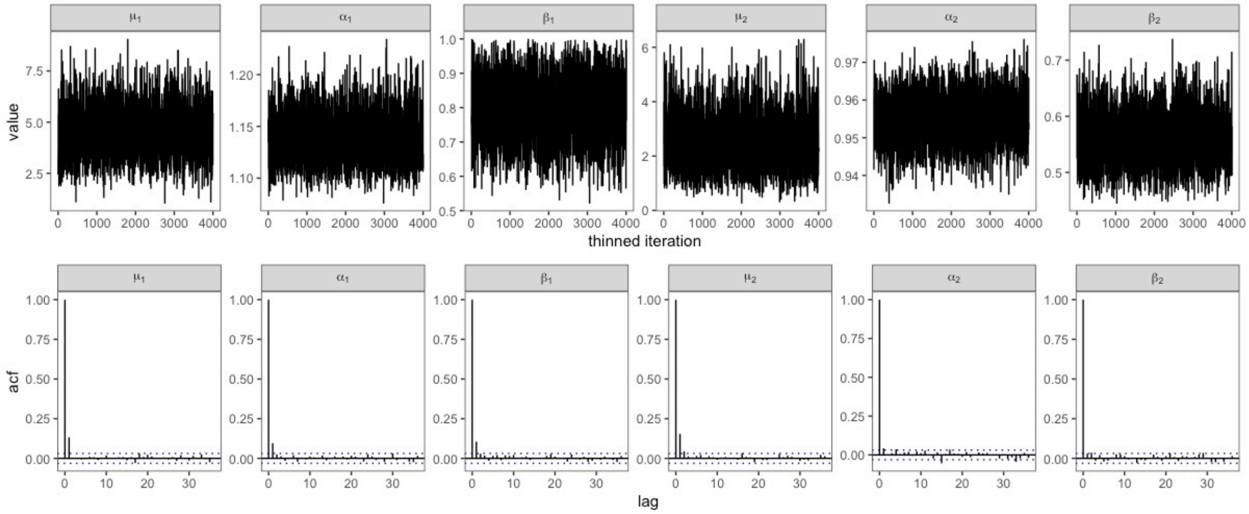

(c)

Fig 7. Convergence and diagnostic plots for the U.K. Top left hand panel: compares the observed number of deaths (black dots) with the 95% posterior interval for the estimated expected number of events (solid red ribbon). Top right hand panel: shows pairwise correlation between all parameters in the lower triangle, corresponding correlation values in the upper triangle, and the marginal posterior densities for each parameter on the diagonal. Bottom panel: shows trace plots on the top row and the autocorrelation function on the bottom row for each parameter. All figures were generated after thinning the posterior samples.

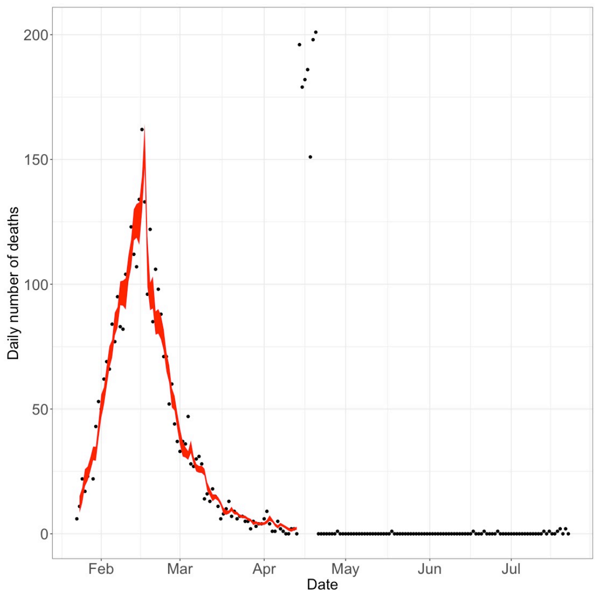

(a)

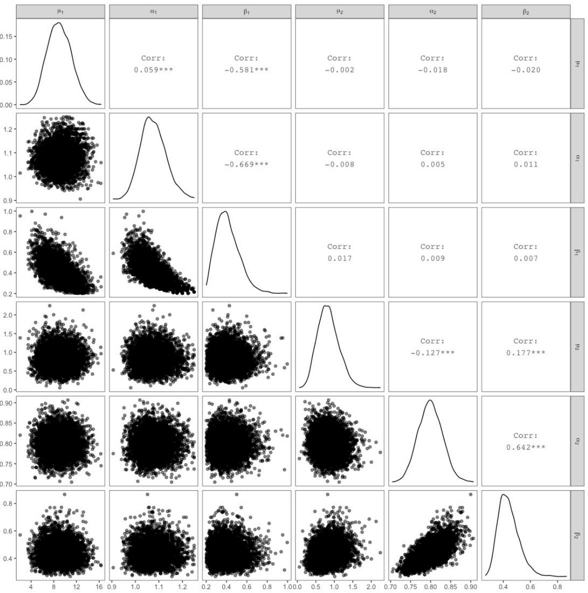

(b)

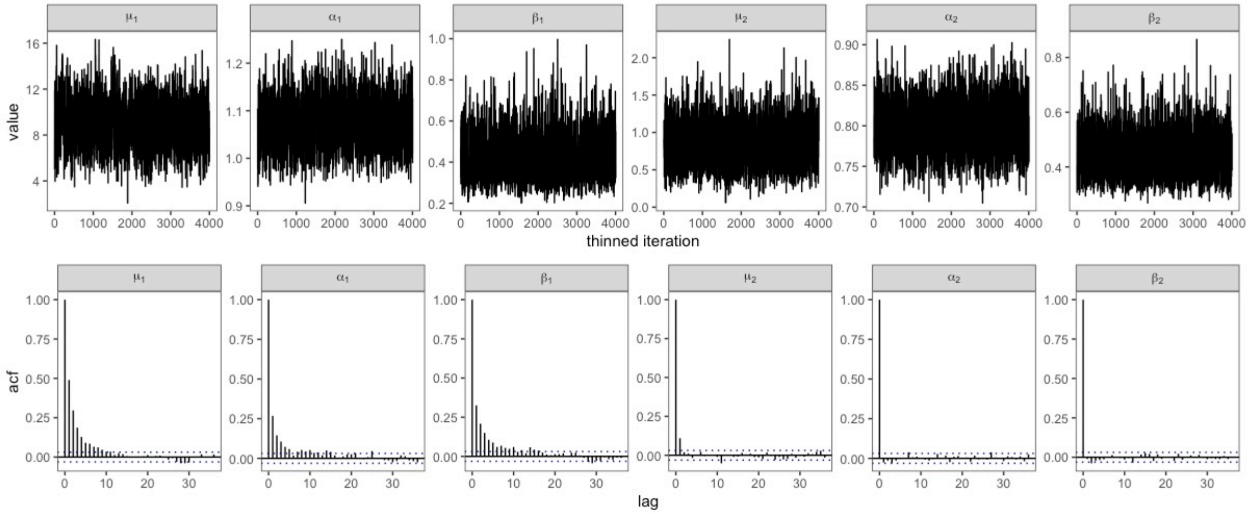

(c)

Fig 8. Convergence and diagnostic plots for China. Top left hand panel: compares the observed number of deaths (black dots) with the 95% posterior interval for the estimated expected number of events (solid red ribbon). Top right hand panel: shows pairwise correlation between all parameters in the lower triangle, corresponding correlation values in the upper triangle, and the marginal posterior densities for each parameter on the diagonal. Bottom panel: shows trace plots on the top row and the autocorrelation function on the bottom row for each parameter. All figures were generated after thinning the posterior samples.

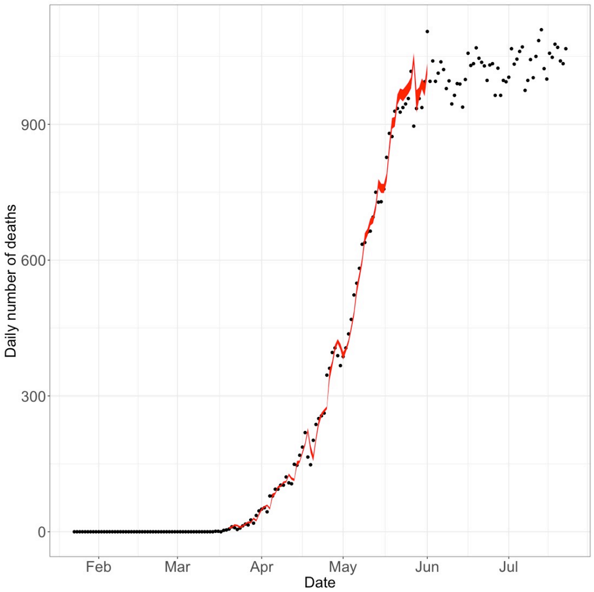

(a)

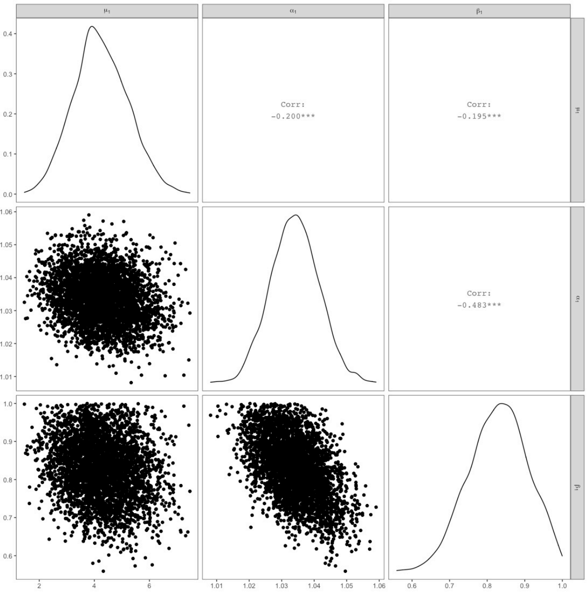

(b)

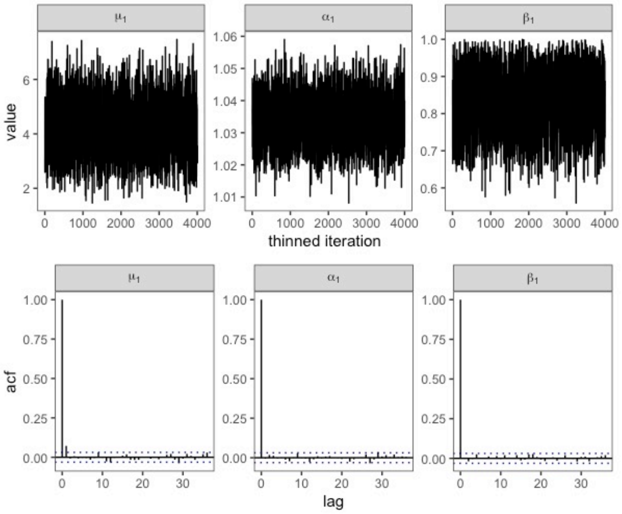

(c)

Fig 9. Convergence and diagnostic plots for Brazil. Top left hand panel: compares the observed number of deaths (black dots) with the 95% posterior interval for the estimated expected number of events (solid red ribbon). Top right hand panel: shows pairwise correlation between all parameters in the lower triangle, corresponding correlation values in the upper triangle, and the marginal posterior densities for each parameter on the diagonal. Bottom panel: shows trace plots on the top row and the autocorrelation function on the bottom row for each parameter. All figures were generated after thinning the posterior samples.

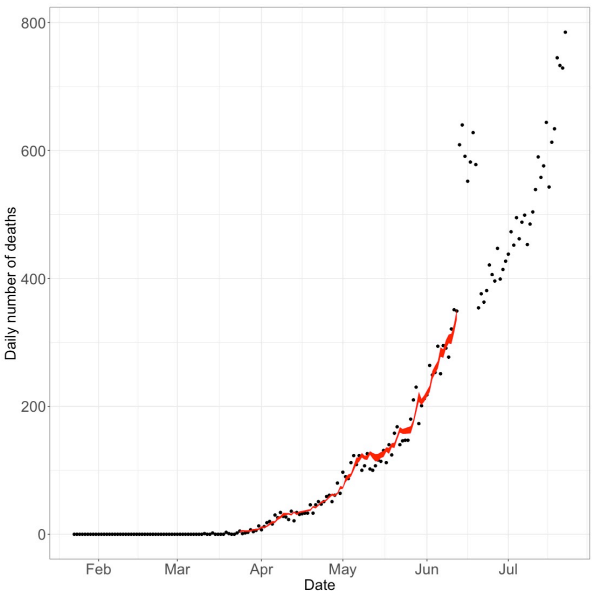

(a)

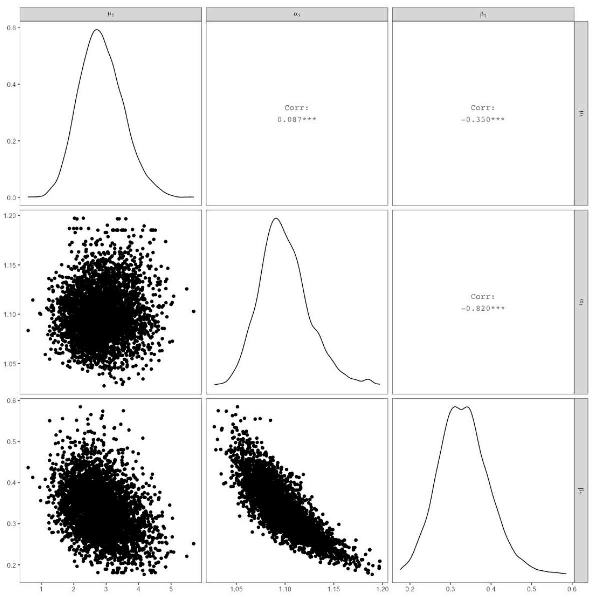

(b)

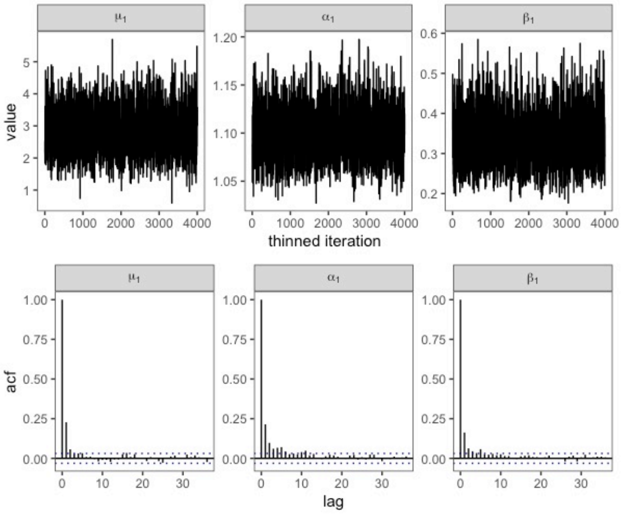

(c)

Fig 10. Convergence and diagnostic plots for India. Top left hand panel: compares the observed number of deaths (black dots) with the 95% posterior interval for the estimated expected number of events (solid red ribbon). Top right hand panel: shows pairwise correlation between all parameters in the lower triangle, corresponding correlation values in the upper triangle, and the marginal posterior densities for each parameter on the diagonal. Bottom panel: shows trace plots on the top row and the autocorrelation function on the bottom row for each parameter. All figures were generated after thinning the posterior samples.

Subsequent analysis

We now present similar plots for the analysis of the subsequent phases, where $\mu_1, \mu_2 \sim \text{Gamma}(5, 1)$.

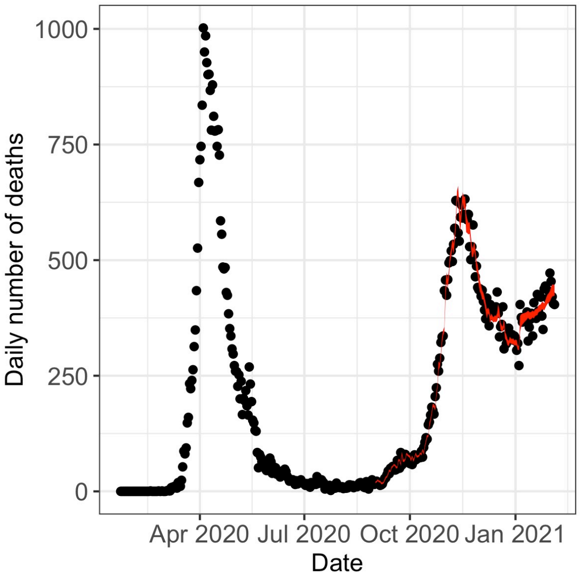

(a)

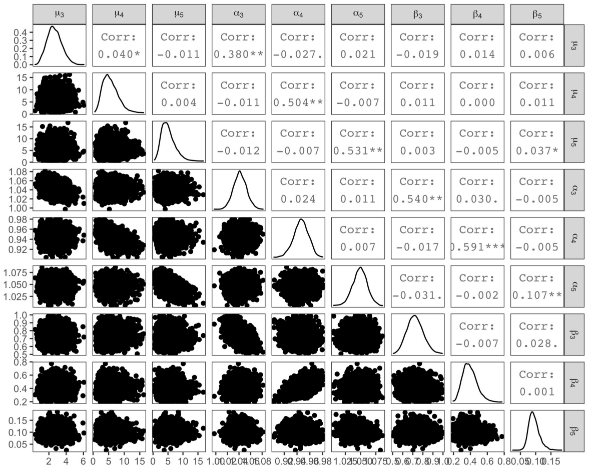

(b)

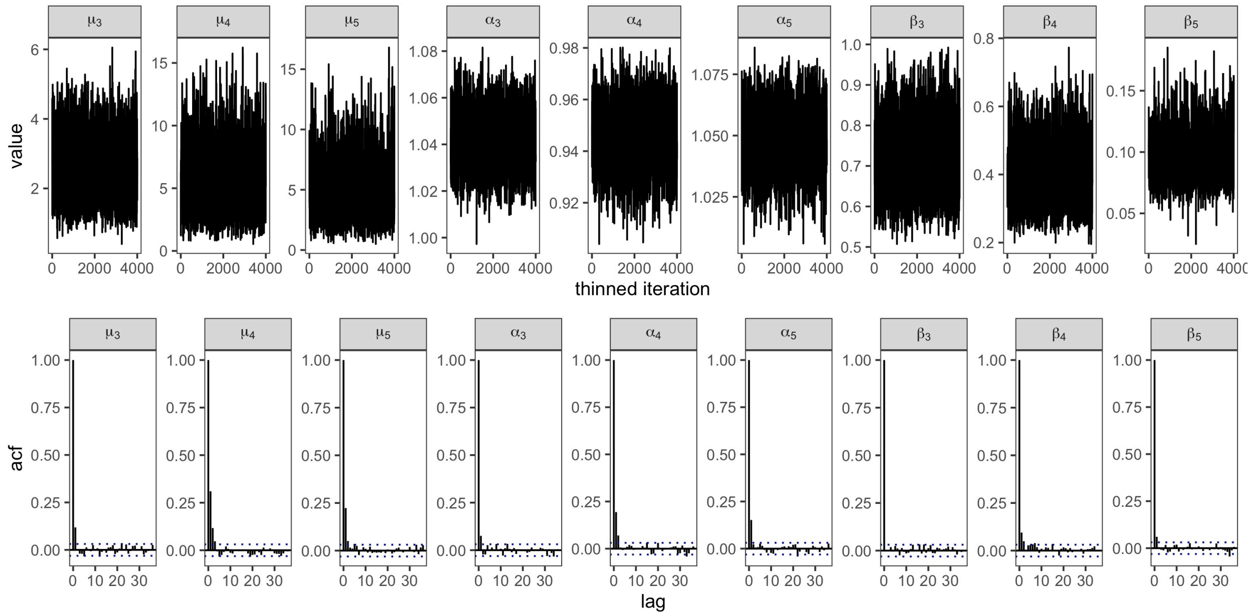

(c)

Fig 11. Convergence and diagnostic plots for France. Top left hand panel: compares the observed number of deaths (black dots) with the 95% posterior interval for the estimated expected number of events (solid red ribbon). Top right hand panel: shows pairwise correlation between all parameters in the lower triangle, corresponding correlation values in the upper triangle, and the marginal posterior densities for each parameter on the diagonal. Bottom panel: shows trace plots on the top row and the autocorrelation function on the bottom row for each parameter. All figures were generated after thinning the posterior samples.

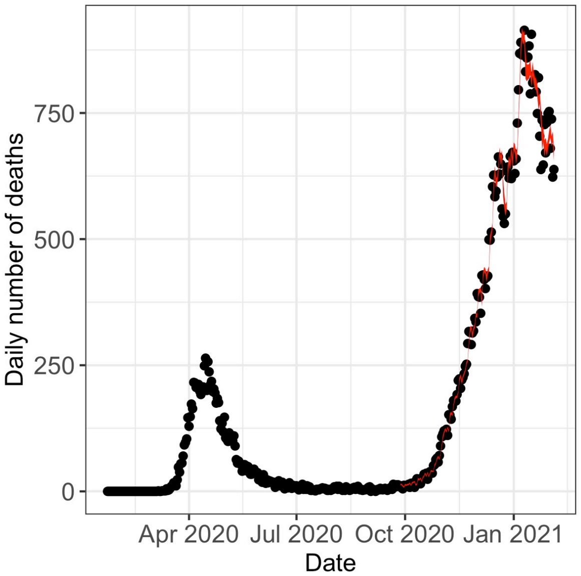

(a)

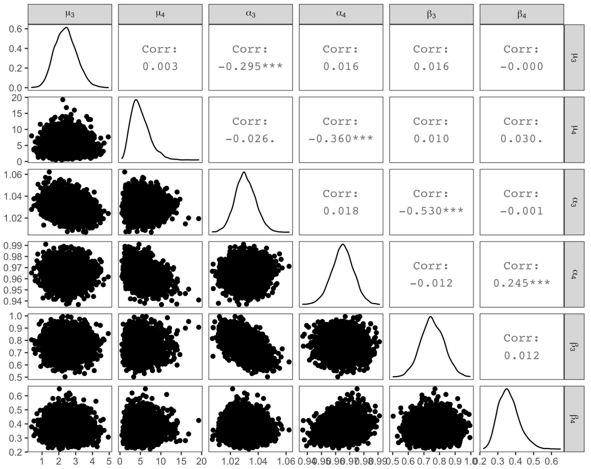

(b)

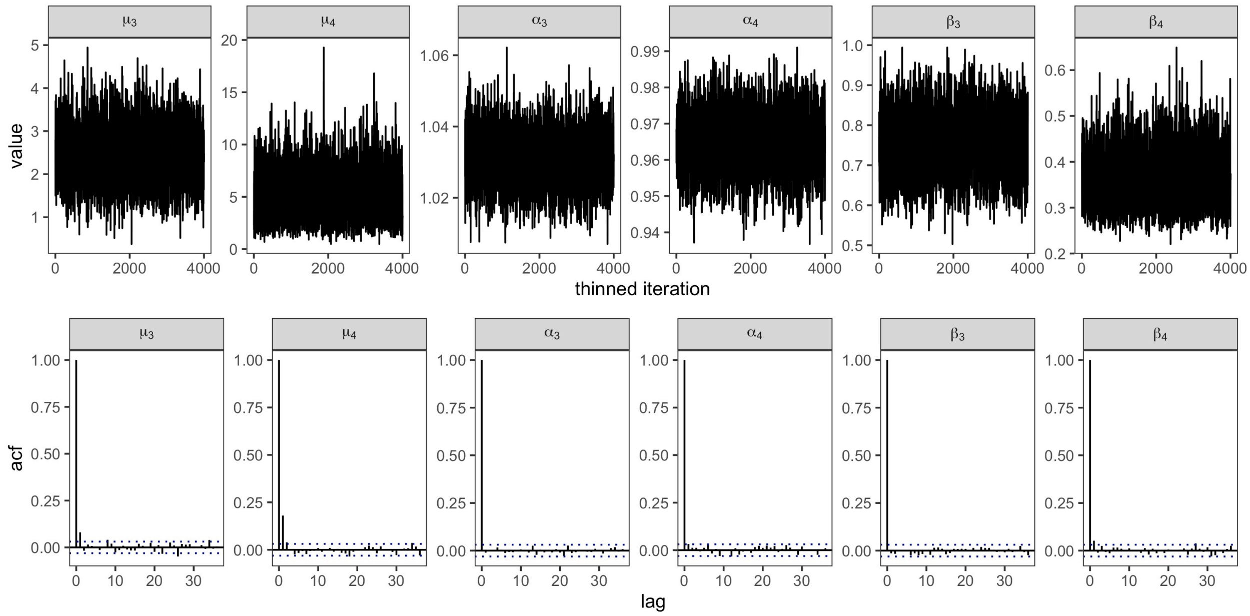

(c)

Fig 12. Convergence and diagnostic plots for Germany. Top left hand panel: compares the observed number of deaths (black dots) with the 95% posterior interval for the estimated expected number of events (solid red ribbon). Top right hand panel: shows pairwise correlation between all parameters in the lower triangle, corresponding correlation values in the upper triangle, and the marginal posterior densities for each parameter on the diagonal. Bottom panel: shows trace plots on the top row and the autocorrelation function on the bottom row for each parameter. All figures were generated after thinning the posterior samples.

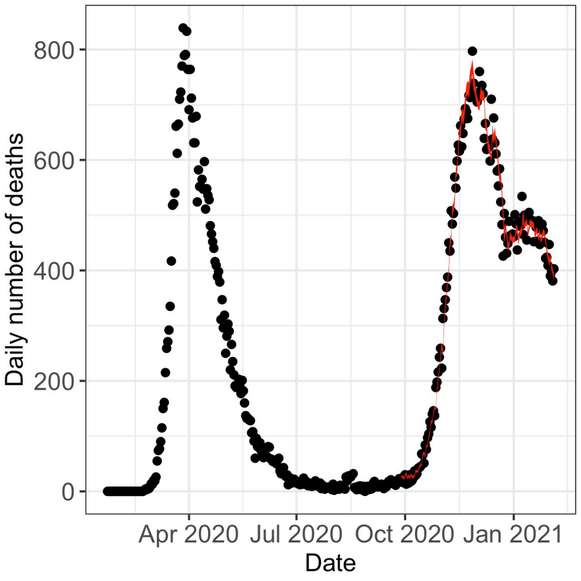

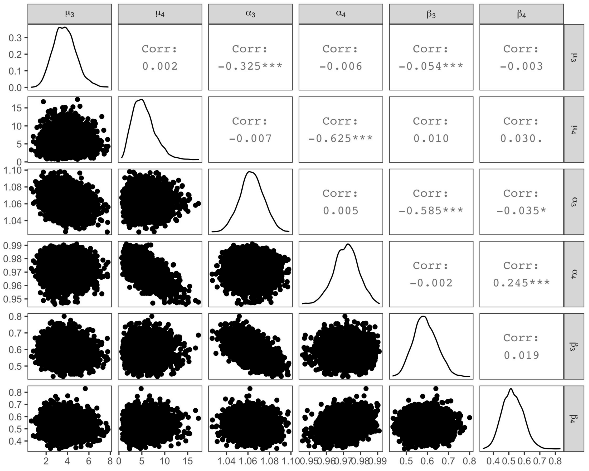

(a) **(b)**

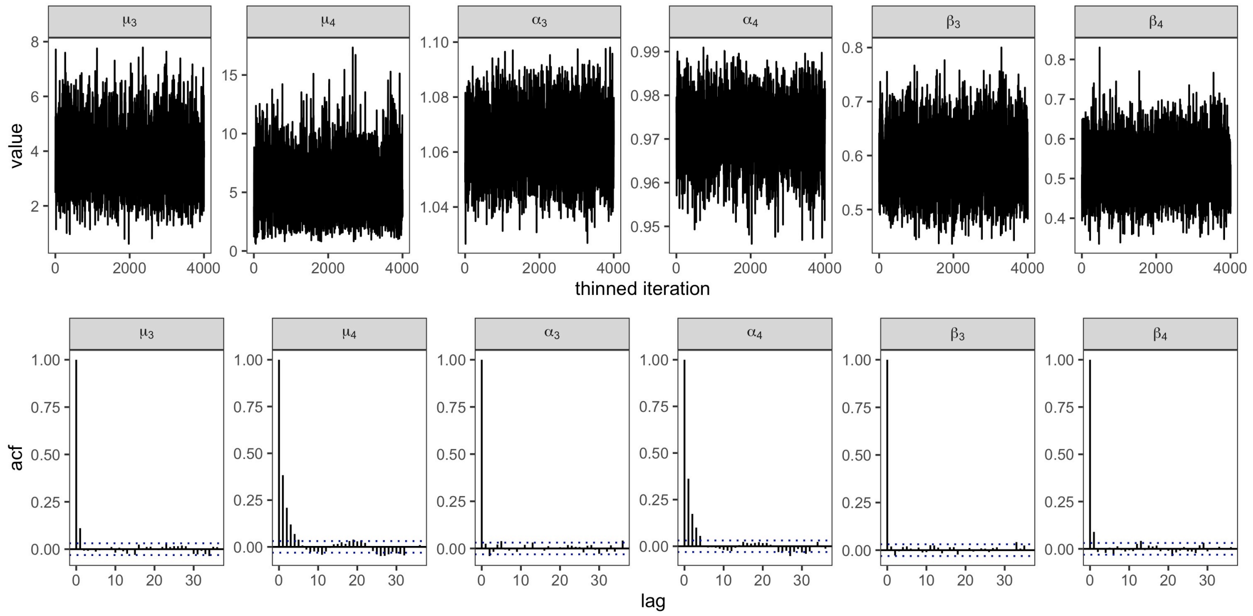

(c)

Fig 13. Convergence and diagnostic plots for Italy. Top left hand panel: compares the observed number of deaths (black dots) with the 95% posterior interval for the estimated expected number of events (solid red ribbon). Top right hand panel: shows pairwise correlation between all parameters in the lower triangle, corresponding correlation values in the upper triangle, and the marginal posterior densities for each parameter on the diagonal. Bottom panel: shows trace plots on the top row and the autocorrelation function on the bottom row for each parameter. All figures were generated after thinning the posterior samples.

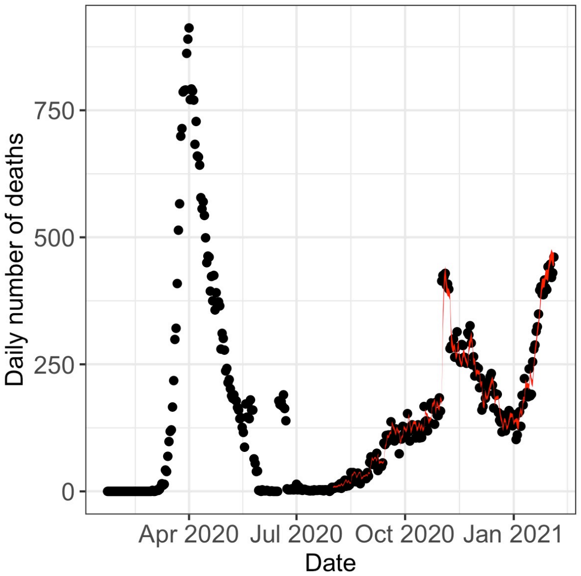

(a)

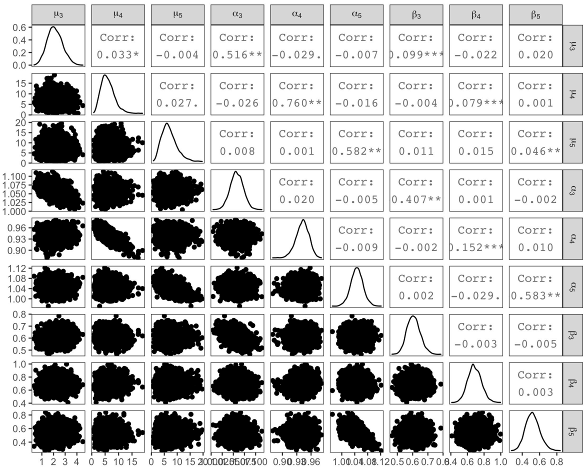

(b)

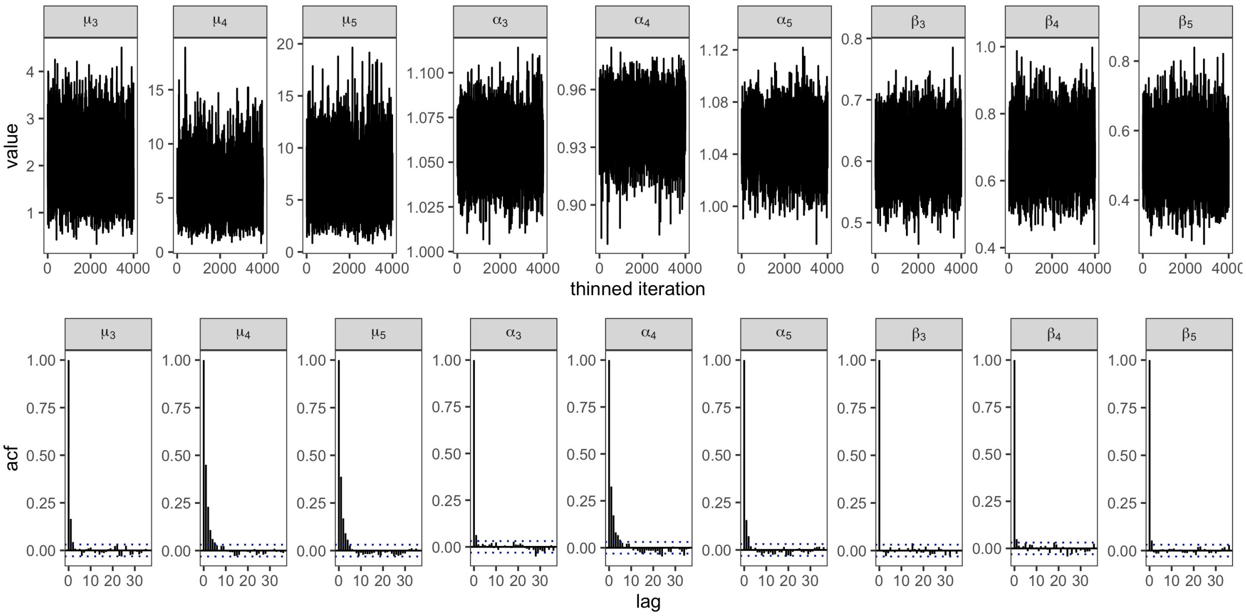

(c)

Fig 14. Convergence and diagnostic plots for Spain. Top left hand panel: compares the observed number of deaths (black dots) with the 95% posterior interval for the estimated expected number of events (solid red ribbon). Top right hand panel: shows pairwise correlation between all parameters in the lower triangle, corresponding correlation values in the upper triangle, and the marginal posterior densities for each parameter on the diagonal. Bottom panel: shows trace plots on the top row and the autocorrelation function on the bottom row for each parameter. All figures were generated after thinning the posterior samples.

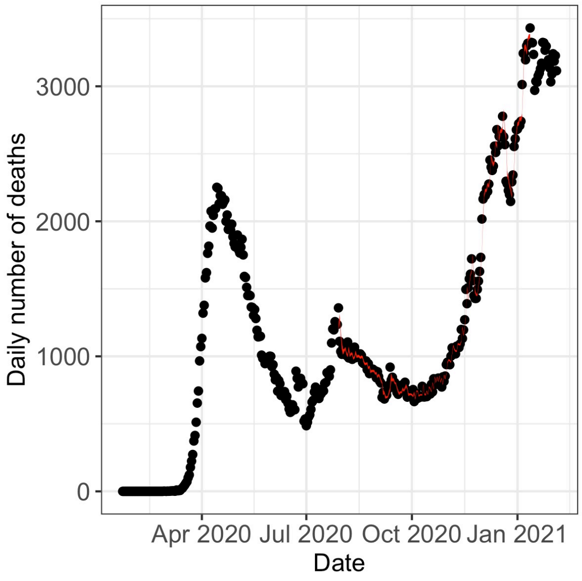

(a)

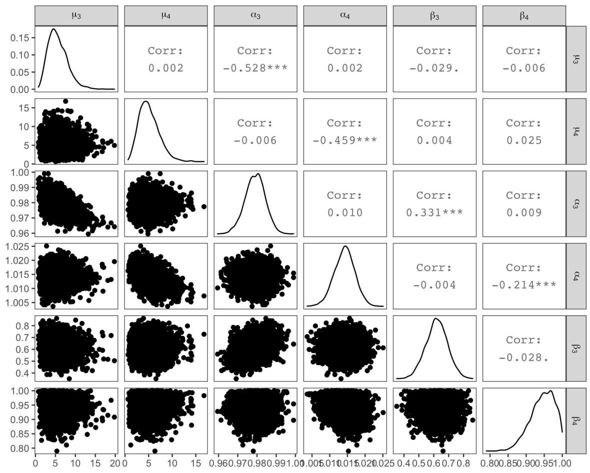

(b)

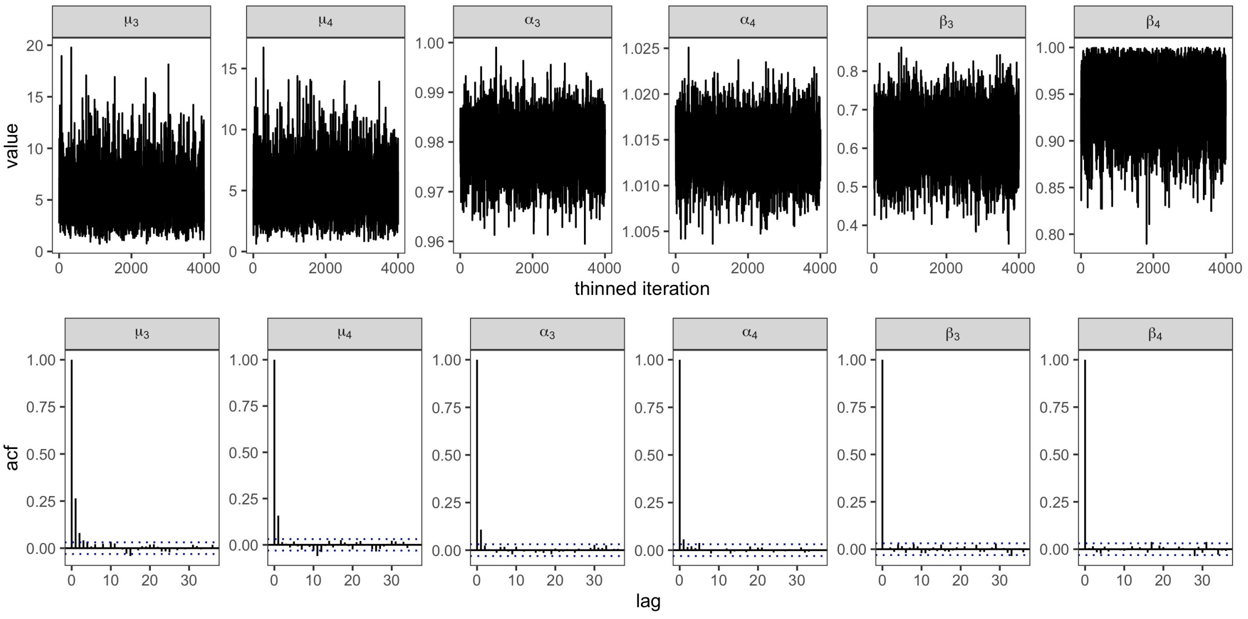

(c)

Fig 15. Convergence and diagnostic plots for the U.S. Top left hand panel: compares the observed number of deaths (black dots) with the 95% posterior interval for the estimated expected number of events (solid red ribbon). Top right hand panel: shows pairwise correlation between all parameters in the lower triangle, corresponding correlation values in the upper triangle, and the marginal posterior densities for each parameter on the diagonal. Bottom panel: shows trace plots on the top row and the autocorrelation function on the bottom row for each parameter. All figures were generated after thinning the posterior samples.

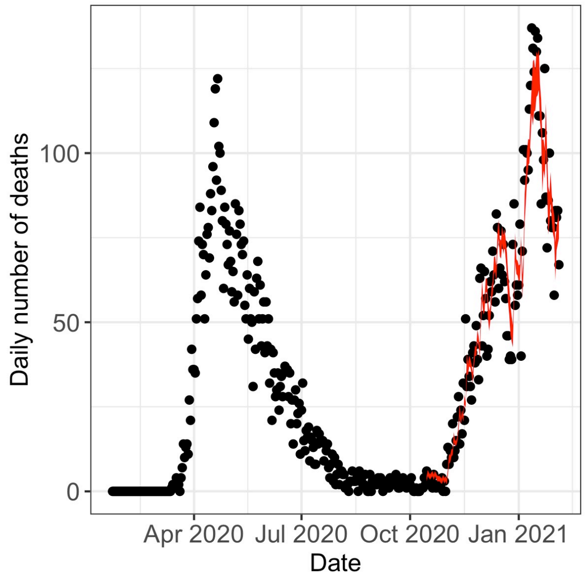

(a)

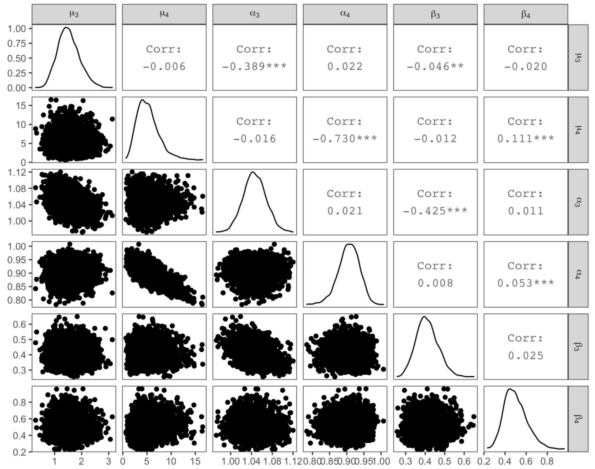

(b)

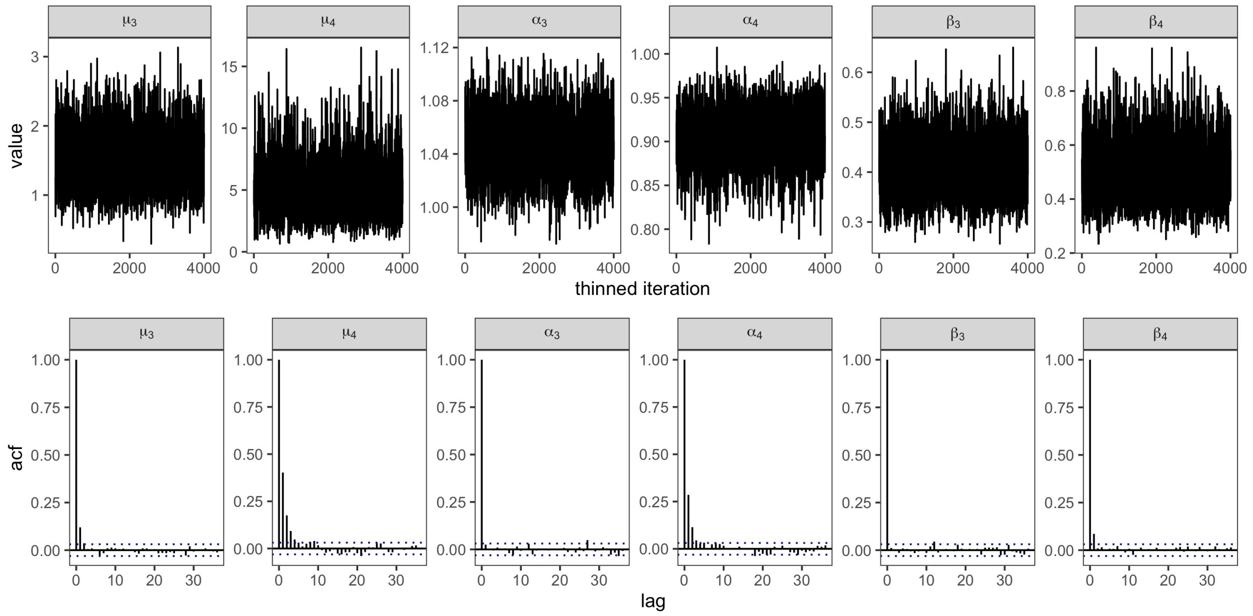

(c)

Fig 16. Convergence and diagnostic plots for Sweden. Top left hand panel: compares the observed number of deaths (black dots) with the 95% posterior interval for the estimated expected number of events (solid red ribbon). Top right hand panel: shows pairwise correlation between all parameters in the lower triangle, corresponding correlation values in the upper triangle, and the marginal posterior densities for each parameter on the diagonal. Bottom panel: shows trace plots on the top row and the autocorrelation function on the bottom row for each parameter. All figures were generated after thinning the posterior samples.

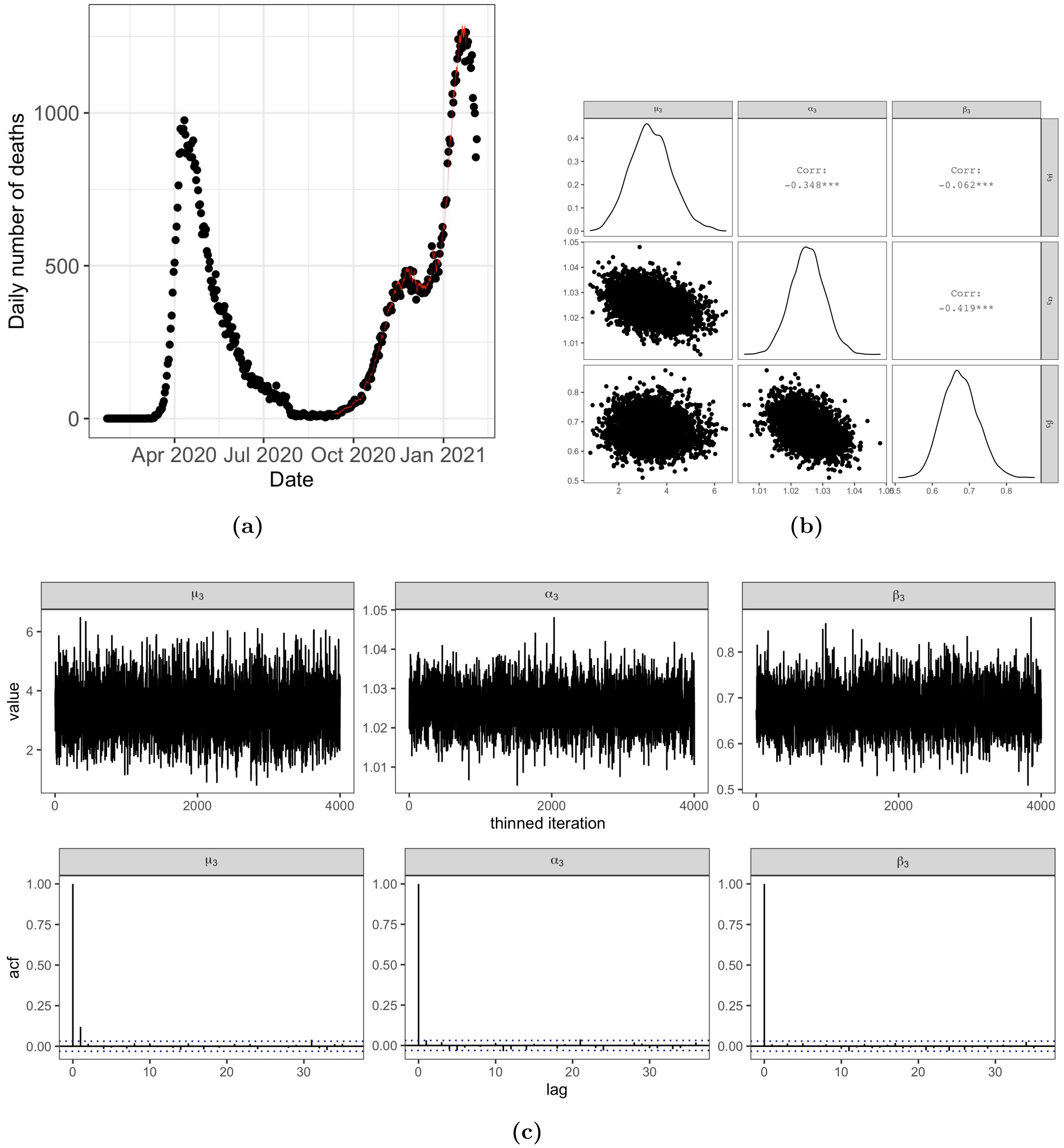

Fig 17. Convergence and diagnostic plots for the U.K. Top left hand panel: compares the observed number of deaths (black dots) with the 95% posterior interval for the estimated expected number of events (solid red ribbon). Top right hand panel: shows pairwise correlation between all parameters in the lower triangle, corresponding correlation values in the upper triangle, and the marginal posterior densities for each parameter on the diagonal. Bottom panel: shows trace plots on the top row and the autocorrelation function on the bottom row for each parameter. All figures were generated after thinning the posterior samples.

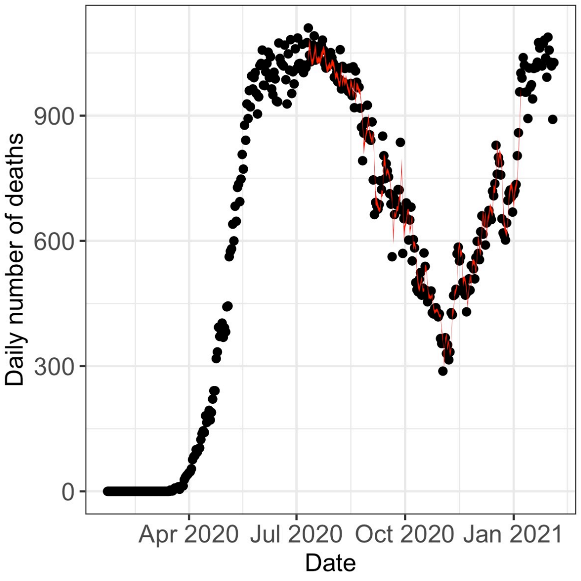

(a)

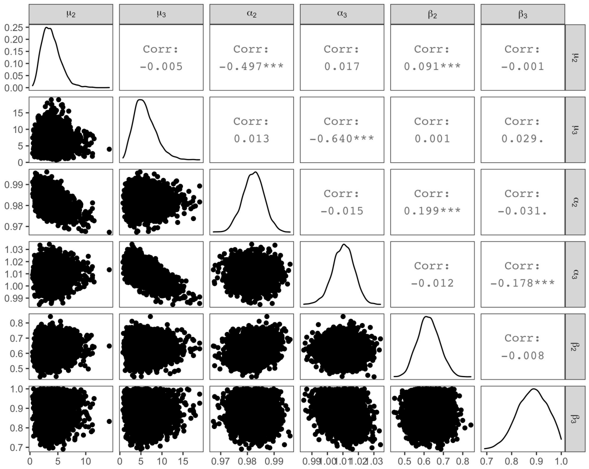

(b)

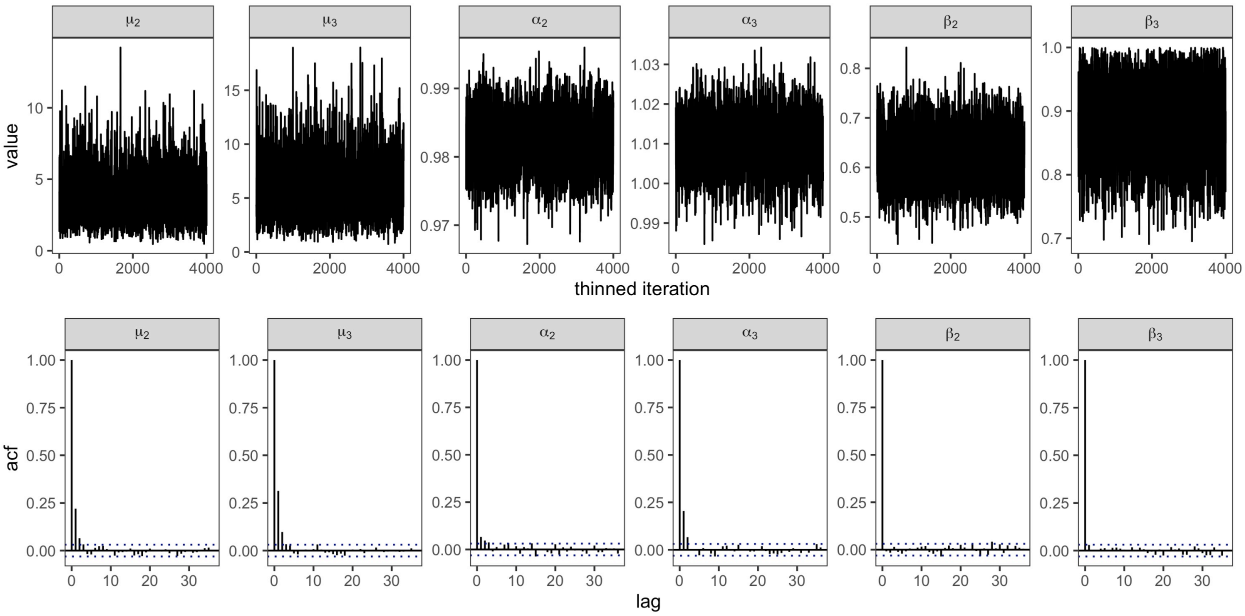

(c)

Fig 18. Convergence and diagnostic plots for Brazil. Top left hand panel: compares the observed number of deaths (black dots) with the 95% posterior interval for the estimated expected number of events (solid red ribbon). Top right hand panel: shows pairwise correlation between all parameters in the lower triangle, corresponding correlation values in the upper triangle, and the marginal posterior densities for each parameter on the diagonal. Bottom panel: shows trace plots on the top row and the autocorrelation function on the bottom row for each parameter. All figures were generated after thinning the posterior samples.

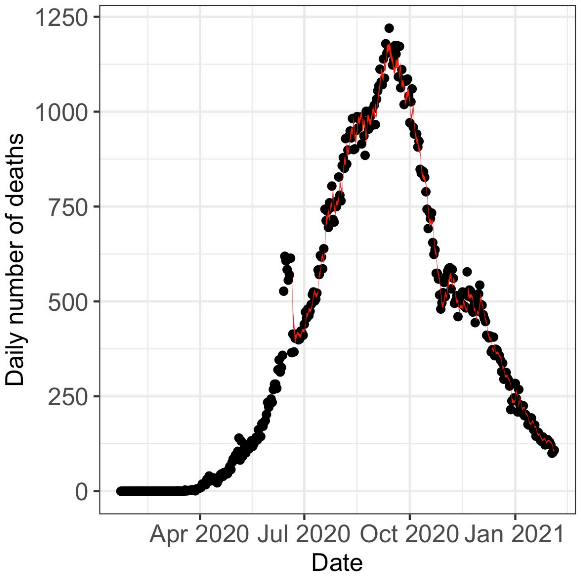

(a)

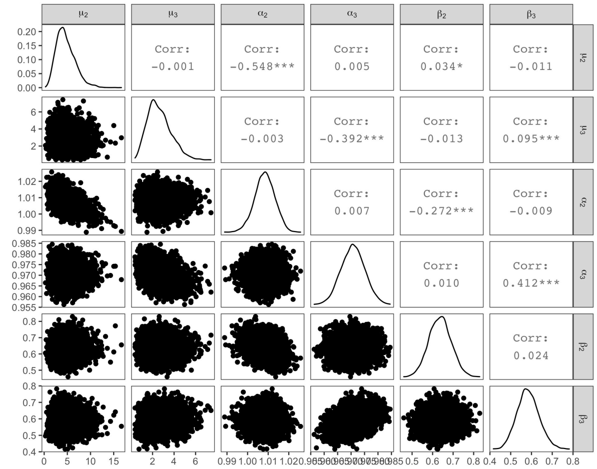

(b)

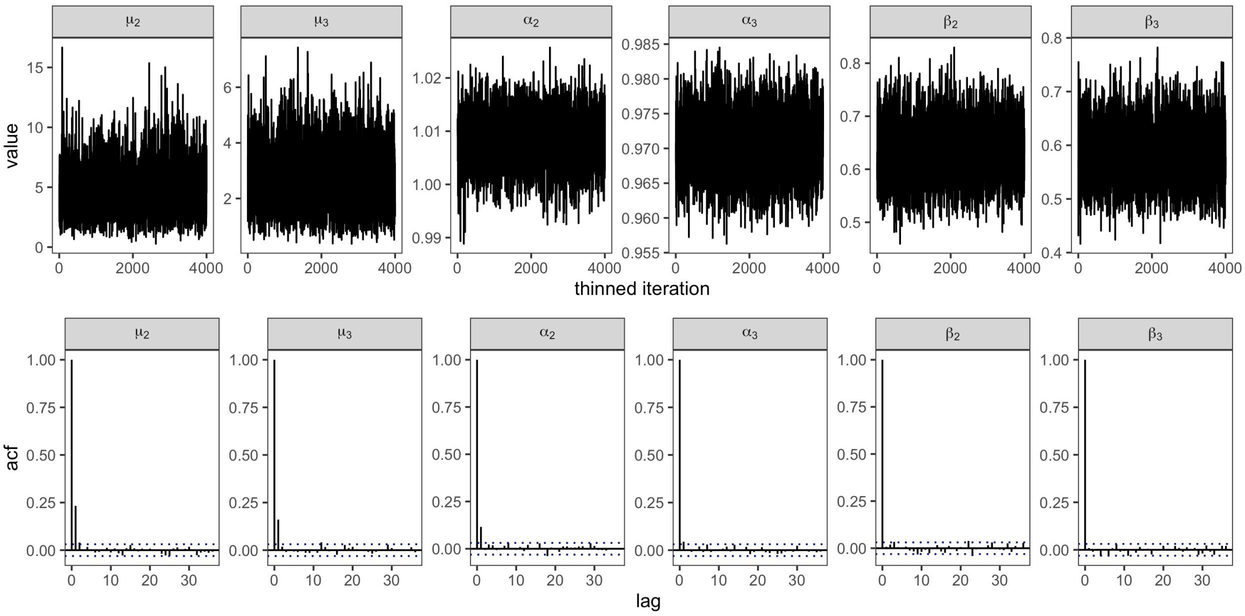

(c)

Fig 19. Convergence and diagnostic plots for India. Top left hand panel: compares the observed number of deaths (black dots) with the 95% posterior interval for the estimated expected number of events (solid red ribbon). Top right hand panel: shows pairwise correlation between all parameters in the lower triangle, corresponding correlation values in the upper triangle, and the marginal posterior densities for each parameter on the diagonal. Bottom panel: shows trace plots on the top row and the autocorrelation function on the bottom row for each parameter. All figures were generated after thinning the posterior samples.
