# Supplementary material for: Simple discrete-time self-exciting models can describe complex dynamic processes: A case study of COVID-19
Source: PLoS One. 2021 Apr 9;16(4):e0250015. doi: 10.1371/journal.pone.0250015 (PMC8034752; doi:10.1371/journal.pone.0250015)
Supplement: S4 Appendix — Phase 1 versus Phase 2 median and 80% intervals of baseline parameters for countries with two phases. (PDF) [file pone.0250015.s004.pdf]

S4 Appendix: Parameter estimates of baseline parameters

Table 1. Phase 1 versus Phase 2 median and 80% intervals of baseline parameters for countries with two phases

Country	Prior	μ_1	μ_2
Italy	logN(1,1)	3.52 (2.13,4.99)	0.34 (0.12,0.78)
	logN(5,1.5)	4.04 (2.62,5.59)	0.33 (0.09,0.85)
	Gamma(2,2)	4.11 (2.87,5.56)	0.66 (0.23,1.3)
	Gamma(5,1)	4.39 (3.18,5.71)	1.17 (0.69,1.8)
	Uniform	4.46 (3.08,5.99)	0.41 (0.07,1.06)
France	logN(1,1)	3.91 (2.64,5.37)	0.55 (0.19,1.21)
	logN(5,1.5)	4.33 (3.04,5.82)	0.67 (0.17,1.44)
	Gamma(2,2)	4.38 (3.15,5.79)	1.1 (0.47,1.84)
	Gamma(5,1)	4.57 (3.38,5.91)	1.57 (0.97,2.28)
	Uniform	4.72 (3.44,6.21)	0.9 (0.23,1.73)
Spain	logN(1,1)	4.84 (2.78,7.11)	0.18 (0.08,0.37)
	logN(5,1.5)	5.78 (3.72,8)	0.16 (0.05,0.35)
	Gamma(2,2)	5.71 (3.83,7.68)	0.24 (0.09,0.48)
	Gamma(5,1)	5.78 (4.06,7.6)	0.49 (0.28,0.76)
	Uniform	6.5 (4.45,8.78)	0.14 (0.03,0.36)
Germany	logN(1,1)	2.97 (1.49,4.61)	0.35 (0.13,0.74)
	logN(5,1.5)	3.68 (2.06,5.3)	0.35 (0.1,0.77)
	Gamma(2,2)	3.85 (2.4,5.39)	0.58 (0.24,1.06)
	Gamma(5,1)	4.17 (2.89,5.54)	0.95 (0.59,1.39)
	Uniform	4.16 (2.67,5.76)	0.4 (0.09,0.9)
Sweden	logN(1,1)	3.05 (1.8,4.5)	0.44 (0.15,1.12)
	logN(5,1.5)	3.65 (2.28,5.18)	0.52 (0.12,1.38)
	Gamma(2,2)	3.78 (2.49,5.26)	1.07 (0.38,2.03)
	Gamma(5,1)	4.05 (2.88,5.44)	1.79 (1.05,2.68)
	Uniform	4.05 (2.67,5.61)	0.72 (0.14,1.78)
U.K.	logN(1,1)	3.22 (1.57,5.05)	0.46 (0.14,1.29)
	logN(5,1.5)	3.98 (2.32,5.78)	0.59 (0.13,1.8)
	Gamma(2,2)	4.15 (2.68,5.8)	1.39 (0.48,2.87)
	Gamma(5,1)	4.51 (3.08,6)	2.42 (1.32,3.75)
	Uniform	4.69 (3.02,6.51)	1.06 (0.2,2.67)
U.S.	logN(1,1)	3.56 (2.56,4.72)	0.57 (0.16,1.95)
	logN(5,1.5)	3.82 (2.77,4.94)	1.04 (0.18,4.41)
	Gamma(2,2)	3.91 (2.86,5.06)	2.71 (0.87,6.31)
	Gamma(5,1)	4.08 (3.13,5.15)	4.1 (2.16,7.12)
	Uniform	4.08 (3.01,5.26)	4.59 (0.82,12.99)
China	logN(1,1)	9.76 (6.32,13.27)	0.29 (0.11,0.58)
	logN(5,1.5)	11.24 (7.97,14.49)	0.28 (0.08,0.62)
	Gamma(2,2)	9.75 (6.81,12.81)	0.46 (0.18,0.85)
	Gamma(5,1)	8.92 (6.29,11.73)	0.82 (0.48,1.22)
	Uniform	12.44 (9.07,15.85)	0.29 (0.06,0.7)

Continued on next page

Table 1 – *Continued from previous page*

Country	Prior	μ_1	μ_2
Brazil	logN(1,1)	3.31 (1.81,4.74)	
	logN(5,1.5)	3.78 (2.37,5.26)	
	Gamma(2,2)	3.97 (2.67,5.37)	
	Gamma(5,1)	4.18 (2.98,5.52)	
	Uniform	4.24 (2.83,5.7)	
India	logN(1,1)	2 (1.11,2.96)	
	logN(5,1.5)	2.31 (1.41,3.29)	
	Gamma(2,2)	2.52 (1.66,3.46)	
	Gamma(5,1)	2.81 (2.02,3.72)	
	Uniform	2.55 (1.66,3.57)	

We only alter the prior choice for the baseline parameters μ. Thus, we do not present the posterior medians and posterior credible intervals corresponding to the self-exciting parameters α and β for each of the prior choices of μ. Their respective priors remained unchanged throughout our analysis, and their posterior distributions are very similar to those presented in the main article.
