# Supplementary material for: Simple discrete-time self-exciting models can describe complex dynamic processes: A case study of COVID-19
Source: PLoS One. 2021 Apr 9;16(4):e0250015. doi: 10.1371/journal.pone.0250015 (PMC8034752; doi:10.1371/journal.pone.0250015)
Supplement: S5 Appendix — Tables containing number of missing data points with actual value within 80% and 95% posterior interval, for all prior choices. (PDF) [file pone.0250015.s005.pdf]

S5 Appendix: Missing data interpolation.

Country	Run 1	Run 2	Run 3	Run 4	Run 5	Average
France	10/14	11/14	8/14	12/14	12/14	10.6/14
Italy	14/15	14/15	12/15	15/15	11/15	13.2/15
Germany	11/14	13/14	14/14	14/14	12/14	12.8/14
Spain	6/11	8/11	9/11	7/11	10/11	8/11
Sweden	13/13	13/13	12/13	13/13	11/13	12.4/13
U.K.	12/14	12/14	10/14	12/14	14/14	12/14
China	8/9	8/9	9/9	9/9	9/9	8.6/9
U.S.	8/11	9/11	11/11	7/11	7/11	8.4/11
Brazil	6/8	7/8	5/8	7/8	8/8	6.6/8
India	8/9	6/9	9/9	8/9	8/9	7.8/9

Table 1. $\log N(1,1)$: number of missing data points with actual value within 95% posterior interval

Country	Run 1	Run 2	Run 3	Run 4	Run 5	Average
France	9/14	6/14	6/14	8/14	9/14	7.6/14
Italy	12/15	12/15	11/15	10/15	8/15	10.6/15
Germany	9/14	11/14	13/14	13/14	8/14	10.8/14
Spain	5/11	4/11	7/11	7/11	8/11	6.2/11
Sweden	12/13	11/13	10/13	11/13	10/13	10.8/13
U.K.	9/14	11/14	7/14	10/14	10/14	9.4/14
China	7/9	7/9	7/9	6/9	9/9	7.2/9
U.S.	5/11	5/11	7/11	3/11	6/11	5.2/11
Brazil	5/8	6/8	4/8	3/8	4/8	4.4/8
India	6/9	4/9	8/9	8/9	8/9	6.8/9

Table 2. $\log N(1,1)$: number of missing data points with actual value within 80% posterior interval

Country	Run 1	Run 2	Run 3	Run 4	Run 5	Average
France	11/14	11/14	8/14	12/14	12/14	10.8/14
Italy	14/15	14/15	12/15	15/15	11/15	13.2/15
Germany	12/14	13/14	14/14	14/14	13/14	13.2/14
Spain	6/11	8/11	9/11	7/11	10/11	8/11
Sweden	13/13	13/13	12/13	13/13	11/13	12.4/13
U.K.	12/14	12/14	10/14	12/14	14/14	12/14
China	8/9	8/9	9/9	9/9	9/9	8.6/9
U.S.	8/11	8/11	11/11	7/11	8/11	8.4/11
Brazil	6/8	7/8	5/8	7/8	8/8	6.6/8
India	8/9	6/9	9/9	8/9	8/9	7.8/9

Table 3. $\log N(5,1.5)$: number of missing data points with actual value within 95% posterior interval

Country	Run 1	Run 2	Run 3	Run 4	Run 5	Average
France	8/14	6/14	6/14	8/14	9/14	7.4/14
Italy	12/15	12/15	11/15	11/15	8/15	10.8/15
Germany	8/14	10/14	12/14	13/14	8/14	10.2/14
Spain	5/11	4/11	7/11	6/11	8/11	6/11
Sweden	12/13	11/13	10/13	11/13	10/13	10.8/13
U.K.	9/14	11/14	6/14	9/14	10/14	9/14
China	7/9	7/9	7/9	7/9	9/9	7.4/9
U.S.	5/11	5/11	7/11	3/11	6/11	5.2/11
Brazil	5/8	6/8	4/8	4/8	4/8	4.6/8
India	6/9	4/9	8/9	8/9	8/9	6.8/9

Table 4. $\log N(5, 1.5)$: number of missing data points with actual value within 80% posterior interval

Country	Run 1	Run 2	Run 3	Run 4	Run 5	Average
France	11/14	10/14	8/14	13/14	12/14	10.8/14
Italy	14/15	14/15	12/15	15/15	10/15	13/15
Germany	12/14	14/14	14/14	14/14	12/14	13.2/14
Spain	6/11	8/11	9/11	7/11	10/11	8/11
Sweden	13/13	13/13	13/13	13/13	11/13	12.6/13
U.K.	12/14	12/14	10/14	12/14	14/14	12/14
China	8/9	8/9	9/9	9/9	9/9	8.6/9
U.S.	8/11	8/11	11/11	7/11	7/11	8.2/11
Brazil	6/8	8/8	5/8	7/8	8/8	6.8/8
India	8/9	6/9	9/9	8/9	8/9	7.8/9

Table 5. Gamma(2,2): number of missing data points with actual value within 95% posterior interval

Country	Run 1	Run 2	Run 3	Run 4	Run 5	Average
France	8/14	6/14	6/14	8/14	9/14	7.4/14
Italy	12/15	12/15	11/15	10/15	8/15	10.6/15
Germany	8/14	10/14	12/14	13/14	8/14	10.2/14
Spain	5/11	4/11	6/11	6/11	8/11	5.8/11
Sweden	12/13	10/13	10/13	10/13	10/13	10.4/13
U.K.	9/14	11/14	6/14	10/14	10/14	9.2/14
China	7/9	7/9	7/9	6/9	9/9	7.2/9
U.S.	5/11	5/11	7/11	3/11	6/11	5.2/11
Brazil	5/8	6/8	4/8	4/8	4/8	4.6/8
India	6/9	4/9	8/9	8/9	8/9	6.8/9

Table 6. Gamma(2,2): number of missing data points with actual value within 80% posterior interval

Country	Run 1	Run 2	Run 3	Run 4	Run 5	Average
France	11/14	11/14	9/14	12/14	12/14	11/14
Italy	14/15	14/15	12/15	15/15	10/15	13/15
Germany	12/14	14/14	14/14	14/14	13/14	13.4/14
Spain	6/11	8/11	9/11	7/11	10/11	8/11
Sweden	13/13	13/13	13/13	13/13	11/13	12.6/13
U.K.	12/14	12/14	10/14	12/14	13/14	11.8/14
China	8/9	8/9	9/9	9/9	9/9	8.6/9
U.S.	8/11	9/11	11/11	7/11	8/11	8.6/11
Brazil	6/8	7/8	5/8	7/8	8/8	6.6/8
India	8/9	6/9	9/9	8/9	8/9	7.8/9

Table 7. Gamma(5,1): number of missing data points with actual value within 95% posterior interval

Country	Run 1	Run 2	Run 3	Run 4	Run 5	Average
France	8/14	6/14	6/14	9/14	9/14	7.6/14
Italy	12/15	12/15	11/15	10/15	9/15	10.8/15
Germany	10/14	10/14	12/14	14/14	8/14	10.8/14
Spain	5/11	4/11	7/11	6/11	8/11	6/11
Sweden	12/13	10/13	10/13	10/13	10/13	10.4/13
U.K.	9/14	11/14	6/14	10/14	10/14	9.2/14
China	6/9	7/9	8/9	6/9	9/9	7.2/9
U.S.	5/11	5/11	7/11	3/11	6/11	5.2/11
Brazil	5/8	6/8	4/8	4/8	4/8	4.6/8
India	6/9	4/9	8/9	8/9	8/9	6.8/9

Table 8. Gamma(5,1): number of missing data points with actual value within 80% posterior interval

Country	Run 1	Run 2	Run 3	Run 4	Run 5	Average
France	11/14	11/14	8/14	12/14	12/14	10.8/14
Italy	14/15	14/15	12/15	15/15	11/15	13.2/15
Germany	12/14	13/14	14/14	14/14	13/14	13.2/14
Spain	6/11	8/11	9/11	7/11	10/11	8/11
Sweden	13/13	13/13	12/13	13/13	11/13	12.4/13
U.K.	12/14	12/14	10/14	12/14	14/14	12/14
China	8/9	8/9	9/9	9/9	9/9	8.6/9
U.S.	8/11	9/11	11/11	7/11	8/11	8.6/11
Brazil	6/8	7/8	5/8	7/8	8/8	6.6/8
India	8/9	6/9	9/9	8/9	8/9	7.8/9

Table 9. Uniform: number of missing data points with actual value within 95% posterior interval

Country	Run 1	Run 2	Run 3	Run 4	Run 5	Average
France	8/14	6/14	6/14	8/14	9/14	7.4/14
Italy	12/15	13/15	11/15	11/15	8/15	11/15
Germany	8/14	10/14	12/14	13/14	8/14	10.2/14
Spain	5/11	4/11	7/11	7/11	8/11	6.2/11
Sweden	12/13	10/13	10/13	11/13	9/13	10.4/13
U.K.	9/14	11/14	6/14	10/14	10/14	9.2/14
China	7/9	7/9	7/9	6/9	9/9	7.2/9
U.S.	5/11	5/11	8/11	3/11	6/11	5.4/11
Brazil	5/8	6/8	4/8	4/8	4/8	4.6/8
India	6/9	4/9	8/9	8/9	8/9	6.8/9

Table 10. Uniform: number of missing data points with actual value within 80% posterior interval
