# Supplementary material for: Simple discrete-time self-exciting models can describe complex dynamic processes: A case study of COVID-19
Source: PLoS One. 2021 Apr 9;16(4):e0250015. doi: 10.1371/journal.pone.0250015 (PMC8034752; doi:10.1371/journal.pone.0250015)
Supplement: S6 Appendix — The histogram represents the estimated posterior distributions for each of the missing data points. The black dashed lines show the 95% credible intervals around the posterior distributions. The solid blue line displays the observed number of deaths. (PDF) [file pone.0250015.s006.pdf]

S6 Appendix: Figures from missing data interpolation

Here we present the histogram of interpolated missing data points for the model where $\mu_1, \mu_2 \sim \text{Gamma}(5, 1)$. Similar results were obtained for all prior choices considered for μ_1, μ_2.

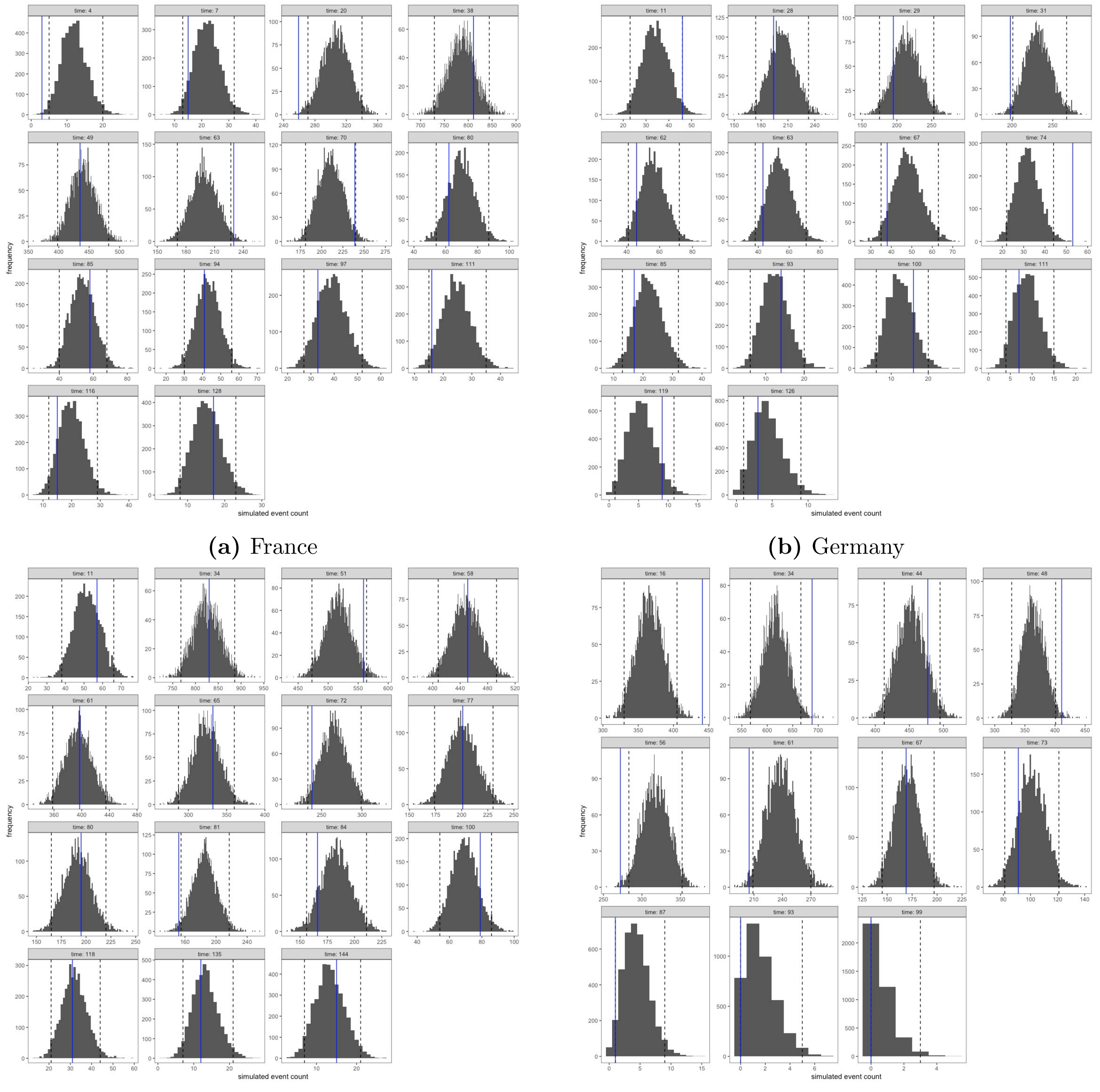

(a) France **(b)** Germany **(c)** Italy **(d)** Spain

Fig 1. Missing data interpolation. The histogram represents the estimated posterior distributions for each of the missing data points. The black dashed lines show the 95% credible intervals around the posterior distributions. The solid blue line displays the observed number of deaths.

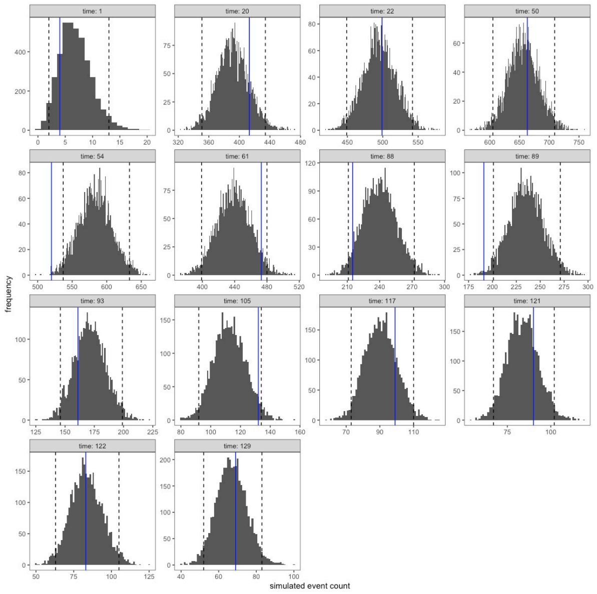

(e) U.K.

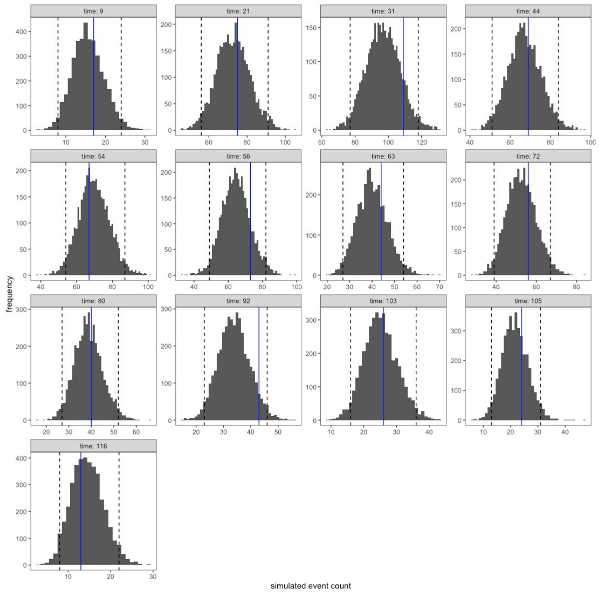

(f) Sweden

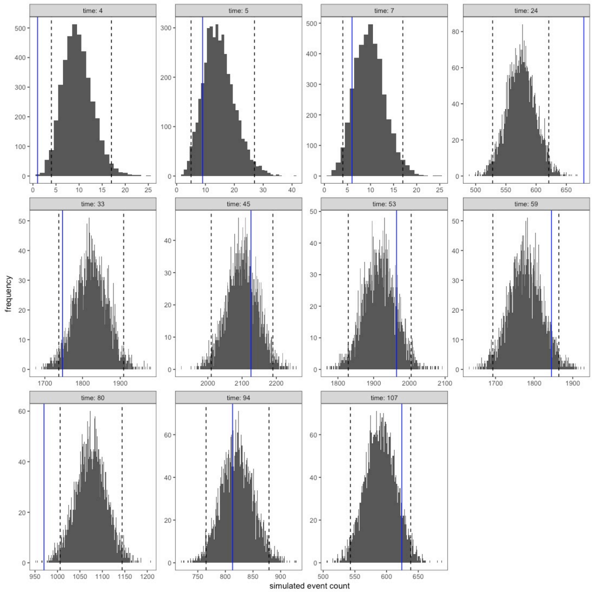

(g) U.S.

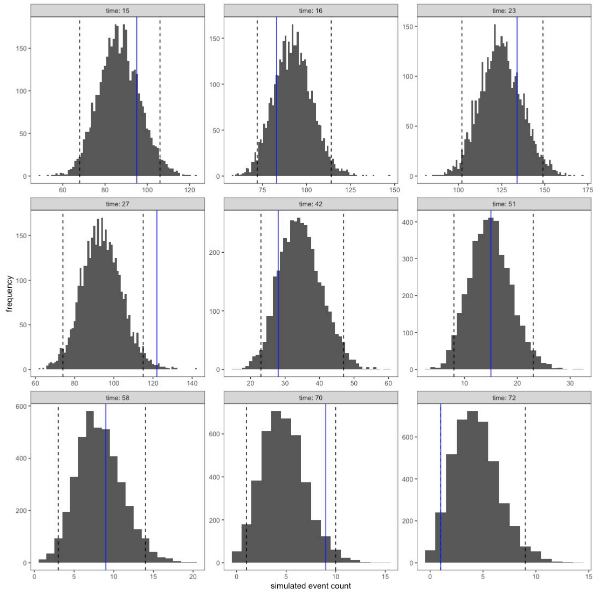

(h) China

Fig 1. (cont.)

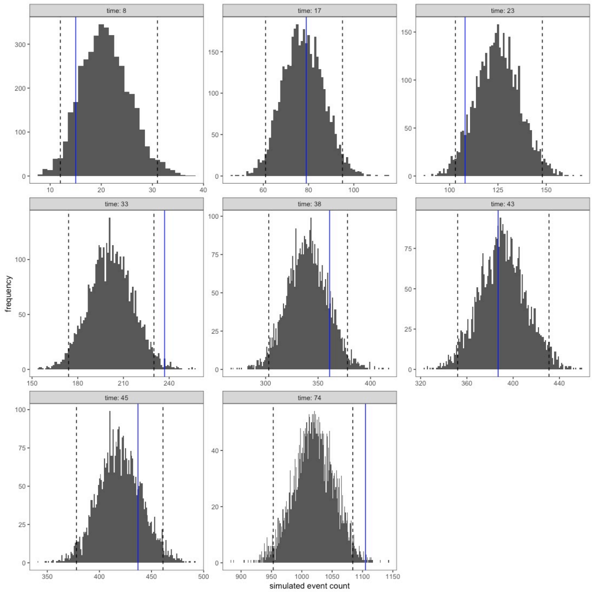

(i) Brazil

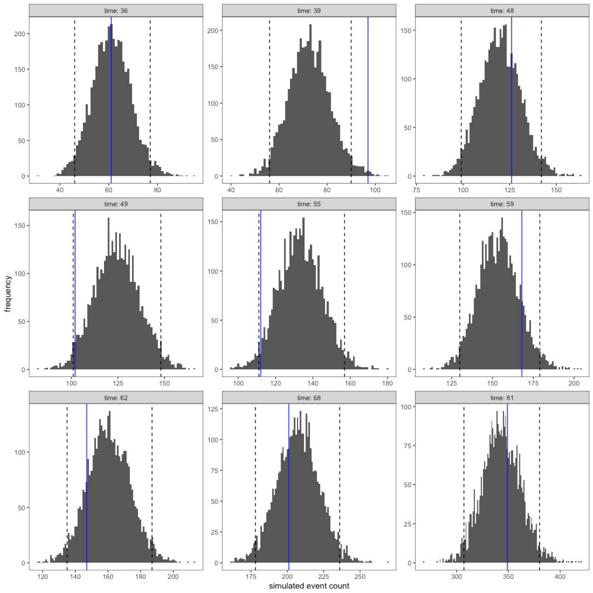

(j) India

Fig 1. (cont.)
