# Supplementary material for: Simple discrete-time self-exciting models can describe complex dynamic processes: A case study of COVID-19
Source: PLoS One. 2021 Apr 9;16(4):e0250015. doi: 10.1371/journal.pone.0250015 (PMC8034752; doi:10.1371/journal.pone.0250015)
Supplement: S7 Appendix — (PDF) [file pone.0250015.s007.pdf]

S7 Appendix: Change point locations

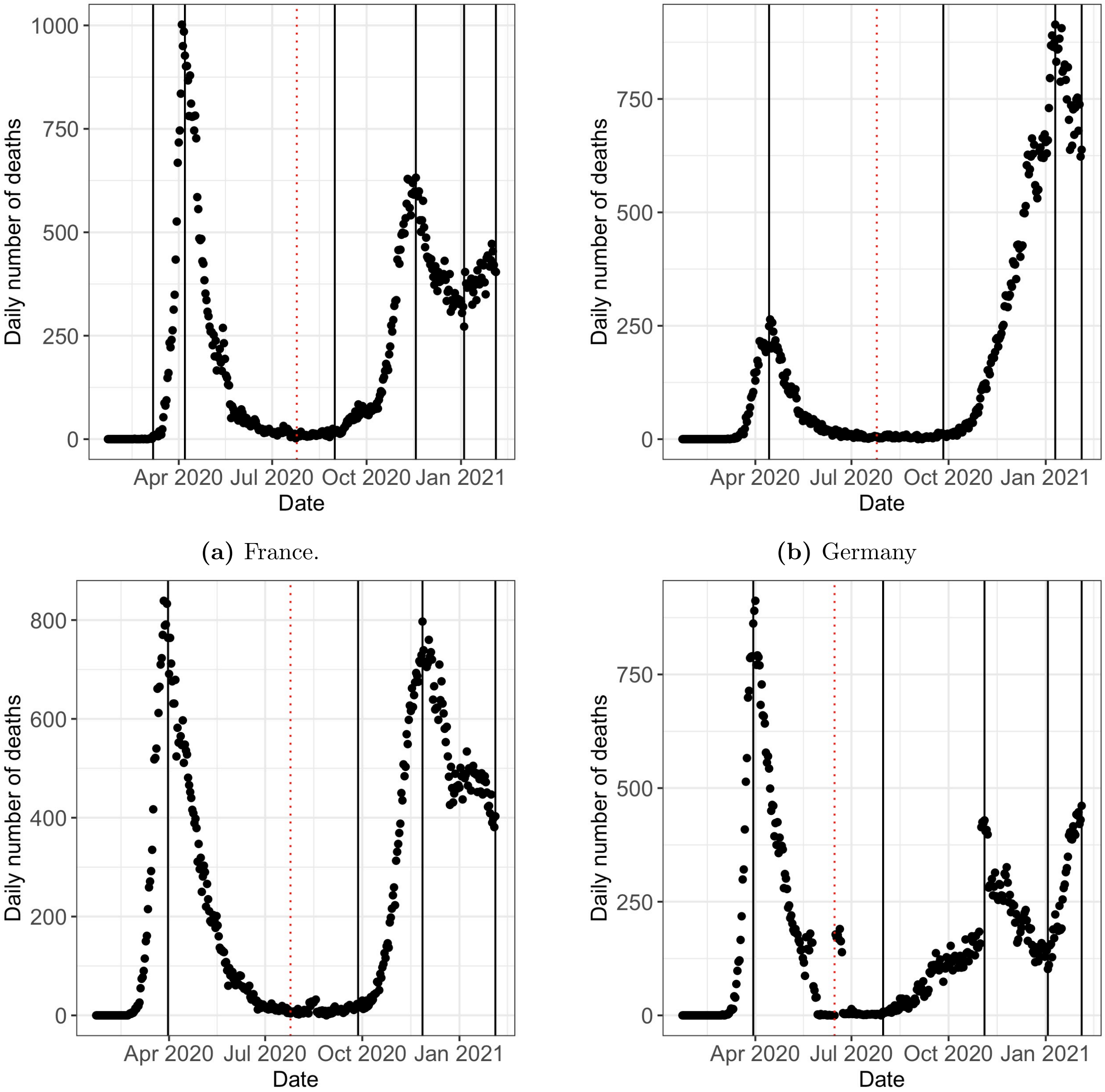

(a) France.

(b) Germany

(c) Italy

(d) Spain

Fig 1. Change point locations. Black vertical lines: change point locations. Black dots: observed data. Red vertical line (dotted): endpoint of initial analysis.

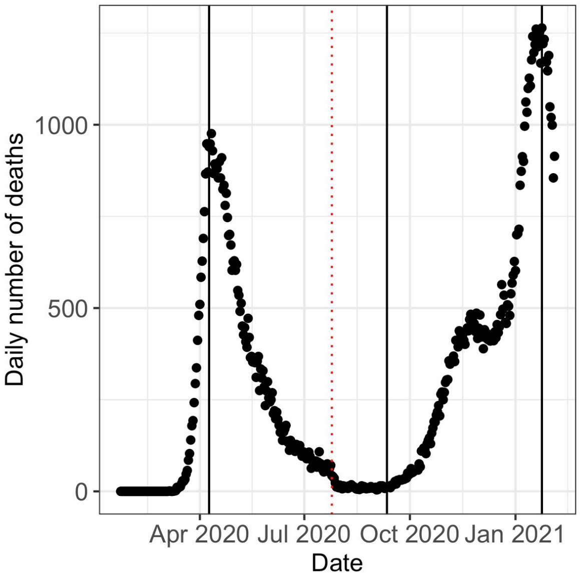

(e) U.K.

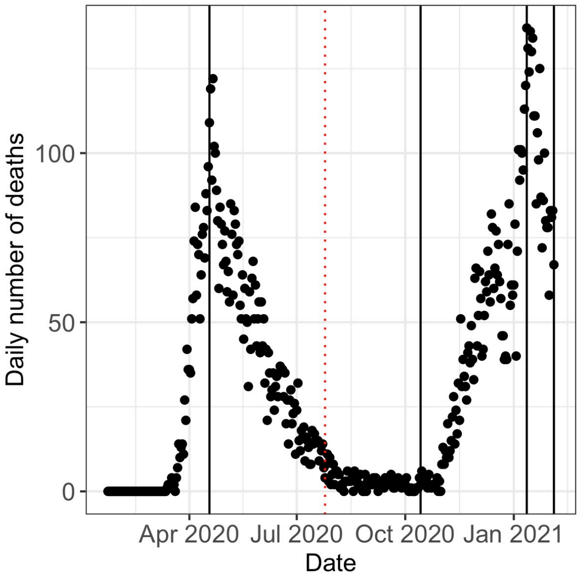

(f) Sweden

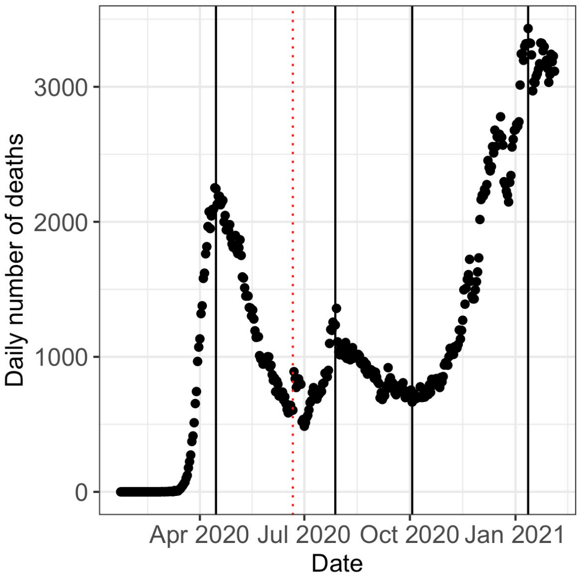

(g) U.S.

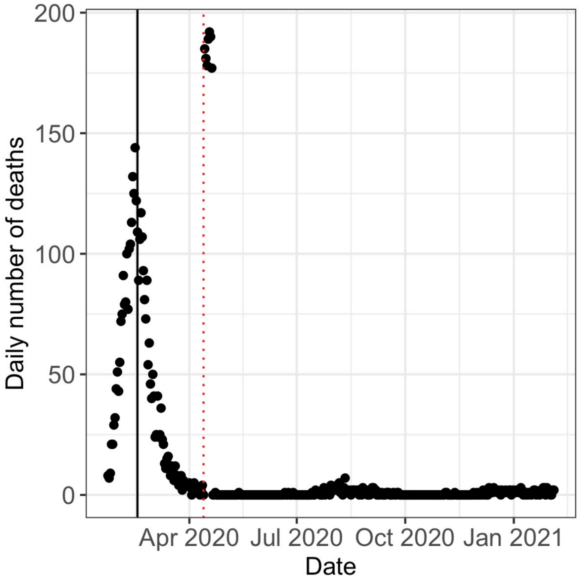

(h) China

Fig 1. (cont.)

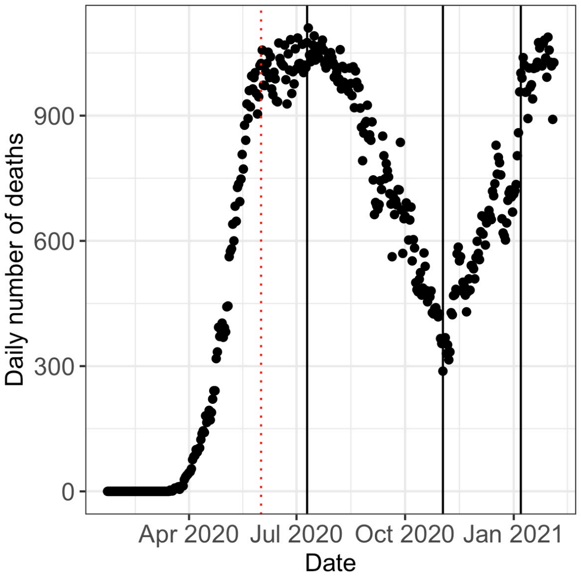

(i) Brazil

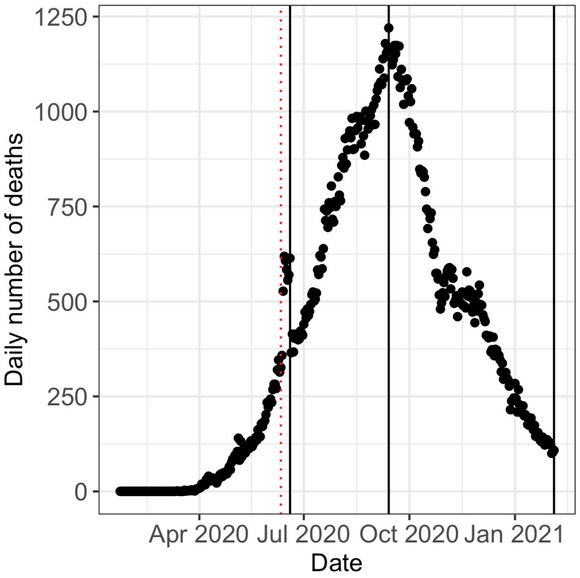

(j) India

Fig 1. (cont.)

Country	Phase	Start Date	End Date
Italy	1	26th Feb 2020	31st Mar 2020
	2	1st Apr 2020	25th July 2020
	3	28th Sep 2020	27th Nov 2020
	4	28th Nov 2020	4th Feb 20201
France	1	7th Mar 2020	7th Apr 2020
	2	8th Apr 2020	25th July 2020
	3	1st Sep 2020	18th Nov 2020
	4	19th Nov 2020	4th Jan 2021
	5	5th Jan 2021	4th Feb 2021
Spain	1	7th Mar 2020	30th Mar 2020
	2	31st Mar 2020	15th June
	3	1st Aug 2020	4th Nov 2020
	4	5th Nov 2020	3rd Jan 2021
	5	4th Jan 2021	4th Feb 2021
Germany	1	13th Mar 2020	14th Apr 2020
	2	15th Apr 2020	25th July 2020
	3	27th Sep 2020	10th Jan 2021
	4	11th Jan 2021	4th Feb 2021
Sweden	1	17th Mar 2020	18th Apr 2020
	2	19th Apr 2020	25th July 2020
	3	15th Oct 2020	12th Jan 2021
	4	13th Jan 2021	4th Feb 2021
UK	1	12th Mar 2020	9th Apr 2020
	2	10th Apr 2020	25th July 2020
	3	12th Sep 2020	24th Jan 2021
US	1	5th Mar 2020	15th Apr 2020
	2	16th Apr 2020	21st June 2020
	3	29th July 2020	3rd Oct 2020
	4	4th Oct 2020	12th Jan 2020
China	1	24th Jan 2020	17th Feb 2020
	2	18th Feb 2020	13th Apr 2020
Brazil	1	3rd Mar 2020	1st June
	2	11th July 2020	2nd Nov 2020
	3	3rd Nov	7th Jan 2021
India	1	23rd Mar 2020	11th June 2020
	2	20th June 2020	13th Sep 2020
	3	14th Sep 2020	4th Feb 2021

Table 1. Dates of phases
