# Supplementary material for: Simple discrete-time self-exciting models can describe complex dynamic processes: A case study of COVID-19
Source: PLoS One. 2021 Apr 9;16(4):e0250015. doi: 10.1371/journal.pone.0250015 (PMC8034752; doi:10.1371/journal.pone.0250015)
Supplement: S8 Appendix — For each country and phase, we calculate the estimated expected intensity of the process (i.e. λ(t)) using the samples of the parameter estimates obtained through the estimation procedure. The histograms then represent the median residual value (median of the difference between the observed number of events and the estimated expected intensity). (PDF) [file pone.0250015.s008.pdf]

S8 Appendix: Plot of residuals

To calculate the residuals, we first determine the difference between the estimated conditional intensity function obtained for each of our posterior samples, and the observed number of deaths. We then take the median residual at each time point to produce the histograms in Fig 1. The residuals are generally centred around zero and higher values are less frequent. Cases where this is not necessarily the case generally arise from phases that have a shorter time series used to estimate the parameters, or where the observed data is not smooth. Additionally, cases where there is a single residual that is significantly larger than the rest are usually either at the peaks or where anomalies are present in the data.

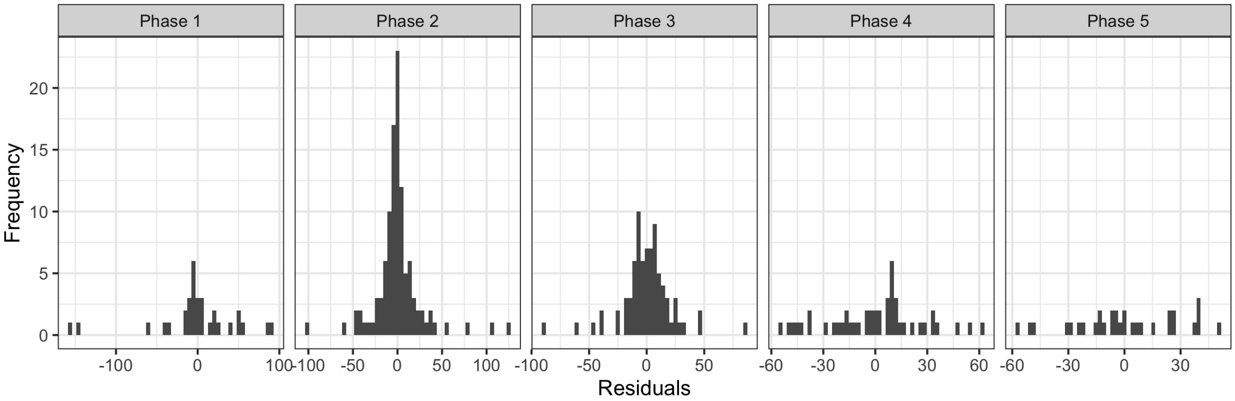

(a) France

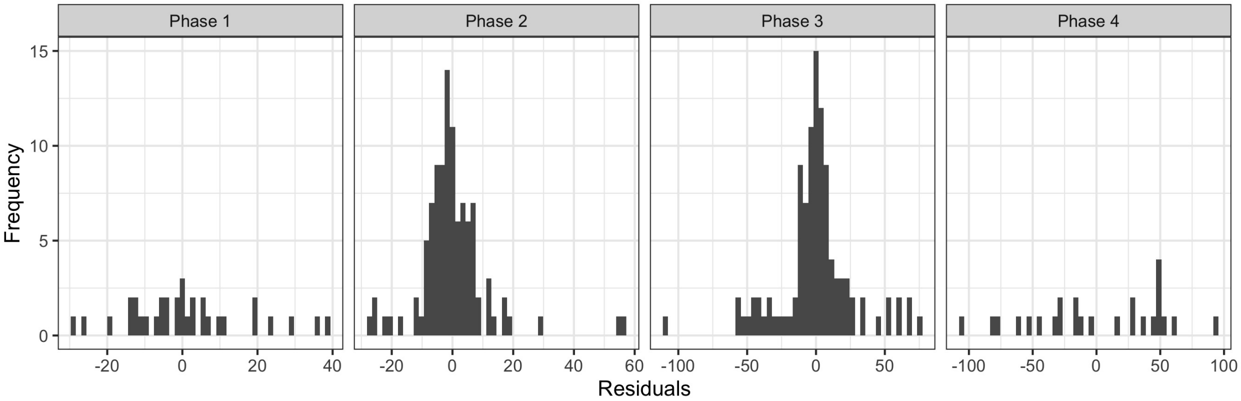

(b) Germany

Fig 1. Plot of residuals For each country and phase, we calculate the estimated expected intensity of the process (i.e. $\lambda(t)$) using the samples of the parameter estimates obtained through the estimation procedure. The histograms then represent the median residual value (median of the difference between the observed number of events and the estimated expected intensity).

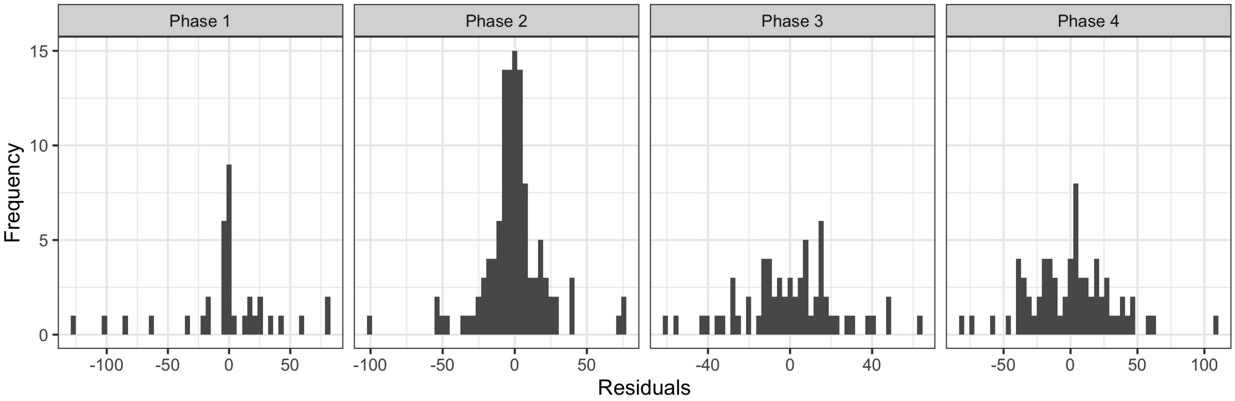

(c) Italy

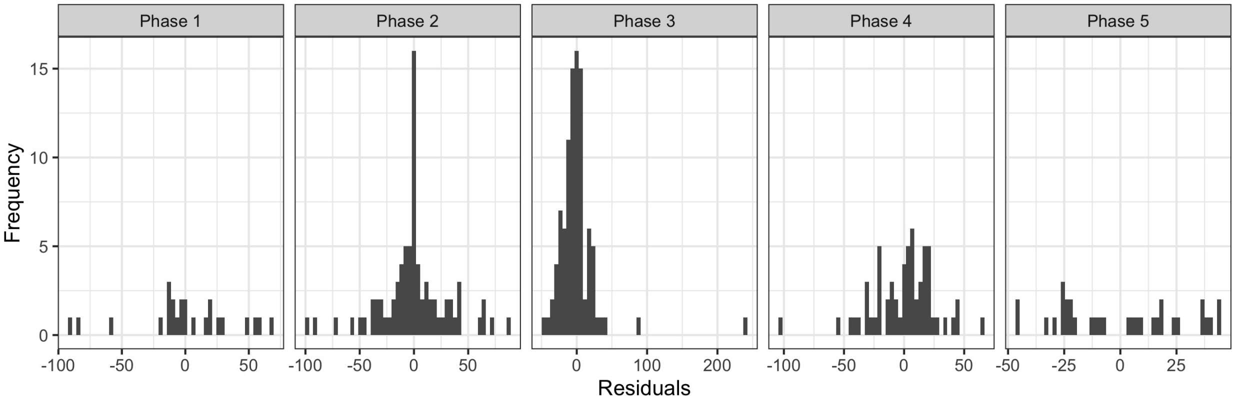

(d) Spain

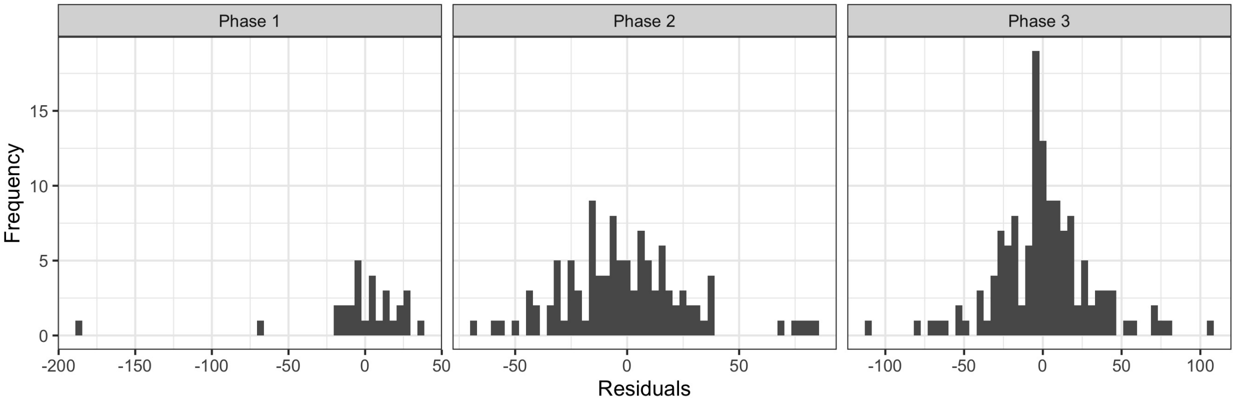

(e) U.K.

Fig 1. (cont.)

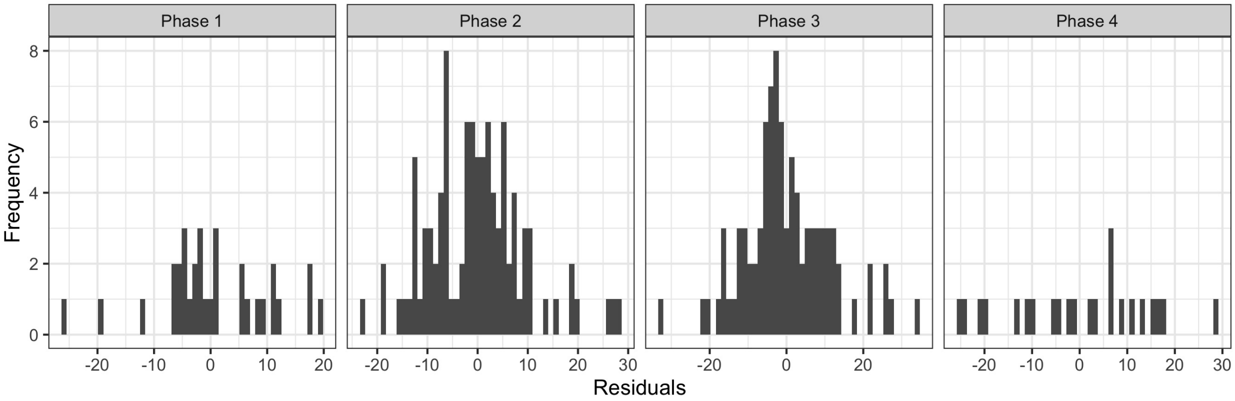

(f) Sweden

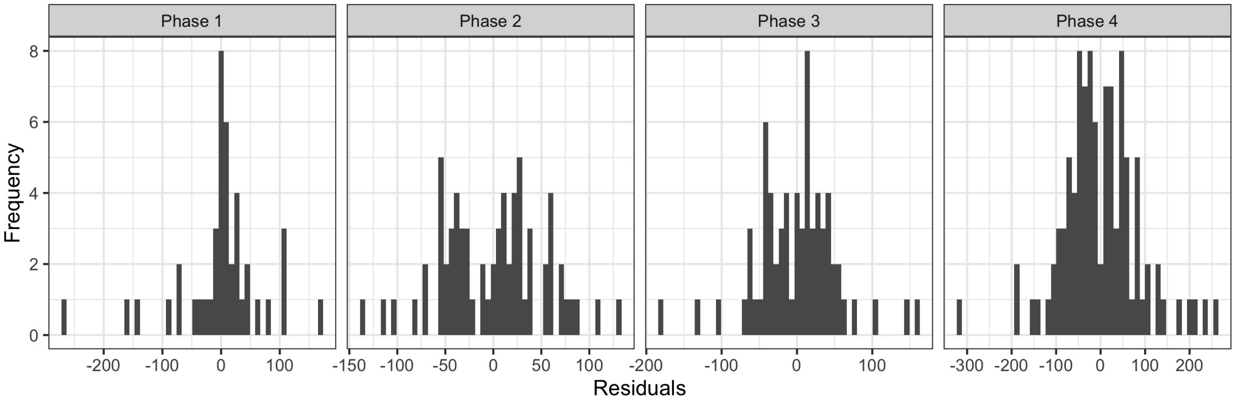

(g) U.S.

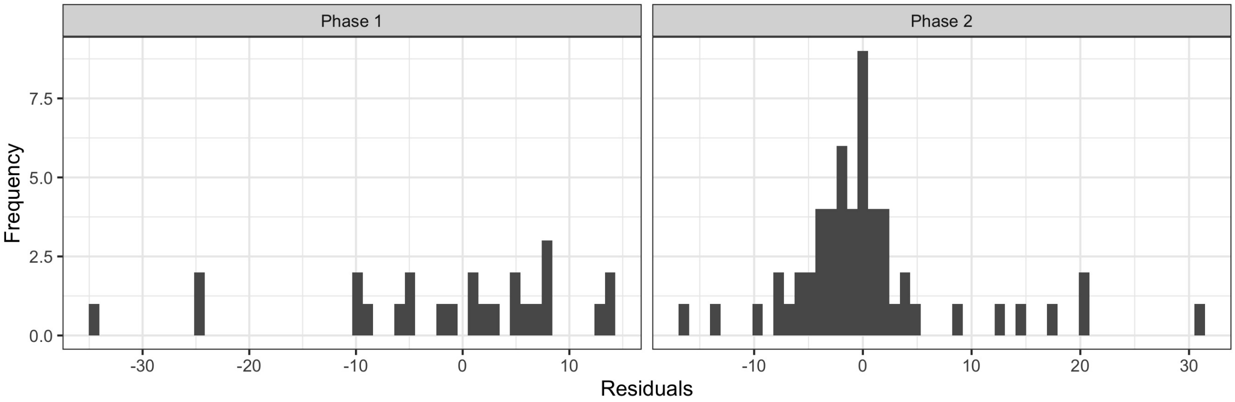

(h) China

Fig 1. (cont.)

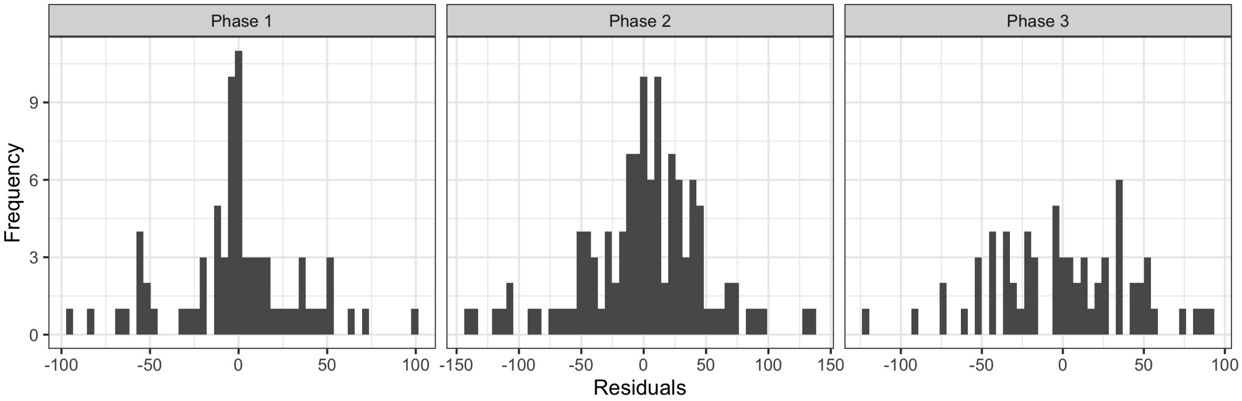

(i) Brazil

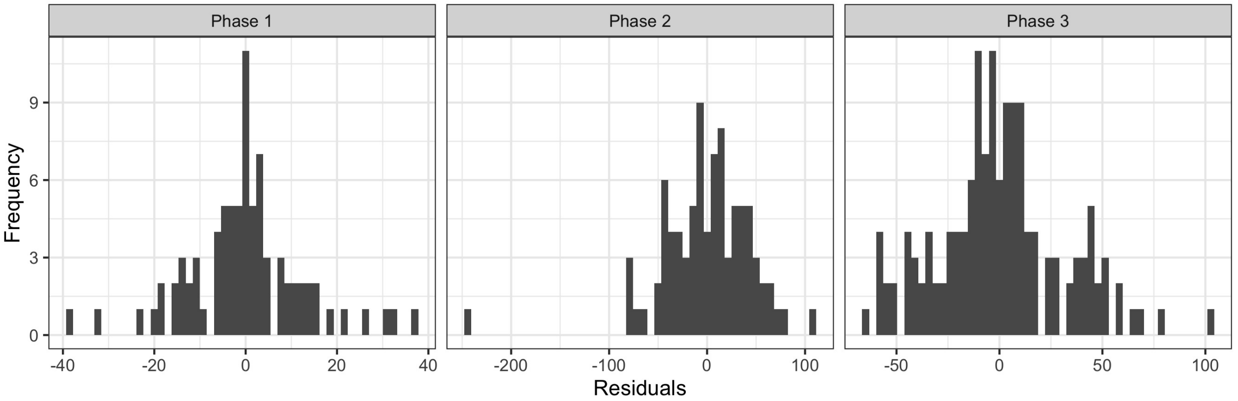

(j) India

Fig 1. (cont.)
