# Supplementary material for: Simple discrete-time self-exciting models can describe complex dynamic processes: A case study of COVID-19
Source: PLoS One. 2021 Apr 9;16(4):e0250015. doi: 10.1371/journal.pone.0250015 (PMC8034752; doi:10.1371/journal.pone.0250015)
Supplement: S1 Table — Expected log predictive density (ELPD) for a range of prior choices. Maximum ELPD in bold. (PDF) [file pone.0250015.s009.pdf]

S1 Table: Results of leave-future-out cross validation with Pareto smoothed importance sampling

Country	$\log N(1,1)$	$\log N(5,1.5)$	Gamma(2,2)	Gamma(5,1)	Uniform
Italy	-2229.5	**-2226.79**	-2392.54	-2240.38	-2242.92
France	-2685.45	-2682.46	-2682.2	**-2680.67**	-2680.93
Germany	-1742.93	-1739.69	-1737.36	**-1734.62**	-1737.57
Spain	**-1827.64**	-1829.85	-1839.56	-1858.82	-1841.94
Sweden	-1548.57	-1547.89	-1546.33	-1545.15	**-1545.14**
U.K.	-2366.67	-2362.07	-2365.69	**-2231.73**	-2365.3
China	-830.56	-829.47	-830.31	-830.56	**-828.78**
U.S.	-2549.46	-2544.98	-2536.99	**-2532.3**	-2545.19
Brazil	-1116.56	-1141.76	**-1108.38**	-1108.98	-1112.18
India	-990.99	-991.39	-990.75	-990.64	**-990.51**

Table 1. Expected log predictive density (ELPD) for a range of prior choices. Maximum ELPD in bold.
