# Supplementary material for: Simple discrete-time self-exciting models can describe complex dynamic processes: A case study of COVID-19
Source: PLoS One. 2021 Apr 9;16(4):e0250015. doi: 10.1371/journal.pone.0250015 (PMC8034752; doi:10.1371/journal.pone.0250015)
Supplement: S2 Table — Comparison of median and 80% intervals of parameters for all phases, using the Gamma(5, 1) prior for μ. (PDF) [file pone.0250015.s010.pdf]

S2 Table: Parameter estimates for original and subsequent analysis

Country	Phase	μ	α	β
Italy	1	4.39 (3.18,5.71)	1.07 (1.05,1.09)	0.88 (0.8,0.95)
	2	1.17 (0.69,1.8)	0.94 (0.93,0.95)	0.55 (0.48,0.63)
	3	3.7 (2.41,5.1)	1.06 (1.05,1.08)	0.59 (0.52,0.66)
	4	5.14 (2.59,8.68)	0.97 (0.96,0.98)	0.52 (0.44,0.61)
France	1	4.57 (3.38,5.91)	1.1 (1.08,1.11)	0.97 (0.92,0.99)
	2	1.57 (0.97,2.28)	0.92 (0.91,0.93)	0.64 (0.58,0.7)
	3	2.58 (1.59,3.75)	1.04 (1.03,1.06)	0.72 (0.62,0.83)
	4	5.24 (2.73,8.82)	0.95 (0.93,0.96)	0.38 (0.29,0.5)
	5	4.71 (2.53,8.17)	1.05 (1.03,1.06)	0.09 (0.07,0.12)
Spain	1	5.78 (4.06,7.6)	1.11 (1.09,1.13)	0.96 (0.9,0.99)
	2	0.49 (0.28,0.76)	0.96 (0.95,0.97)	0.91 (0.85,0.95)
	3	2.08 (1.31,2.98)	1.06 (1.04,1.08)	0.6 (0.55,0.66)
	4	5.64 (3.04,9.1)	0.94 (0.92,0.96)	0.68 (0.59,0.79)
	5	6.46 (3.46,10.68)	1.05 (1.02,1.07)	0.52 (0.42,0.63)
Germany	1	4.17 (2.89,5.54)	1.06 (1.03,1.09)	0.65 (0.57,0.75)
	2	0.95 (0.59,1.39)	0.91 (0.89,0.93)	0.51 (0.45,0.59)
	3	2.39 (1.61,3.25)	1.03 (1.02,1.04)	0.75 (0.66,0.85)
	4	4.63 (2.4,8.02)	0.97 (0.95,0.97)	0.36 (0.29,0.44)
Sweden	1	4.05 (2.88,5.44)	1.07 (1.01,1.13)	0.42 (0.32,0.54)
	2	1.79 (1.05,2.68)	0.92 (0.89,0.95)	0.5 (0.39,0.62)
	3	1.49 (1.02,2.05)	1.05 (1.02,1.07)	0.41 (0.34,0.48)
	4	4.92 (2.61,8.16)	0.91 (0.87,0.94)	0.49 (0.37,0.65)
UK	1	4.51 (3.08,6)	1.14 (1.11,1.17)	0.79 (0.68,0.91)
	2	2.42 (1.32,3.75)	0.95 (0.95,0.96)	0.56 (0.5,0.62)
	3	3.3 (2.25,4.48)	1.03 (1.02,1.03)	0.67 (0.61,0.74)
US	1	4.08 (3.13,5.15)	1.07 (1.06,1.07)	0.99 (0.98,1)
	2	4.1 (2.16,7.12)	0.97 (0.97,0.98)	0.77 (0.66,0.89)
	3	5.28 (2.81,8.8)	0.98 (0.97,0.99)	0.62 (0.53,0.71)
	4	4.85 (2.62,7.93)	1.01 (1.01,1.02)	0.95 (0.9,0.99)
China	1	8.92 (6.29,11.73)	1.07 (1.01,1.15)	0.4 (0.28,0.56)
	2	0.82 (0.48,1.22)	0.8 (0.76,0.84)	0.43 (0.35,0.54)
Brazil	1	4.18 (2.98,5.52)	1.03 (1.02,1.04)	0.83 (0.73,0.93)
	2	3.62 (1.87,5.97)	0.98 (0.98,0.99)	0.62 (0.55,0.69)
	3	5.63 (2.96,9.35)	1.01 (1,1.02)	0.89 (0.8,0.96)
India	1	2.81 (2.02,3.72)	1.1 (1.07,1.13)	0.33 (0.26,0.41)
	2	4.34 (2.31,7.35)	1.01 (1,1.01)	0.64 (0.57,0.71)
	3	2.49 (1.33,4.11)	0.97 (0.97,0.98)	0.58 (0.52,0.65)

Table 1. Comparison of median and 80% intervals of parameters for all phases, using the Gamma$(5, 1)$ prior for μ.
