# Supplementary material for: Simple discrete-time self-exciting models can describe complex dynamic processes: A case study of COVID-19
Source: PLoS One. 2021 Apr 9;16(4):e0250015. doi: 10.1371/journal.pone.0250015 (PMC8034752; doi:10.1371/journal.pone.0250015)
Supplement: Attachment — Submitted filename: Response to Reviewers.pdf [file pone.0250015.s011.pdf]

Response to Reviewers

Simple discrete-time self-exciting models can describe complex dynamic processes: a case study of COVID-19

Raiha Browning, Deborah Sulem, Kerrie Mengersen,
Vincent Rivoirard, Judith Rousseau

Throughout this response document, please note that line numbers refer to the manuscript with tracked changes.

Responses to Reviewer 1

R1.1 Comparison with SIR family models could be more detailed and focused on explaining how these different models are able to model the same phenomenon. One obvious difference, is that SIR considers finite population, while Hawkes process seems to not be aware of population being finite. In case of COVID-19, this might not be important, but to model other more prevalent diseases (e.g., flu), this could play a major role.

We thank the reviewer for their comment. We have now expanded our discussion to detail the key differences between our approach and compartmental models. We first discuss the differences in terms of the population being considered. While the SIR family of models consider the entire infection cycle (i.e. the susceptible, infected, recovered/deceased populations and corresponding transmission and removed rates), our Hawkes model is somewhat simplified as it considers only the deceased population. We have added the following discussion in lines 558-571 of the manuscript.

> "Here we discuss several of the many approaches that have been considered to model the ongoing COVID-19 epidemic, and the different perspectives they provide compared to our DTHP model. Compartmental models such as the SIR family of models are among the most popular methods for epidemic modelling. They are more detailed and consider the mechanics of the infection cycle, separating the population into categories such as susceptible, infected and recovered or deceased. Our DTHP model is simplified in the sense that

we consider only death events. We chose to model deaths instead of infection numbers as the latter data was very unreliable in the beginning due to lack of testing and different testing policies across countries. However, as we show in S1 Appendix, as a first-order approximation, the death dynamics are helpful to understand the infection dynamics. This approximation is convenient when the infection data are unreliable, as occurred in the early stages of the COVID-19 pandemic. In the presence of data uncertainty such as this, the SIR model requires additional terms to account for this measurement error."

We then discuss the assumption of finite population in lines 582-593 of the manuscript.

> "Another significant difference between our model and standard compartmental models is that the latter considers a finite population. In its original form, the Hawkes model assumes that there will be immigrant events arriving at a rate of the baseline mean μ indefinitely, implying an infinite population. However, finite population variants of the Hawkes model do exist (Rizoiu et al., 2018). This differs from the SIR model, which naturally considers a finite population whereby the infection dies out once herd immunity is achieved. The impact of this difference is negligible in our modelling because we predominantly model the pandemic's initial phases, where not enough of the population has been infected or vaccinated to achieve herd immunity. This may not be the case for more prevalent diseases such as the flu, however both models are reasonable. As the flu season ends, there will still be new infections throughout the year, however on a smaller scale."

Rizoiu et al. (2018) is an important paper regarding the finite population assumption. These authors explore the connection between the Hawkes model and SIR models, and develop a finite population variant of the Hawkes model. We have also expanded our discussion on this paper to include a brief comparison of SIR and Hawkes models in lines 64-72 of the manuscript, and also allude to further discussion here.

> "Hawkes processes and compartmental models are based on different mathematical principles and rely on different assumptions. However, their connection was explored by Rizoiu et al. (2018). These authors show that, via a modified, finite population variant of the Hawkes model for a particular choice of triggering kernel, the rate of events is equivalent to the SIR model's infection rate. While the SIR family of models is useful if more is known about the system dynamics, a simpler model is often useful for phenomena where there are many unknowns. We show in this study that our model is helpful for this purpose. Additionally, we explore the differences between Hawkes, compartmental models and other approaches further in the discussion."

Hence we believe a simple model for unknown and volatile phenomena such as the COVID-19 pandemic is a valuable addition to the literature, particularly in the early stages of the outbreak. We discuss the value of our contribution further in lines 594-606, as outlined below.

> "Hence our approach provides a simple model for unknown and volatile phenomena such as the COVID-19 pandemic, particularly in the early stages of the outbreak. Unlike the common flu, where the dynamics and course of infection are well understood and relatively predictable, COVID-19 is a new and unexplored domain. The various interventions that take place simultaneously result in complex interactions that complicate the dynamics of the process. Our focus is on the early stages of the epidemic where there is a great deal of uncertainty and volatility. The SIR model family is useful for phenomena where the mechanics are well known. However, complicated variants of these models are required to capture the complexity of this pandemic. Our simple model is useful in describing this early stage in the pandemic when there are still many unknowns. Our model also introduces randomness and flexibility that is not afforded in standard compartmental models. This allows our model to adapt to system changes induced by government interventions quickly."

R1.2 SIR family models seem to have advantage in that they have natural change point (when effective R becomes smaller than 1). Furthermore around this change point effective R seems to change continuously. In DTHP case, it seems that the change point is exogenously given prior to the fitting procedure. In practice, policy makers might not have this kind of knowledge.

This analysis aims to provide alternative insights into the COVID-19 pandemic, showing how the DTHP can be used to understand COVID-19 and how it can apply to other self-exciting phenomena. As mentioned in the discussion, the reproduction number in epidemiological models has a slightly different interpretation than the magnitude parameters α in our model. While they both represent the extent of secondary infections, the differences in the underlying modelling frameworks mean that they don't have the same properties. The parameters in our model also provide a simplified view of the infectivity of the virus. However other authors (Bertozzi et al., 2020; Mohler et al., 2020) have considered a time-varying magnitude parameter for a Hawkes model, which would have the same effect as considering when the effective R becomes smaller than 1 in the SIR family of models.

For the dataset used in this analysis, it was fairly evident where a reasonable change point was. Thus our simple change point model was easily accommodated for. Since this change point results from policy changes, and although policy makers do not have access to the exact delay of the impact of these measures, the existence of a change and a range

of values for its delay may be known. However, for more complex trajectories, the change point could be estimated, for example using a variant of the method suggested in Li et al. (2017). These authors detect change points in Hawkes process models based on likelihood ratios between the null hypothesis (no change) and alternative hypothesis (change point) reaching a pre-defined threshold. We have also added some more explanation to our change point allocation when introducing the change point in lines 155-162,

> “We do not estimate the change points for our model, as it was fairly obvious where a reasonable change point was in these data, and this avoids complexity arising from different interventions being introduced in each country, with varying levels of restrictions. Furthermore, the delays before tangible results are observed, in addition to the complex and hidden interactions underlying the process, complicate the interpretation of estimated change points. We instead opt for this consistent and simplistic definition of the change point for each country. The change points could however be estimated for more complex trajectories.”

and in the discussion on lines 645-667.

> “The reproduction number, defined as the number of secondary infections from a single case, is a crucial parameter in epidemiological models. Similarly, the magnitude parameters in our model, given by α, also represent the expected number of secondary cases caused by a single parent event. While their respective interpretations are similar at a superficial level, α is not directly comparable to reproduction numbers in epidemiological models. This is due to differences in model assumptions and the underlying mathematical frameworks, as our model's magnitude parameters do not provide the same information as the effective reproduction number. The effective reproduction number informs the level of herd immunity that will bring the virus under control, and the proportion of new infections that must be prevented to change the trend of events from increasing to decreasing (The Royal Society, 2020), whereas our model parameters do not. However, we note that, similarly to reproduction numbers, if $\alpha > 1$ in our model there is exponential growth in the number of events and $\alpha < 1$ leads to a stationary model, which translates into a decrease in the number of deaths if the phase begins at a time with a high event intensity. We also consider a static variable that fundamentally averages over the whole period, rather than varying through time as the effective reproduction number would. We do this as reasonable change points were fairly obvious in the dataset used for this analysis. However, for more complex trajectories, other authors (Bertozzi et al., 2020; Mohler et al., 2020) consider a Hawkes model with a time-varying magnitude parameter, which they refer to as a dimensionless reproduction number. This approach could inform the change point's location

by observing when the magnitude parameter goes below 1. The change points could also be estimated, for example using the method suggested in Li et al. (2017)."

R1.3 I have recently seen another manuscript on Hawkes process [https://arxiv.org/abs/2[illegible]]. It explores different phases of the COVID-19 pandemic, which are not commonly taken into account by SIR family models: phase in which external excitation is present (cases incoming from abroad) and phase in which there is no external excitation. Your modeling seems to indicate that external excitation is negligible (besides first few cases)?

We thank the reviewer for bringing this manuscript to our attention. The external excitation in this manuscript is analogous to our baseline parameter μ. However it differs in that we assume a constant level of external excitation throughout each phase, whereas in this manuscript it is time-varying. Our model also includes this external excitation parameter in the second phase, as well as the initial phase, as exogenous activity is still occurring in the second phase. This is supported in our analysis by comparing the baseline parameter across phases, and observing its reduction in the second phase. However we also note that since we are dealing with deaths, as opposed to the number of confirmed cases, the baseline parameter in our model is not a direct representation of cases arriving from abroad. However as now explained in S1 Appendix, we can link the death model to a similar underlying model for the infections, where α and β have the same meaning but the external parameters would be μ/p where p is the probability of death given infection. Hence roughly our external excitation parameters μ can be interpreted as external arrivals (infections) times p, the probability of death given infection. We have added this discussion to the Related Work section of the manuscript in lines 92-96,

> "Two similar approaches to ours are that of Chen et al. (2020) and Koyama et al. (2021). The former proposes a two-phase contagion model based on an extension of the Hawkes process. This study considers a continuous-time Hawkes process, assume the rate of external events varies through time, and estimate the change point in their model. The authors also assume there is no external excitation after the change point."

and discuss the differences between this method and ours in the Contributions section of the manuscript in lines 130-141,

> "Regarding external events, Chen et al. (2020) also assume there is no external excitation in the second phase of their two-phase model. We make no such assumption, and believe considering external excitation throughout the entire course of the pandemic is a valuable consideration. There are still travellers arriving from abroad, and thus exogenous activity is still occurring in later

phases at a lower rate. This is particularly relevant as many countries have relatively relaxed quarantine requirements, which means that travellers from abroad are still capable of spreading the virus. Although we study mortality data in this analysis, we are able to make a connection between mortalities and infections. In particular, we show in S1 Appendix that the rate of external events in our model can roughly be interpreted as external infections, times the probability of death given infection. This link is particularly useful in the absence of reliable infection data."

Some interesting extensions of our model include introducing a time-dependent baseline process, estimating the change point, or even including covariates related to COVID-19 deaths. These are all interesting directions to pursue in future work, however are out of scope for this analysis. We have also mentioned these extensions to the discussion in lines 545-556.

> "While we somewhat manually select change points in this analysis, an algorithm suitable to this model with automatic selection of the number of change points and their respective locations could also be considered. Additional change points need to be determined carefully as there must be sufficient information in each time series to inform parameter estimation. Another consideration is flexible Bayesian nonparametric splines (DiMatteo et al., 2001) or other methods to provide time-varying parameters. However, the identifiability and existence of this model would need to be established. One could also consider different triggering kernels, including nonparametric kernels in order to improve the flexibility of the model. Another possible extension is considering covariates related to COVID-19 deaths, such as the number of people travelling and number of hospitals per capita."

R1.4 Definition of the excitation kernel uses different notation in definition (lines 190-191) and other equations in the manuscript. In most expressions the excitation kernel is function of time lag only, while in the definition it is a function of two values. Based on further text it is clear that "β" is a model parameter and that "i" represents time lag (which is confusing, because "i" is also used to index excitation events).

We have modified our notation to be consistent throughout the text, in particular we have updated lined 241-242 in the manuscript. The sentence now reads,

> "The triggering kernel $g(t - t_i)$ is selected as a geometric excitation kernel, $g(t - t_i; \beta) = \beta(1 - \beta)^{t - t_i - 1}$."

R1.5 It is unclear what authors mean by "We choose the geometric kernel to resemble the exponential distribution". Why not exponential kernel then?

We use the geometric distribution, as opposed to the exponential because we are dealing with a discrete time resolution, and thus will only ever evaluate the excitation kernel at integer values. Note that $g(t-t_i;\beta) = \beta(1-\beta)^{t-t_i-1}$ decreases exponentially or equivalently it can be written as $\beta/(1-\beta)e^{-(t-t_i)\log(1/(1-\beta))}$ with $1/(1-\beta) > 1$. It is therefore equivalent to the exponential kernel in the context of discrete time. We have made this more clear in lines 244-247.

> "The exponential distribution is one of the most commonly used triggering kernels for continuous-time processes. Thus we choose the geometric kernel as it can be shown to be equivalent to the exponential distribution in the context of discrete time."

Further, we require a discrete-valued probability distribution (that the sum of the excitation kernel over $\mathbb{Z}_+$ be equal to 1) to ensure the interpretation of the magnitude parameter α is sound. We have also made this more clear in lines 194-198.

> "In this study, we specify the triggering kernel to be a proper probability mass function with strictly positive integer-valued support. Since the sum of the excitation kernel over $\mathbb{Z}_+$ is equal to 1, one can interpret the non-negative magnitude parameter $\alpha \in \mathbb{R}_{\geq 0}$ as the expected number of subsequent events produced by a single event (White et al., 2012)."

R1.6 At the definition of the excitation kernel, it is not clear what parameter "β" means, from the later text it becomes clear that it controls average excitation time. Would it be possible to provide an equation for the average excitation time given this kernel?

We are not quite sure what you mean by average excitation time. In our model each event will produce a number of future events at various time points, and at each future time point this number is a random variable (Poisson). Do you mean by excitation time the (random) time after which no new events are induced by a single event? We have now investigated this question and express the expectation of the maximum excitation time in terms of the model parameters, α and β. Roughly speaking its average is of order $c(\alpha)/\beta$ where $c(\alpha)$ is of order α for α that are not too large.

However if you are referring to average excitation in terms of the mean of the geometric kernel, rather than the expected maximum excitation time as discussed in the paragraph above, we have also added a description of β when introducing the model. Additionally,

we have moved the paragraph earlier to clarify what the form of the triggering kernel is, before introducing β.

These additions have been added in lines 247-250 and the detailed working for the expected maximum excitation time can be found in S2 Appendix.

> "The parameter β represents the success probability in the geometric distribution, and thus the average of the excitation kernel is $\frac{1}{\beta}$. We also express the expectation of the maximum excitation time in terms of the parameters of the model in S2 Appendix."

R1.7 Can DTHP be used for forecasting? How well it would perform?

Hawkes processes have been found in some previous studies (Park et al., 2018; Kelly et al., 2019) to be useful for short term forecasting, due to their ability to react quickly to recent activity. However longer term forecasts are not advised, particularly for volatile phenomena such as the COVID-19 pandemic that are not well understood, and there is limited data available. This can also be seen in other studies such as Petropoulos and Makridakis (2020), where even 10 day forecasts had very large uncertainty intervals and often overestimated the actual case numbers. Our out of sample prediction also supports this - these predictions are reliable in the short term but should not be extrapolated out. Ioannidis et al. (2020) emphasise many of the issues and challenges in forecasting epidemics, and the sometimes dangerous consequences. While the stochastic nature of our model allows us to construct measures of uncertainties on predictions, our model is heavily influenced by the most recent event history so long term forecasting is inadvisable, as we previously discussed in the original manuscript. This excerpt can be found on lines 389-397 of the manuscript.

> "Prediction is a difficult task, particularly for complex phenomena such as the COVID-19 pandemic. For this particular model, more recent events have a larger impact on the intensity of the process, and thus the model is very reactive to recent behaviours. Thus prediction performed at a time where abnormal behaviour is occurring will be highly uncertain and often unreliable. Moreover, a prediction is only realistic in the short term and generally only at times where there is no evidence of abnormal behaviour. This is consistent with other models in the literature (Park et al., 2018; Kelly et al., 2019; Worden et al., 2019; Funk et al., 2019; Peixoto et al., 2020). Thus we consider in-sample and out-of-sample posterior predictive checks in this study as a measure of model fit only."

Responses to Reviewer 2

R2.1 Recently, Agent-Based Modeling approaches of Covid19 epidemics revealed non-random nature of the underlying stochastic processes. Based on the fluctuations in social participation and certain biological factors, they lead to the infection spreading, hospitalization, and eventually to the fluctuations of the fatality rate [see Refs.: PLOS ONE 15(10) e0241163 (2020); Computers in Biology and Medicine (2020) 121, 103827; arXiv:2003.10218v1 (2020); Entropy 2020, 22(11), 1236;]

We thank the author for these interesting references. We have now included agent-based modelling approaches of COVID-19 to the introduction in lines 26-27 of the manuscript.

> "More detailed approaches such as agent-based modelling have also been considered by numerous authors (Tadić and Melnik, 2020; Cuevas, 2020; Chang et al., 2020; Burda, 2020)."

We have also taken the liberty of including your description of agent-based modelling in the discussion on lines 618-624.

> "Another more complex approach is that of agent-based modelling. These are more detailed than compartmental models, and are very useful if you have an understanding of the underlying mechanisms. Recent papers using this approach for the COVID-19 epidemic, referenced in the introduction, reveal the non-random nature of the underlying stochastic processes. Based on fluctuations in social participation and certain biological factors, they lead to the infection spreading, hospitalisation, and eventually to fluctuations of the fatality rate."

R2.2 Given a generally considerable delay between the infection day of an individual and eventual fatal outcome, several factors can contribute (both individual and collective) along this timeline. Hence, the Poisson distribution in the context of fatality rate might be a reasonable approximation (still arguments need to be given). Then, the question remains if the same approach applied to the other two time series in the data (that is, the infection rate and recovery rate) would give an adequate description/with potentially different parameters?

The Poisson distribution is not only a very natural choice for modelling discrete phenomena, but it is also a natural approximation of a binomial with large population and small success rate (success = deaths in our case). We have added more detail in lines 204-207.

> "The Poisson distribution is selected as it has an intuitive interpretation regarding the generation of daily death counts on a given day, and because it is a natural approximation of a binomial with a large population and low death rate. More detail is given in S1 Appendix."

In our modelling we consider deaths, as opposed to infections, as infection data was found to be unreliable particularly in the earlier stages of the pandemic. However we now show in S1 Appendix that we can link the model on the death dynamics to a similar model on the infections dynamics, where the interpretation of the parameters α and β remain the same but μ is changed into μ/p, with p the probability of death given infection. Hence, as a first order approximation, our approach can be used to understand the dynamics of the infections as well, which is useful in early stages of a pandemic, when no reliable data on infections are available.

While there is this link between infections and deaths, we would nonetheless expect that the same assumptions would not be reasonable for recovery rates, as these are very high, thus the justification above is not necessarily valid. Additionally, caution with recovery data is also necessary as there is no standard definition of a recovery.

We have added a discussion on this approximation in lines 258-267.

> "Although we consider the deceased population rather than the infected population, there is a connection between the two under some simplifications. Thus studying deaths is useful for understanding the infection dynamics as well. This is advantageous particularly in the early stages of a pandemic, when no reliable data on infections are available. We do not go into the details here, but the key outcome of this is that α, β and a function of μ are interpreted with respect to infections, not deaths. The full derivation is available in S1 Appendix. As this approximation relies on the assumption of a large population and a low death rate, we would not expect this model to be reasonable for other time series where the rate of occurrence is high, such as COVID-19 recoveries."

R2.3 Another question regards applicability of the analysis to the data from the second (and third) wave of the epidemics. In many countries, the developments beyond the data considered in this work are available. They reveal a different course of events, especially regarding the ICU and fatality in this epidemics. It is expected that the parameters and hence the epidemic's path could be different than in the first wave, the question is how different? Furthermore, if they could convey (in)efficiency of the social measures that we are currently experiencing, e.g., in Europe?

We thank the reviewer for their comment, and agree that this would be an interesting and revealing analysis. Including the second and third waves involves incorporating additional

change points into the model and producing separate parameter estimates for these phases. Comparison of the model parameters throughout phases can then indicate the differences in severity throughout the various waves.

We now consider the available data up to 4th February 2021. We have alluded to this additional analysis in lines 251-256,

> "It is straightforward to extend Eq (2) to allow for additional change points. While the majority of this paper considers only the initial phases of the pandemic up to 25th July 2020, we consider subsequent phases after this date as a set of additional analysis. This is to demonstrate how our model can be extended beyond the initial phases of the pandemic, as new data will continue to become available each day for the foreseeable future."

and have added an additional section title "Fitting subsequent phases" in lines 442-483 to discuss these new results. Note that figure numbers in the quote below refer to the manuscript, not this response document.

> "As the pandemic progresses further waves of infection, and thus deaths, are inevitable and will continue to be of interest for the foreseeable future, particularly as a vaccine is rolled out and new variants of the virus are discovered. There is no obvious endpoint to the pandemic, however it is of interest to investigate subsequent waves of infection as well. To address this, we extend our main analysis to determine whether our proposed model is applicable over a longer time period.
>
> We consider mortality data from the endpoint of our initial analysis, up to 4th February 2021. Countries with inadequate data to inform another phase were cut short. As such, the observation period for Brazil, U.K and U.S end on 7th January 2021, 24th January 2021 and 12th January 2021 respectively. Furthermore, for many countries there is a period of very low mortality in between the first and second waves of infection, and we do not consider this period. Additionally, China has not experienced a second wave, and thus it is excluded from this subsequent analysis.
>
> Change points were selected where there were obvious changes in the trajectory, in a similar fashion as the main analysis. The starting point of the second wave was selected as the time where either the 2 week or 4 week rolling average increases by 50% in a single week. The choice between a 2 or 4 week rolling average is chosen based on which more closely aligns to the start of the second wave upon visual inspection. We note that automatic change point detection algorithms such as the CUSUM algorithm (Killick and Eckley, 2014) were considered, however, they are not appropriate for our model. These algorithms are generally based on the mean of the time series. Given the self-exciting nature

of our model, changes in the intensity of the process do not necessarily indicate changes in the underlying model parameters. The change points selected can be found in S7 Appendix.

Comparing the parameter estimates between the initial analysis and this subsequent analysis, several observations can be made. The full table of estimates can be found in S2 Table. Generally, while the baseline parameter μ in the initial analysis shows a reduction between the first and second phases, in subsequent phases the baseline mean begins to increase again. This is potentially due to the relaxing of restrictions and the opening of international borders. The magnitude parameter α acts as expected, in other words it is less than 1 for phases with a downward trajectory and greater than 1 for phases with an upward trajectory. In the initial analysis, β is generally close to 1 in the first phase and reduces in subsequent phases.

Fig 6 shows the estimated intensity function against the observed data for the subsequent analysis. We find that the estimated intensity follows very closely to the observed data, as is also seen in the main analysis. We also consider in-sample (Fig 7) and out-of-sample validation (Fig 8), in the same manner as the main analysis. These both show promising results, with both in-sample and out-of-sample predictions aligning very closely to the observed data. The residuals, in this case referring to the difference between the observed data and the estimated intensity, for all phases in both the initial and subsequent analysis are provided in S8 Appendix, and support that the models for both sets of analyses are reasonable. ”

The inefficiency of social measures would be an interesting research question but requires a broader investigation to determine. Comparing the parameter estimates between phases would give some indications, but it would not be a definitive conclusion, and is even further complicated when considering other factors such as vaccines.

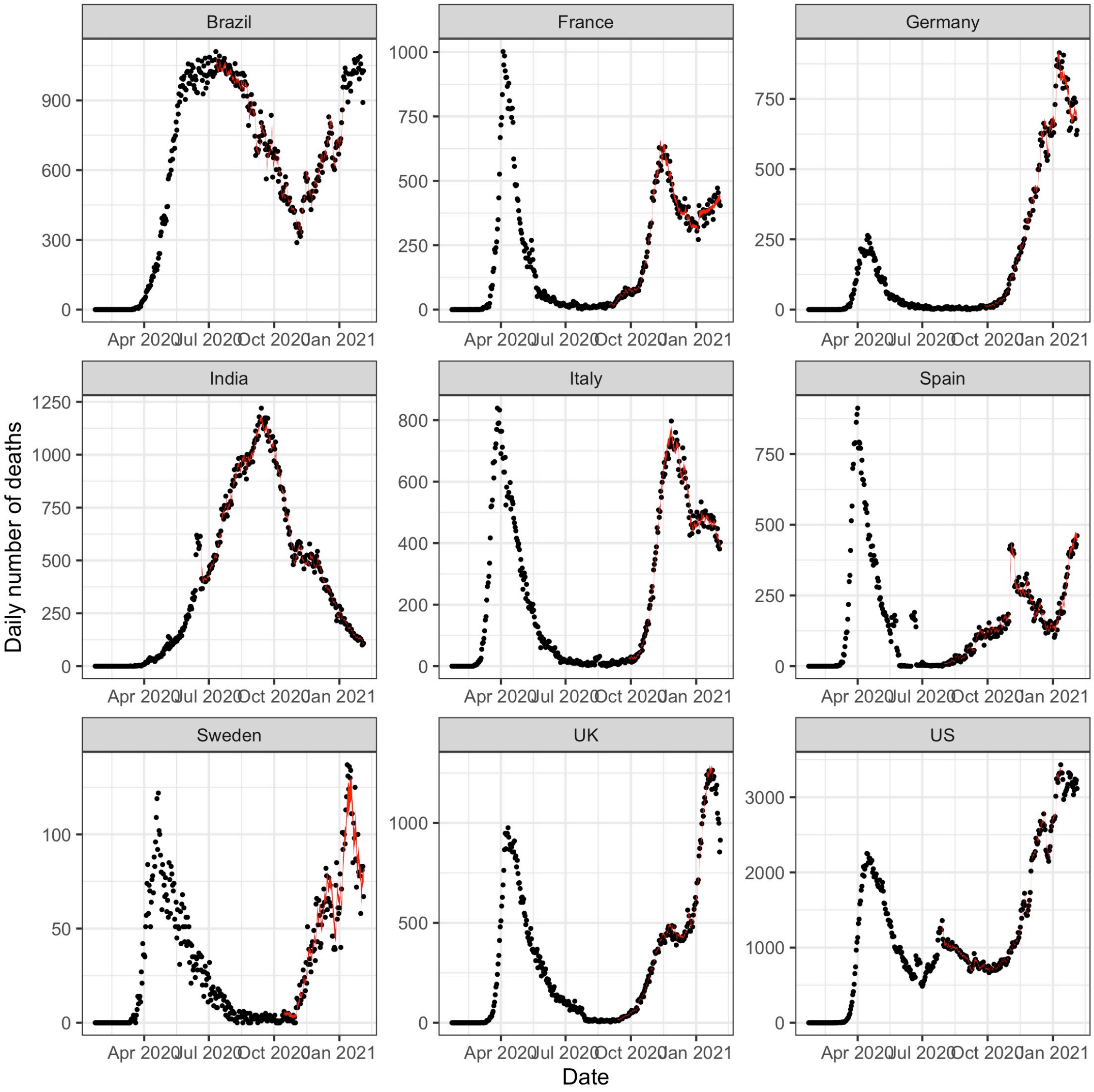

Figure 1: **Observed deaths versus estimated deaths (subsequent analysis).** The observed number of deaths (black dots) compared to the 95% posterior interval for the estimated expected number of events, i.e. $\lambda(t)$ (solid red ribbon), for the subsequent analysis.

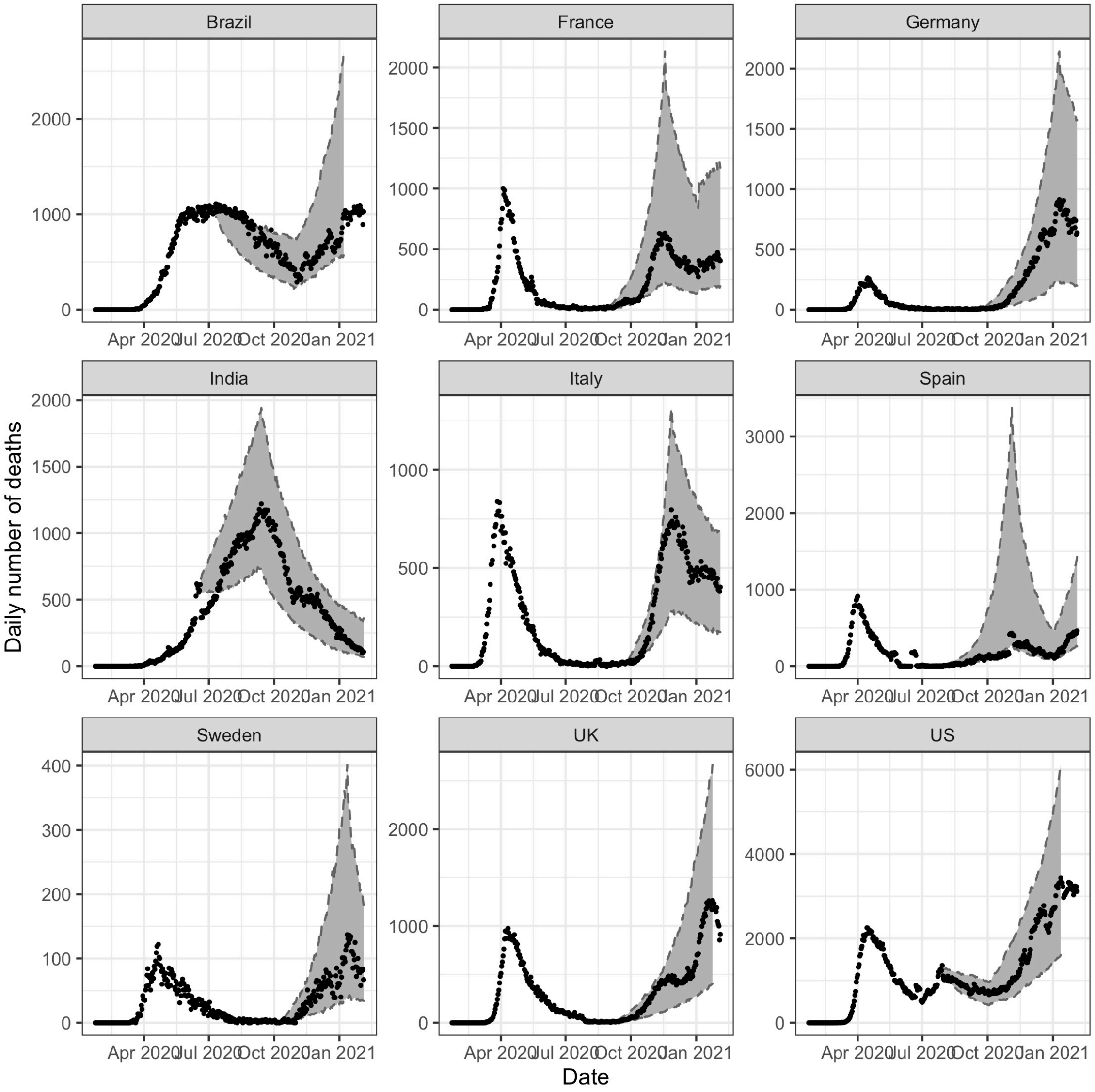

Figure 2: **In-sample validation for subsequent analysis, conditioned on data from the initial analysis.** The observed number of deaths (black dots) compared to the 95% posterior predictive interval for the estimated expected number of events , i.e. $\lambda(t)$ (grey ribbon), for the subsequent analysis.

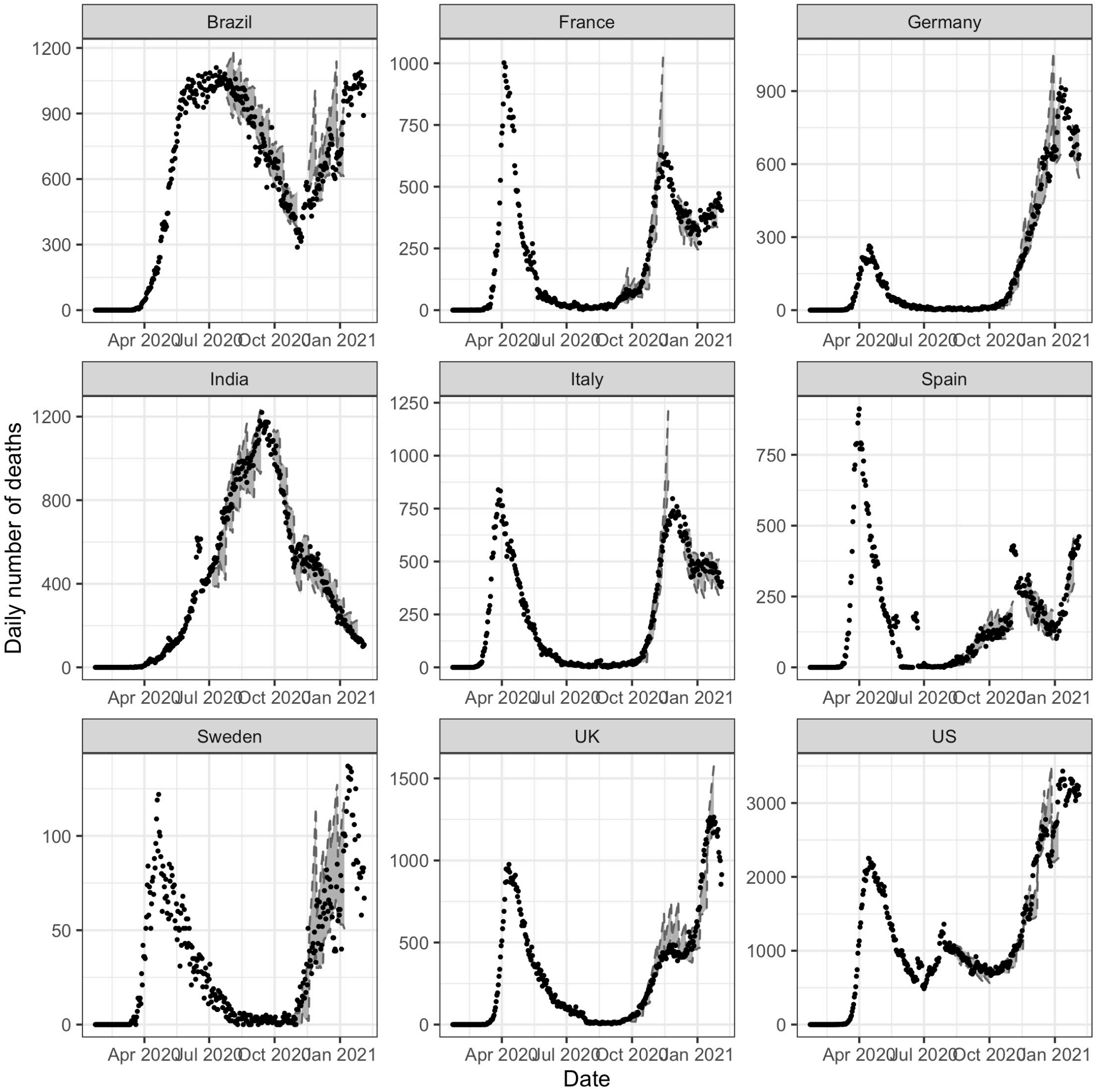

Figure 3: **Out-of-sample validation (subsequent analysis).** The observed number of deaths (black dots) compared to the 95% posterior predictive interval for the estimated expected number of events , i.e. $\lambda(t)$ using various training datasets (grey ribbons) for the subsequent analysis.

Responses to Reviewer 3

R3.1 The number of infected individuals and deaths during an epidemic are known to proceed with the basic pattern shown in Figure 1, 2, etc. This shape has been well-modeled using the SIR model and its variants for many previous epidemics (e.g., Lipsitch et al. 2003). In the SIR model, this shape (a rise to a peak and then fall) results from the reduction of susceptible (S) individuals over time that is, there are fewer deaths later in the epidemic, because there are fewer people left who have not yet been infected or have already died. Being a stochastic model, the Hawkes process doesnt capture these longer time-scale underlying dynamics and the authors are forced to introduce a change point to effectively model the pre-peak and post-peak trajectories. While reading this paper, I was left wondering which is the appropriate modeling approach and does the Hawkes process model leave out important time-scales in the system? Can the authors comment more on how the Hawkes process model structure is linked to their presumptions about the underlying processes driving the death dynamics? Lipsitch, M., Cohen, T., Cooper, B., Robins, J. M., Ma, S., James, L., ... & Fisman, D. (2003). Transmission dynamics and control of severe acute respiratory syndrome. Science, 300(5627), 1966-1970.

We thank the reviewer for their comment. There are many reasons why death dynamics can change. There is considerable uncertainty in the deaths process due to complex interactions. An example is advancements in medical treatments for COVID-19 co-occurring with medical facilities becoming overwhelmed and unable to take more patients. Thus, we make no explicit assumptions about the underlying processes driving the death dynamics. Instead, we assume deaths are driven by infections, as we discuss in S1 Appendix. We have added more discussion in lines 222-234 of the manuscript.

> "In the initial period of analysis, to accommodate this shape, we assume in our analysis that two phases can adequately separate the underlying dynamics. Namely, these phases are the initial period where the virus is spreading rapidly and the following period of reduced contagion resulting from the introduction of preventative measures and policies. Many complex interactions are occurring in the deaths process. For example, as medical professionals become more familiar with the virus and treatments are improved, medical facilities are better equipped to deal with COVID-19 patients in critical condition requiring ICU (Dennis et al., 2021; Horwitz et al., 2020). However, this can be offset by increased demand for hospital beds, resulting in medical facilities becoming overwhelmed and unable to care for all patients that require hospital treatment.

> Therefore, rather than making explicit assumptions about the underlying processes driving the death dynamics, we link our Hawkes model on the death dynamics to a similar infection model, as we discuss in S1 Appendix."

SIR models typically have the shape of an increase followed by a decrease due to a significant reduction in the susceptible population. In the early stages of the pandemic, which we cover in our analysis, the observed decline is not related to a decrease in the susceptible population, but rather by external factors. We now discuss this in the manuscript in lines 607-617.

> "The family of SIR models naturally follow the pattern of infections and deaths rising to a peak and then falling due to a reduction in the susceptible population. However, this is not the cause of the fall observed in the early stage of the pandemic. Instead, the fall is driven by external factors such as social distancing measures, temperature, and improvement in treatments, to name a few. SIR family models have also incorporated change points or time-varying parameters to account for these alternative drivers (Piccolomini and Zama, 2020; Mbuvha and Marwala, 2020). Given our analysis's retrospective nature, the change points were quite obvious, and we did not estimate them. However, our Hawkes model can be easily augmented to induce this shape naturally. For example, we could consider a mixture of Hawkes processes for each of these distinct phases, estimate the unknown (or known) change points, or incorporate time-varying parameters."

While standard compartmental models are indeed crucial to modelling infectious diseases such as the COVID-19 pandemic, we believe that there is no single appropriate model to use. Different approaches provide unique perspectives into the behaviour of the system. Our model aims to provide additional insights, alongside these models, for example, an indication of the change in external excitation through the baseline parameters μ, and the time scale of infectivity through the triggering kernel. Our model also introduces randomness and flexibility that is not afforded in standard compartmental models, allowing our model to easily adapt to system changes induced by government interventions.

R3.2 Similarly, how does the Hawkes process model compare to simply a piece-wise exponential model (i.e., rate linear in x) with a change-point at the peak from a positive growth rate before the peak to a negative growth rate after the peak? If the peak is estimated from the data, this model would have 2 additional degrees of freedom. Are the small-scale fluctuations predicted by the Hawkes model worth the additional parameters?

We thank the reviewer for this interesting question. The Hawkes model is a natural model for self-exciting phenomena and provides a flexible and stochastic framework for modelling. The additional parameters can also provide more nuanced insights into the pandemic. We go into more detail in the excerpt below. On the other hand, the exponential model tells us only about the exponential model's growth rate and the starting value. These are uncertain times, and our model strikes a balance between modelling the dynamics of the whole infection cycle and fitting a generic exponential model. We discuss the value of these additional parameters in lines 636-644.

> "While there are many alternative approaches available, the Hawkes model is also a natural model for describing self-exciting phenomena. It provides a flexible and stochastic framework for modelling, and the parameters in our model provide interesting insights into the pandemic. Namely, α is the average number of secondary infections and is related to the reproduction number, $\frac{\alpha}{\beta}$ is related to the average time an infected individual has infected someone, and μ relates to the occurrence of external excitations, or rather contaminations weighted by the probability of death given contamination. The β parameter on its own also indicates how the time between infections changes throughout time."

We believe that allowing for these fluctuations is essential in understanding the physical system dynamics, and provides a more realistic model. The Hawkes process allows for uncertainty in the model that is not possible with the exponential growth model, which is very strict and captures only the data trend. We show this in Fig 4, which shows that the exponential model fails to fit the data's tails for the first phase. Table 1 also indicates that our model gives a much better fit in both phases. Furthermore, it would be more prudent to undertake a model comparison with less strict models, such as Weibull or Gamma models where exponential is a special case. Based on these results, once could determine whether an exponential growth model is appropriate.

Our self-exciting model relaxes some of the constraints that come with the exponential model and provides a more flexible modelling approach. The exponential model is sensitive to the initial number of cases, possibly producing inaccurate results if there are changes in behaviour as time goes on. For example, if countries become more prepared after the first few weeks or increase testing/healthcare capacity. We observed this inadequacy in

Kasilingam et al. (2020). However, this is not as much of an issue for the Hawkes model. The effect of previous events diminishes as time goes on, and thus, more recent events are more influential. The exponential model may also be less appropriate for the downward trajectory (Tovissodé et al., 2020) and later phases of the epidemic. This is because, during this time, complex interactions between infection and preventative measures result in volatile behaviour and unpredictable trajectories (i.e. not exponential).

Predictions using the exponential model are also very sensitive to the parameter values. The exponential often fails to fit the tails of the data. This is likely where we would predict the data out from, which could result in poor predictions. Additionally, the predictions will be even worse if there are deviations from the trend of the data. This is an advantage of our model. When the contagion deviates from the overall trend, possibly indicating another outbreak, our model can show the expected trajectory in the short term if authorities took no action. This could highlight the importance of reacting quickly with preventative measures. As we demonstrate in our out-of-sample analysis (Fig. 6), short term predictions provided by our Hawkes model are quite good, primarily due to the reactivity of the process itself. This contrasts with the exponential model, which often severely over-predicts the tails in the first phase (Fig. 5).

We summarise these points lines 625-635.

> "Alternatively, one could consider an even more straightforward approach, such as a piece-wise exponential model. However, the Hawkes process allows for uncertainty in the model that is not possible with the exponential growth model, which is very strict and captures only the data trend. Allowing fluctuations in the data - particularly for volatile phenomena such as the current pandemic - is an essential aspect of providing a realistic model. The exponential model also becomes less appropriate as the pandemic progresses. In later phases, there are complex interactions that result in trajectories that are inherently not exponential. These are uncertain times, and our model strikes a balance between modelling the dynamics of the whole infection cycle and fitting a generic exponential model. Thus, we model some fluctuations motivated by the physical process, but with a simpler model than many others considered in the literature."

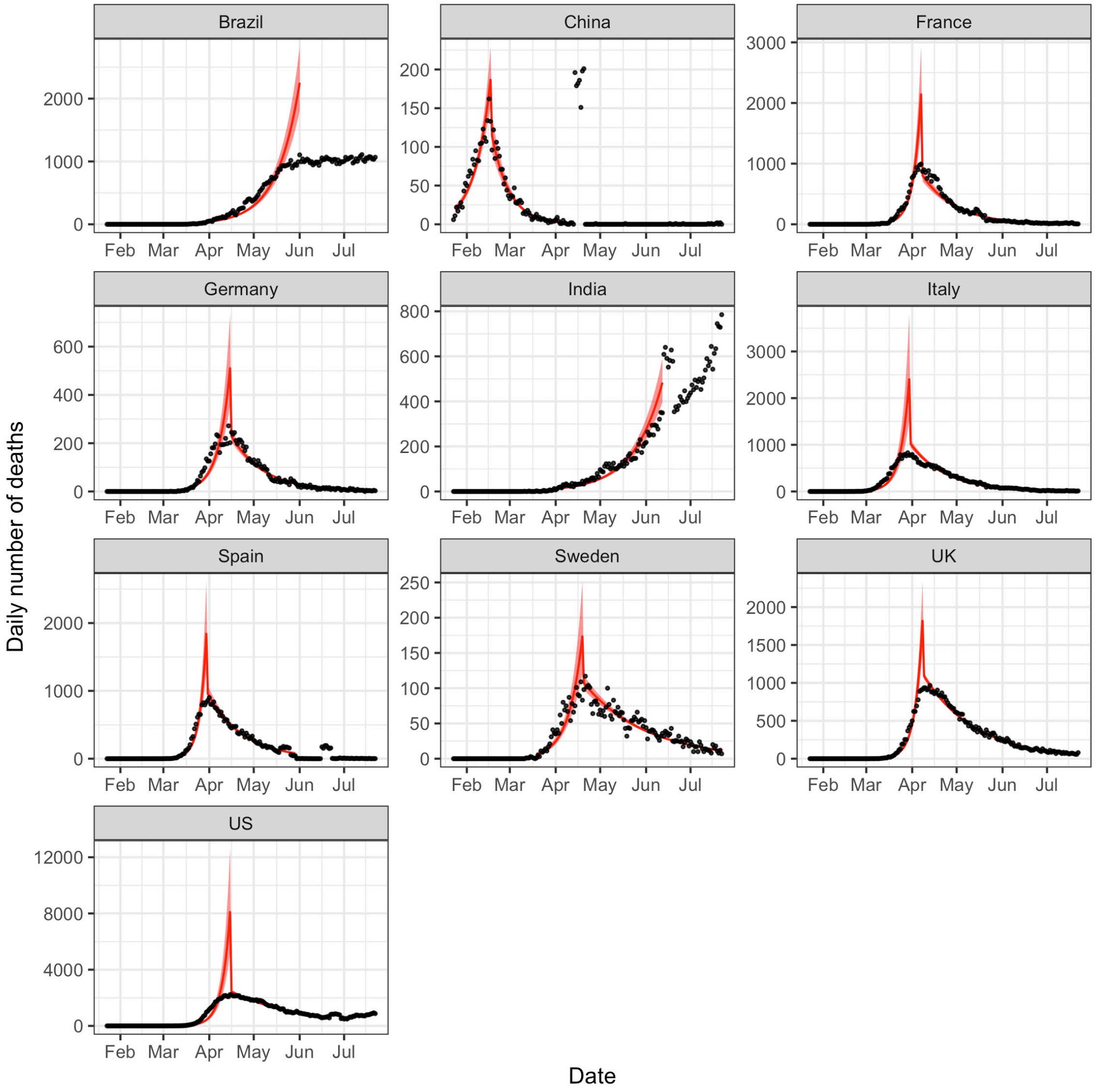

Figure 4: 2-phase exponential model. Black dots: observed data. Red line and ribbon: estimated exponential growth curve and 95% confidence interval respectively.

Table 1: Root mean squared error (RMSE) for exponential and DTHP 2-phase models

	Exponential		DTHP	
	RMSE (Phase 1)	RMSE (Phase 2)	RMSE (Phase 1)	RMSE (Phase 2)
France	274.99	310.92	38.30	29.25
Italy	408.13	298.06	40.66	17.95
Spain	267.07	422.24	44.33	32.67
Germany	85.31	86.65	24.06	10.22
UK	220.20	445.85	35.59	28.06
Sweden	25.53	47.41	11.17	8.89
China	17.18	39.92	10.99	7.00
India	41.05	-	14.75	-
Brazil	303.70	-	30.89	-
US	1430.20	1400.14	67.18	51.30

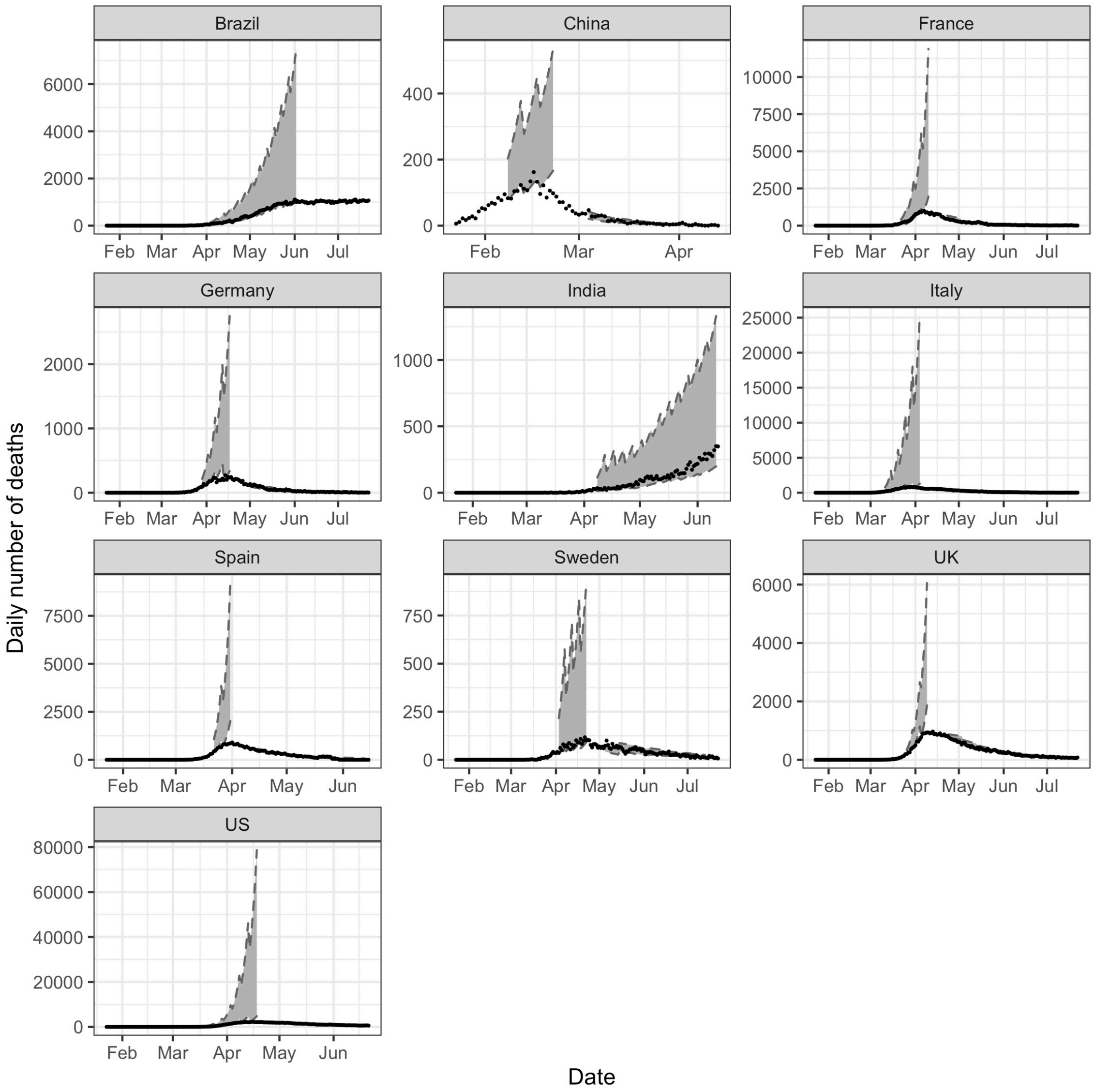

Figure 5: 2-phase exponential model: out-of-sample predictions. Black dots: observed data. Grey ribbon: 95% prediction intervals.

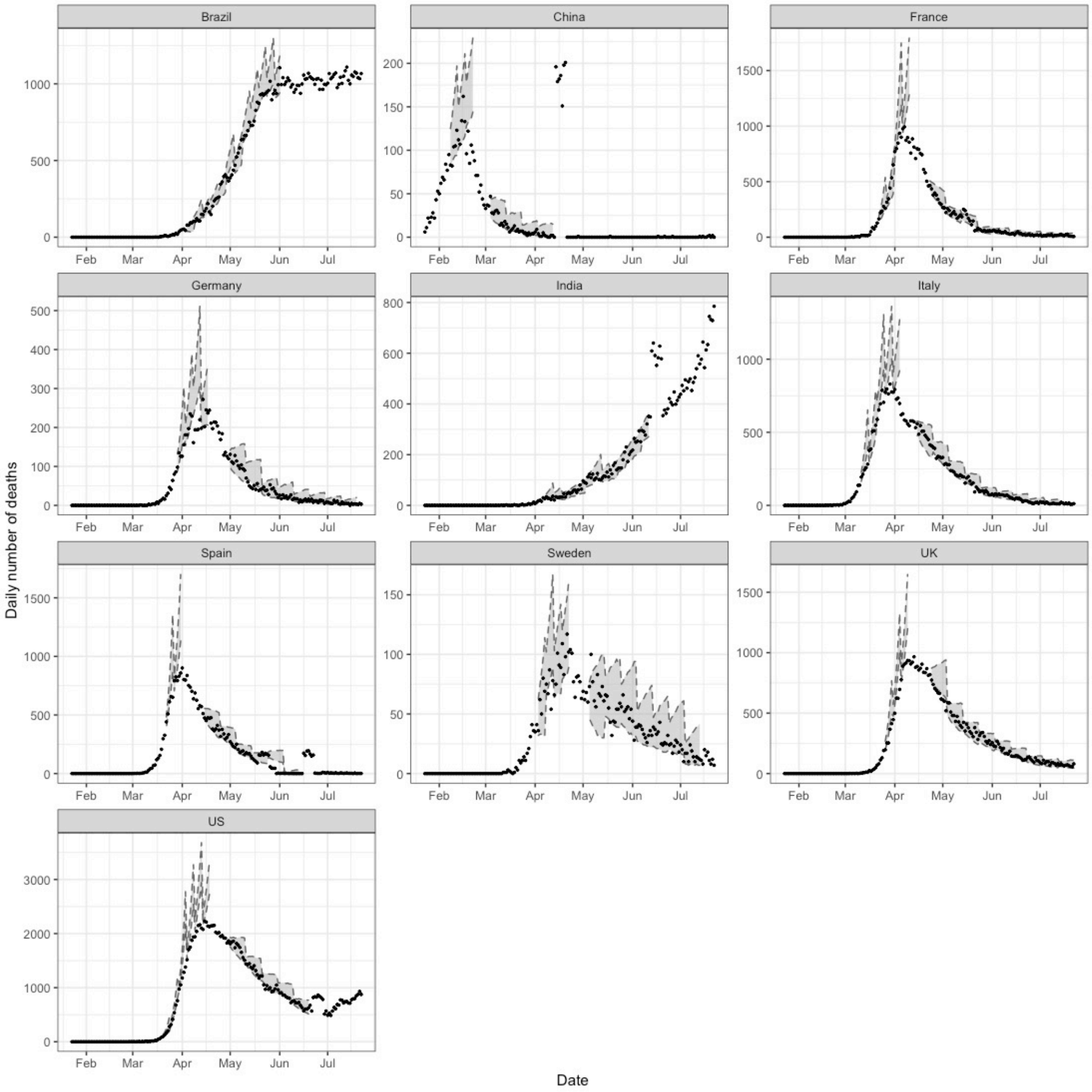

Figure 6: 2-phase DTHP model: out-of-sample predictions. Black dots: observed data. Grey ribbon: 95% posterior prediction intervals.

Additional Comments

It was brought to our attention while completing these revisions that a paper proposing a similar approach to ours was released recently by Koyama et al. (2021). We have since included this article in lines 92-100 of the manuscript,

> "Two similar approaches to ours are that of Chen et al. (2020) and Koyama et al. (2021). The former proposes a two-phase contagion model based on an extension of the Hawkes process. This study considers a continuous-time Hawkes process, assume the rate of external events varies through time, and estimate the change point in their model. The authors also assume there is no external excitation after the change point. The latter of these is, to the authors' knowledge, the most similar approach to ours. These authors consider a discrete-time Hawkes process to describe the current COVID-19 epidemic. This study focusses on estimating a time-varying reproduction number, ignoring the influence of external activity and considering a fixed excitation kernel."

compare our approach to theirs in lines 128-141 of the manuscript,

> "While Koyama et al. (2021) also study more recent data, these authors limit parameter estimation to the reproduction number, and fix the remaining parameters of the Hawkes model. In our study, we estimate the excitation kernel for additional flexibility. Regarding external events, Chen et al. (2020) also assume there is no external excitation in the second phase of their two-phase model. We make no such assumption, and believe considering external excitation throughout the entire course of the pandemic is a valuable consideration. There are still travellers arriving from abroad, and thus exogenous activity is still occurring in later phases at a lower rate. This is particularly relevant as many countries have relatively relaxed quarantine requirements, which means that travellers from abroad are still capable of spreading the virus. Although we study mortality data in this analysis, we are able to make a connection between mortalities and infections. In particular, we show in S1 Appendix that the rate of external events in our model can roughly be interpreted as external infections, times the probability of death given infection. This link is particularly useful in the absence of reliable infection data."

and briefly describe the estimation of the time-varying reproduction number in lines 149-151.

> "For the current COVID-19 pandemic, Koyama et al. (2021) introduce a method for detecting change points in the reproduction number through augmenting their Hawkes model with state-space methods."

References

Bertozzi AL, Franco E, Mohler G, Short MB, Sledge D (2020) The challenges of modeling and forecasting the spread of COVID-19. *Proceedings of the National Academy of Sciences of the United States of America* **117**(29), 16732–16738.

Burda Z (2020) Modelling Excess Mortality in Covid-19-Like Epidemics. *Entropy* **22**, 1236.

Chang SL, Harding N, Zachreson C, Cliff OM, Prokopenko M (2020) Modelling transmission and control of the COVID-19 pandemic in Australia. *Nature Communications* **11**(1), 1–13.

Chen Z, Dassios A, Kuan V, Lim JW, Qu Y, Surya B, Zhao H (2020) A Two-Phase Dynamic Contagion Model for COVID-19. *arXiv* .

Cuevas E (2020) An agent-based model to evaluate the COVID-19 transmission risks in facilities. *Computers in Biology and Medicine* **121**, 103827.

Dennis JM, McGovern AP, Vollmer SJ, Mateen BA (2021) Improving Survival of Critical Care Patients With Coronavirus Disease 2019 in England: A National Cohort Study, March to June 2020*. *Critical Care Medicine* **49**(2), 209–214.

DiMatteo I, Genovese CR, Kass RE (2001) Bayesian curve-fitting with free-knot splines. *Biometrika* **88**(4), 1055–1071.

Funk S, Camacho A, Kucharski AJ, Lowe R, Eggo RM, Edmunds WJ (2019) Assessing the performance of real-time epidemic forecasts: A case study of Ebola in the Western Area region of Sierra Leone, 2014-15. *PLOS Computational Biology* **15**(2), e1006785.

Horwitz LI, Jones SA, Cerfolio RJ, Francois F, Greco J, Rudy B, Petrilli CM (2020) Trends in COVID-19 Risk-Adjusted Mortality Rates. *Journal of Hospital Medicine* **16**(2), 90–92.

Ioannidis JPA, Cripps S, Tanner MA (2020) Forecasting for COVID-19 has failed. *International Journal of Forecasting* .

Kasilingam D, Sathiya Prabhakaran SP, Rajendran DK, Rajagopal V, Santhosh Kumar T, Soundararaj A (2020) Exploring the growth of COVID-19 cases using exponential modelling across 42 countries and predicting signs of early containment using machine learning. *Transboundary and Emerging Diseases* , 1–18.

Kelly JD, Park J, Harrigan RJ, Hoff NA, Lee SD, Wannier R, Selo B, Mossoko M, Njoloko B, Okitolonda-Wemakoy E, Mbala-Kingebeni P, Rutherford GW, Smith TB, Ahuka-Mundeke S, Muyembe-Tamfum JJ, Rimoin AW, Schoenberg FP (2019) Real-time predictions of the 2018–2019 Ebola virus disease outbreak in the Democratic Republic of the Congo using Hawkes point process models. *Epidemics* **28**, 100354.

Killick R, Eckley I (2014) changepoint:an R package for changepoint analysis. *Journal of Statistical Software* **58**(3).

Koyama S, Horie T, Shinomoto S (2021) Estimating the time-varying reproduction number of COVID-19 with a state-space method. *PLOS Computational Biology* **17**(1), e1008679.

Li S, Xie Y, Farajtabar M, Verma A, Song L (2017) Detecting Changes in Dynamic Events Over Networks. *IEEE Transactions on Signal and Information Processing over Networks* **3**(2), 346–359.

Mbuvha R, Marwala T (2020) Bayesian inference of COVID-19 spreading rates in South Africa. *PLOS ONE* **15**(8), e0237126.

Mohler G, Short MB, Schoenberg F, Sledge D (2020) Analyzing the impacts of public policy on COVID-19 transmission in Indiana: The role of model and dataset selection .

Park J, Chaffee AW, Harrigan RJ, Schoenberg FP (2018) A non-parametric hawkes model of the spread of ebola in west africa. *Journal of Applied Statistics, Forthcoming* .

Peixoto PS, Marcondes D, Peixoto C, Oliva SM (2020) Modeling future spread of infections via mobile geolocation data and population dynamics. An application to COVID-19 in Brazil. *PLOS ONE* **15**(7), e0235732.

Petropoulos F, Makridakis S (2020) Forecasting the novel coronavirus COVID-19. *PLOS ONE* **15**(3), e0231236.

Piccolomini EL, Zama F (2020) Monitoring Italian COVID-19 spread by a forced SEIRD model. *PLOS ONE* **15**(8), e0237417.

Rizoiu MA, Mishra S, Kong Q, Carman M, Xie L (2018) SIR-Hawkes: Linking Epidemic Models and Hawkes Processes to Model Diffusions in Finite Populations. In *Proceedings of the 2018 World Wide Web Conference*. International World Wide Web Conferences Steering Committee, Lyon, France, 419–428.

Tadić B, Melnik R (2020) Modeling latent infection transmissions through biosocial stochastic dynamics. *PLOS ONE* **15**(10), e0241163.

The Royal Society (2020) Reproduction number (R) and growth rate (r) of the COVID-19 epidemic in the UK: methods of estimation, data sources, causes of heterogeneity, and use as a guide in policy formulation.

Tovissodé CF, Lokonon BE, Kakaï RG (2020) On the use of growth models to understand epidemic outbreaks with application to COVID-19 data. *PLOS ONE* **15**(10), e0240578.

White G, Porter MD, Mazerolle L (2012) Terrorism Risk, Resilience and Volatility: A Comparison of Terrorism Patterns in Three Southeast Asian Countries. *Journal of Quantitative Criminology* **29**(2), 295–320.

Worden L, Wannier R, Hoff NA, Musene K, Selo B, Mossoko M, Okitolonda-Wemakoy E, Muyembe-Tamfum JJ, Rutherford GW, Lietman TM, Rimoin AW, Porco TC, Kelly JD (2019) Projections of epidemic transmission and estimation of vaccination impact during an ongoing Ebola virus disease outbreak in Northeastern Democratic Republic of Congo, as of Feb. 25, 2019. *PLOS Neglected Tropical Diseases* **13**(8), e0007512.

S1 Appendix: Justification for Hawkes model on deaths

In this section we provide some interpretation of our model on the number of deaths, relating it to the underlying number of infections. This allows us to interpret the parameters in terms of driving parameters for the contamination process.

Hawkes processes are natural objects to model causality through time. Hence, they are well adapted to model the infections, since infections are causing other infections. Assume that at time t (say on the daily basis) N_t^I are newly infected and assume that given the past N_t^I follows a distribution Poisson with rate λ_t^I such that,

$$N_t^I \sim \mathcal{P}(\lambda_t^I), \quad \lambda_t^I = \mu_I + \alpha\beta \sum_{k>0} N_{t-k}^I (1-\beta)^{k-1})$$

where μ_I is the background rate of arrivals with respect to infections.

Consider the simplifying assumption that it takes the same amount of time Δ for a person to die, i.e. people infected at time t will die - given that they will die - at time $t+\Delta$. Hence given that N_t^I people were infected at time t the number of deaths at time $t+\Delta$, $N_{t+\Delta}^D$, is a Binomial (p, N_t^I). When p is small and N_t^I is large this is approximately a Poisson with parameter pN_t^I. This assumption is reasonable since the global death rate is small. Moreover if λ_t^I is large, $N_t^I = \lambda_t^I\Big(1 + O(1/\sqrt{\lambda_t^I})\Big)$. Hence, conditionnally on the past, $N_{t+\Delta}^D$ approximately follows a Poisson distribution with parameter

$$\lambda_{t+\Delta}^D = p\lambda_t^I(1 + 1/\sqrt{\lambda_t^I}) \approx [p\mu_I + \alpha\beta \sum_{k>0} pN_{t-k}^I (1-\beta)^{k-1}](1+o(1)).$$

In a regime where pN_{t-k}^I is large, $N_{t+\Delta-k}^D \approx pN_{t-k}^I$ and to the first order

$$\lambda_{t+\Delta}^D = p\lambda_t^I(1 + 1/\sqrt{\lambda_t^I}) \approx [p\mu_I + \alpha\beta \sum_{k>0} N_{t+\Delta-k}^D (1+o(1))(1-\beta)^{k-1}](1+o(1)).$$

Hence the parameters α and β in the death model have the same meaning as in the infection model. In particular α can be interpreted to the first order as the average number of people contaminated by an infected person and the external arrival rate μ in the death model is roughly the external infection rate μ_I multiplied by the probability of dying given that one is infected.

S2 Appendix: About the average excitation duration

Under the link established in S1 Appendix with the infection model, then what we have is that an infected individual will in the future infect individuals at different times following this model: let us consider case 0, an infected individual,

$$\sum_{t=1}^{\infty} \zeta_t, \quad \zeta_t \sim \mathcal{P}(\alpha\beta(1-\beta)^{t-1})$$

where ζ_t corresponds to the number of individuals infected by case 0. The average number of individuals infected by case 0 is α. One can define the average time of infections from patient 0 as $\bar{T} = \sum_{t=1}^{\infty} t\zeta_t/\alpha$ and its expectation is given by

$$E(\bar{T}) = \sum_{t=1}^{\infty} t\beta(1-\beta)^{t-1} = \frac{1}{\beta}.$$

Hence $1/\beta$ can be interpreted as the expectation of the average time of infections created by patient 0 over the average number of infections. On the other hand, the maximal duration of effective infectiousness is given by $T_0 = \max\{q; \sum_{t\geq q} \zeta_t > 0\}$ so that

$$P(T_0 < q) = P\Big(\sum_{t\geq q} \zeta_t = 0\Big) = e^{-\alpha(1-\beta)^{q-1}}, \quad q \geq 1$$

and

$$E(T_0) = \sum_{q\geq 1} P(T_0 \geq q) = \sum_{q\geq 0} [1 - e^{-\alpha(1-\beta)^q}].$$

Using $u - \frac{u^2}{2} \leq 1 - e^{-u} \leq u, \quad u \geq 0$, we can bound

$$1 - e^{-\alpha} + \frac{\alpha(1-\beta)}{\beta} - \frac{\alpha^2(1-\beta)^2}{4\beta(1-\beta/2)} \leq E(T_0) \leq 1 - e^{-\alpha} + \frac{\alpha(1-\beta)}{\beta}.$$

So that if β is close to 1 (which is the case in the first phase of our analysis),

$$E(T_0) \approx 1 - e^{-\alpha}$$

while if β is close to 0 (and α is not too large),

$$E(T_0) \approx \frac{c(\alpha)}{\beta}, \quad \alpha(1-\alpha/4) \leq c(\alpha) \leq \alpha.$$

S3 Appendix: Convergence and diagnostic plots for initial and subsequent analysis

Subsequent analysis

We now present similar plots for the analysis of the subsequent phases, where $\mu_1, \mu_2 \sim \text{Gamma}(5, 1)$.

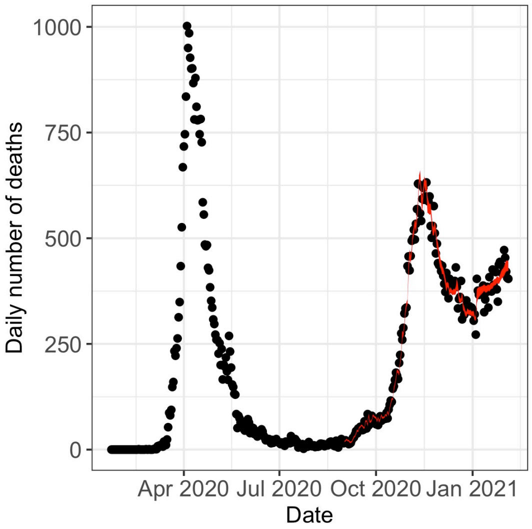

(a)

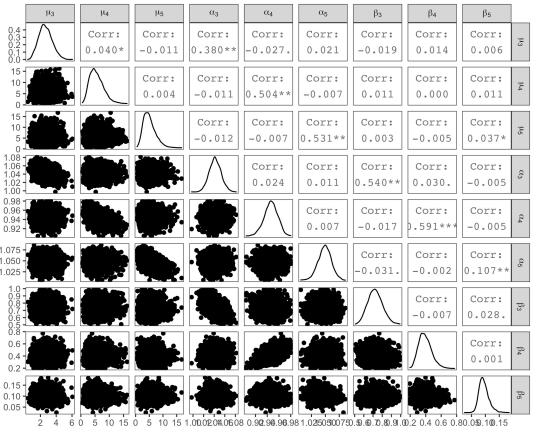

(b)

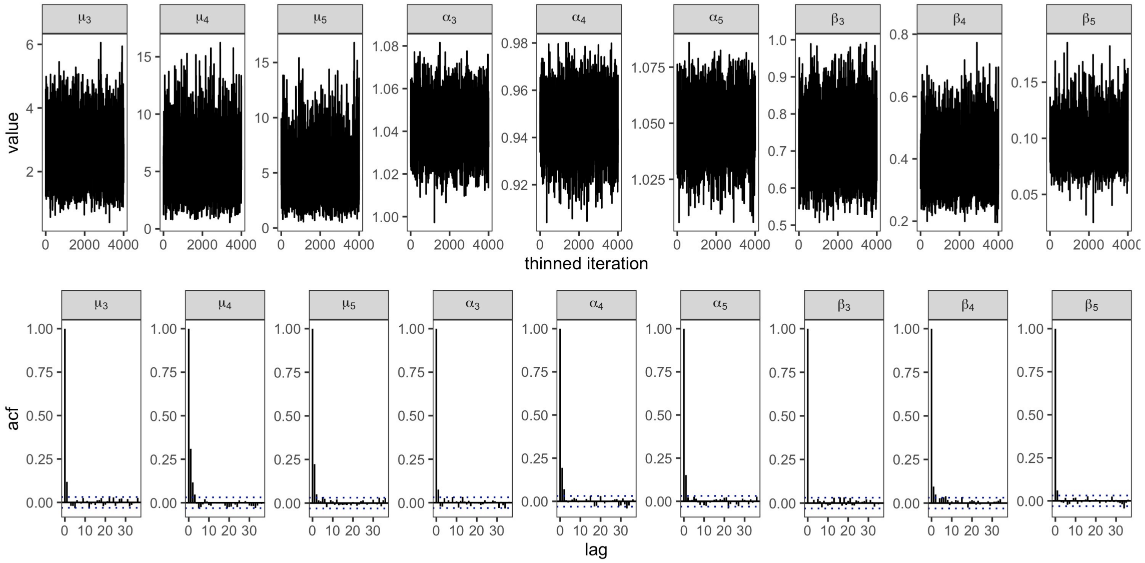

(c)

Figure 7: **Convergence and diagnostic plots for France.** Top left hand panel: compares the observed number of deaths (black dots) with the 95% posterior interval for the estimated expected number of events (solid red ribbon). Top right hand panel: shows pairwise correlation between all parameters in the lower triangle, corresponding correlation values in the upper triangle, and the marginal posterior densities for each parameter on the diagonal. Bottom panel: shows trace plots on the top row and the autocorrelation function on the bottom row for each parameter. All figures were generated after thinning the posterior samples.

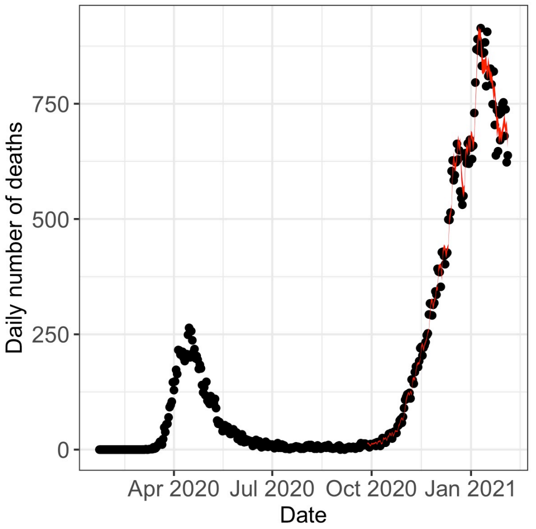

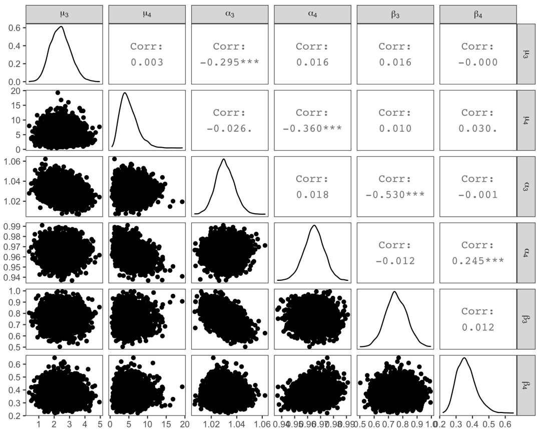

(a) (b)

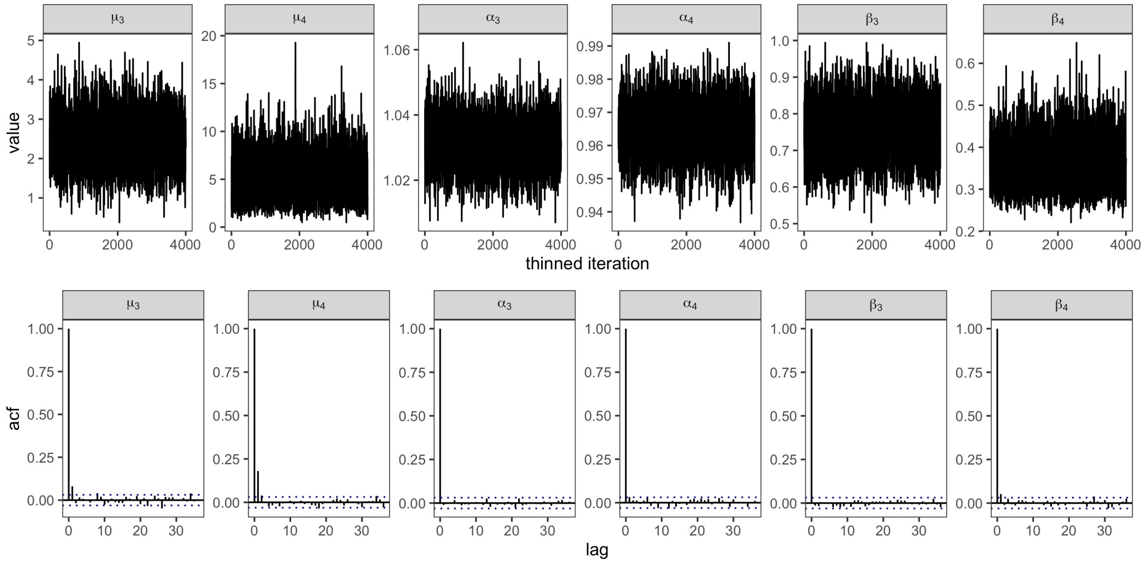

(c)

Figure 8: **Convergence and diagnostic plots for Germany.** Top left hand panel: compares the observed number of deaths (black dots) with the 95% posterior interval for the estimated expected number of events (solid red ribbon). Top right hand panel: shows pairwise correlation between all parameters in the lower triangle, corresponding correlation values in the upper triangle, and the marginal posterior densities for each parameter on the diagonal. Bottom panel: shows trace plots on the top row and the autocorrelation function on the bottom row for each parameter. All figures were generated after thinning the posterior samples.

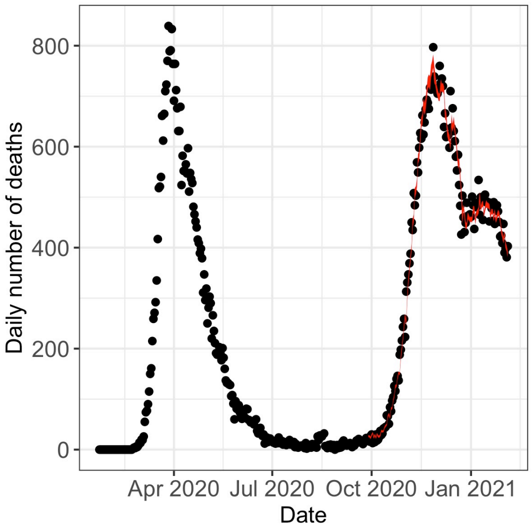

(a)

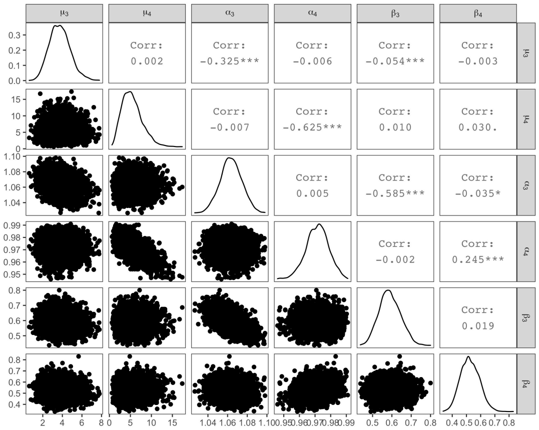

(b)

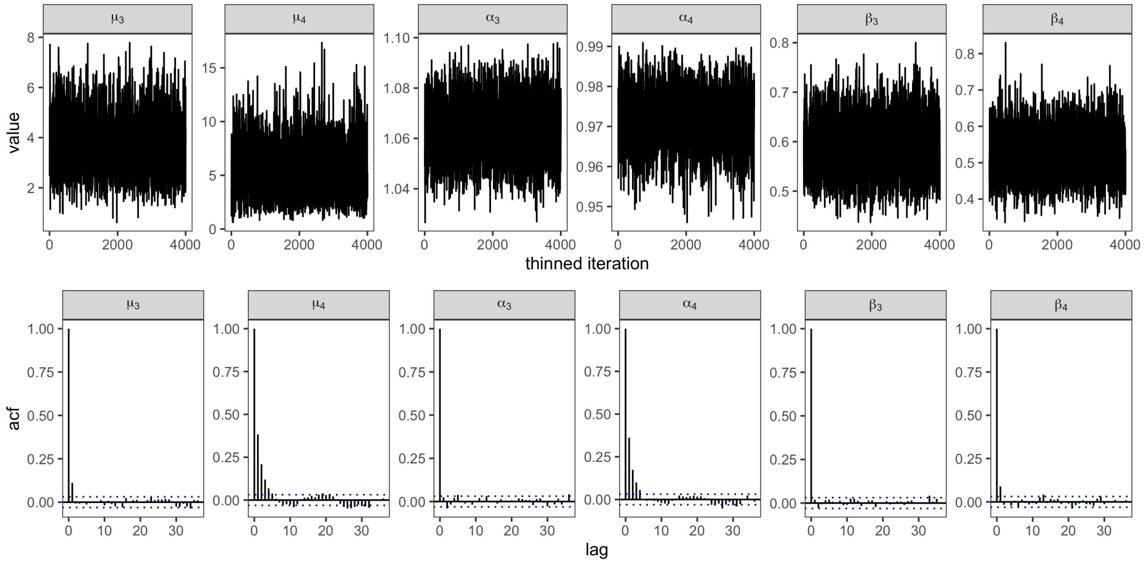

(c)

Figure 9: **Convergence and diagnostic plots for Italy.** Top left hand panel: compares the observed number of deaths (black dots) with the 95% posterior interval for the estimated expected number of events (solid red ribbon). Top right hand panel: shows pairwise correlation between all parameters in the lower triangle, corresponding correlation values in the upper triangle, and the marginal posterior densities for each parameter on the diagonal. Bottom panel: shows trace plots on the top row and the autocorrelation function on the bottom row for each parameter. All figures were generated after thinning the posterior samples.

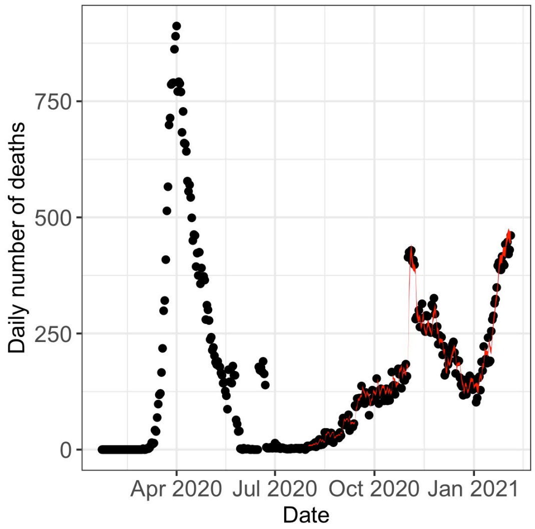

(a)

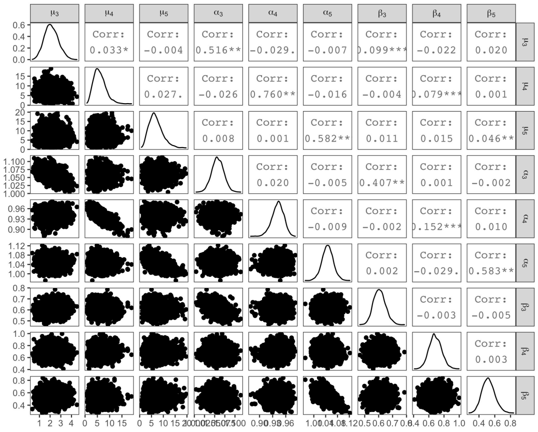

(b)

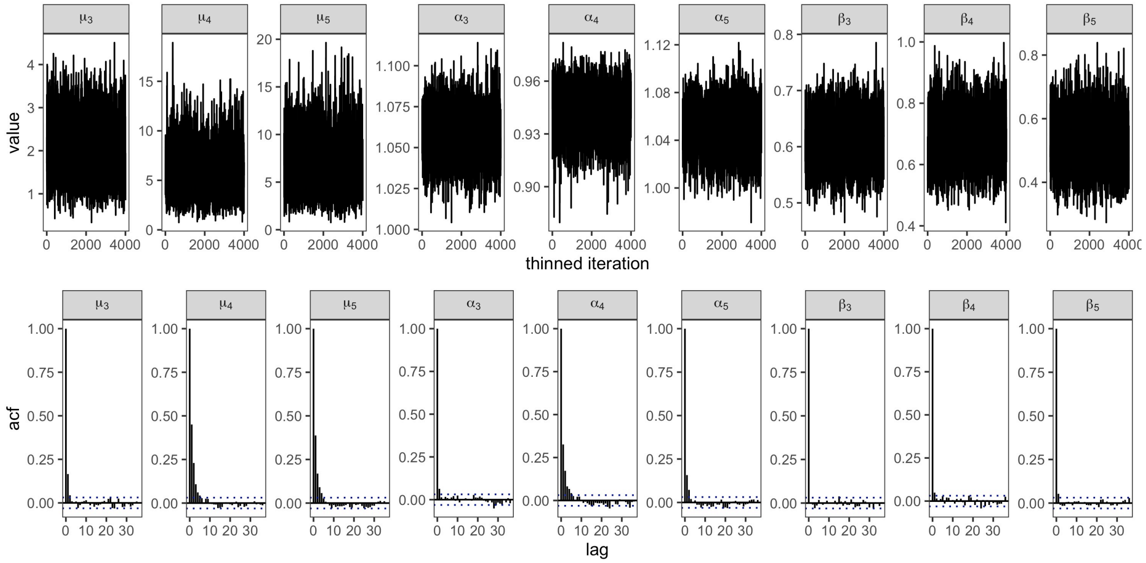

(c)

Figure 10: **Convergence and diagnostic plots for Spain.** Top left hand panel: compares the observed number of deaths (black dots) with the 95% posterior interval for the estimated expected number of events (solid red ribbon). Top right hand panel: shows pairwise correlation between all parameters in the lower triangle, corresponding correlation values in the upper triangle, and the marginal posterior densities for each parameter on the diagonal. Bottom panel: shows trace plots on the top row and the autocorrelation function on the bottom row for each parameter. All figures were generated after thinning the posterior samples.

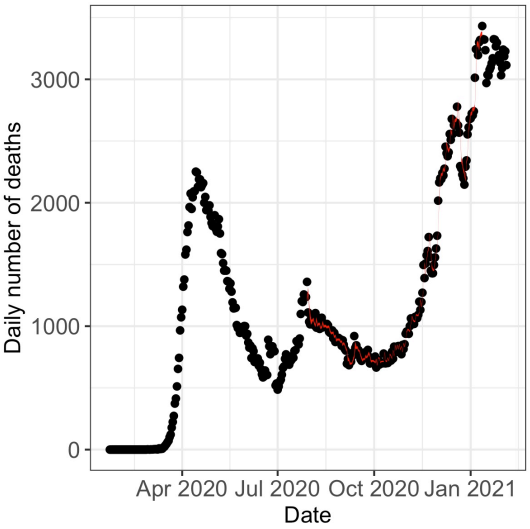

(a)

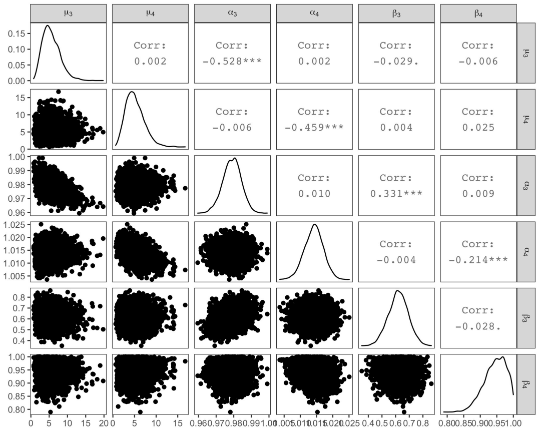

(b)

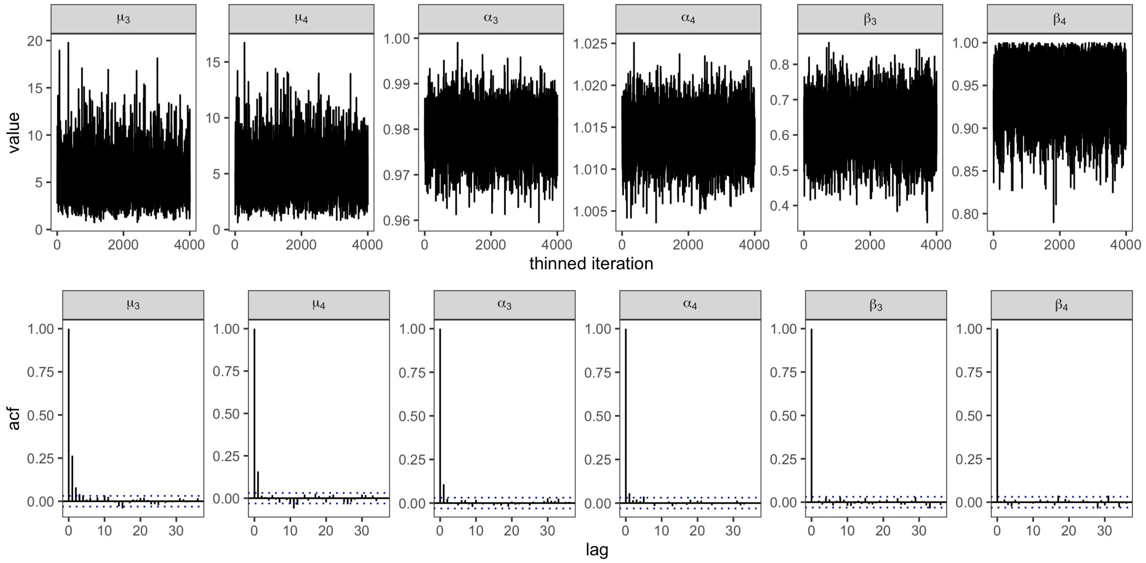

(c)

Figure 11: **Convergence and diagnostic plots for the U.S.** Top left hand panel: compares the observed number of deaths (black dots) with the 95% posterior interval for the estimated expected number of events (solid red ribbon). Top right hand panel: shows pairwise correlation between all parameters in the lower triangle, corresponding correlation values in the upper triangle, and the marginal posterior densities for each parameter on the diagonal. Bottom panel: shows trace plots on the top row and the autocorrelation function on the bottom row for each parameter. All figures were generated after thinning the posterior samples.

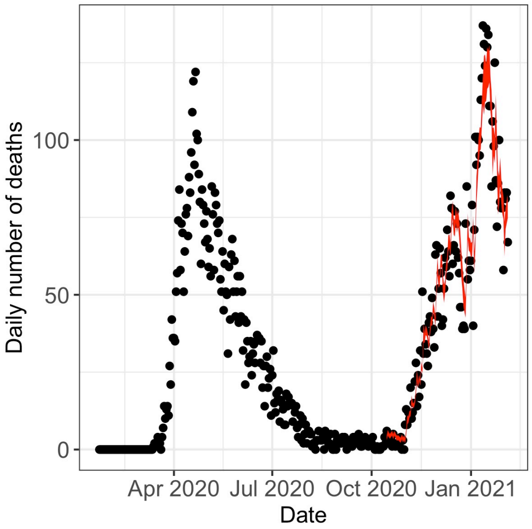

(a)

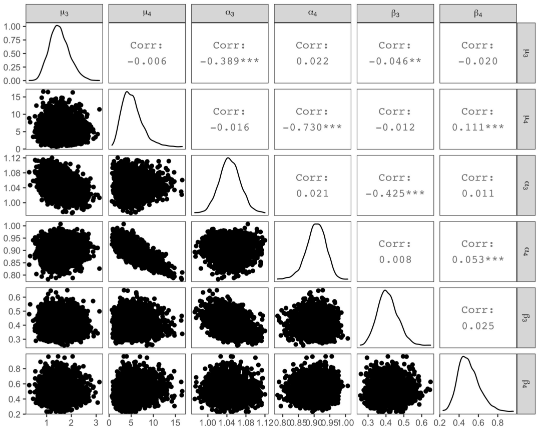

(b)

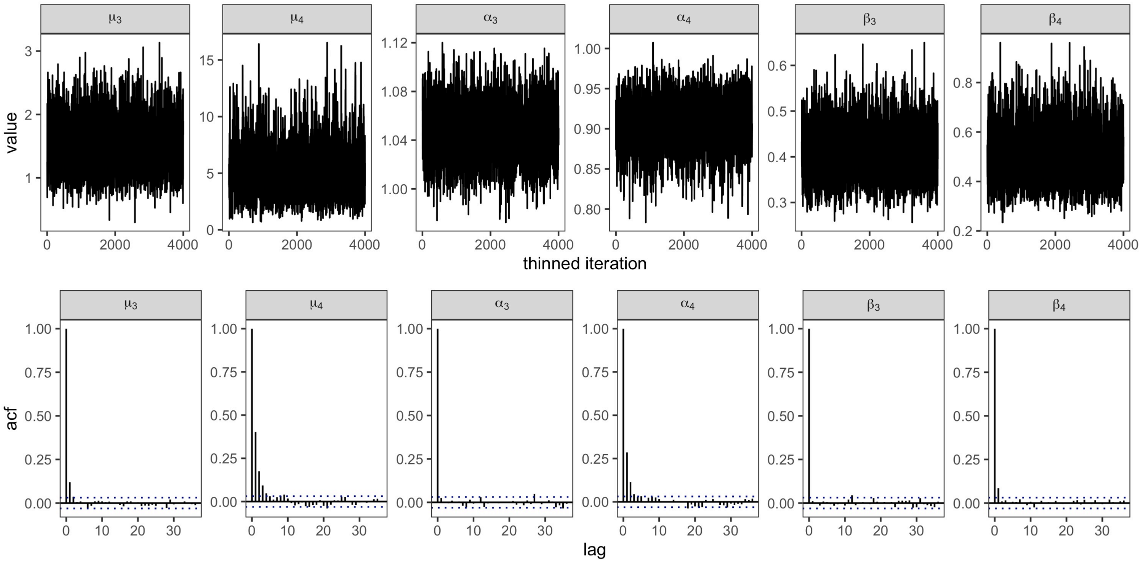

(c)

Figure 12: **Convergence and diagnostic plots for Sweden.** Top left hand panel: compares the observed number of deaths (black dots) with the 95% posterior interval for the estimated expected number of events (solid red ribbon). Top right hand panel: shows pairwise correlation between all parameters in the lower triangle, corresponding correlation values in the upper triangle, and the marginal posterior densities for each parameter on the diagonal. Bottom panel: shows trace plots on the top row and the autocorrelation function on the bottom row for each parameter. All figures were generated after thinning the posterior samples.

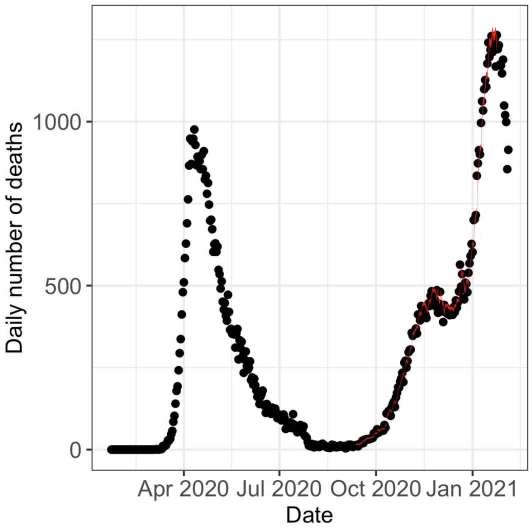

(a)

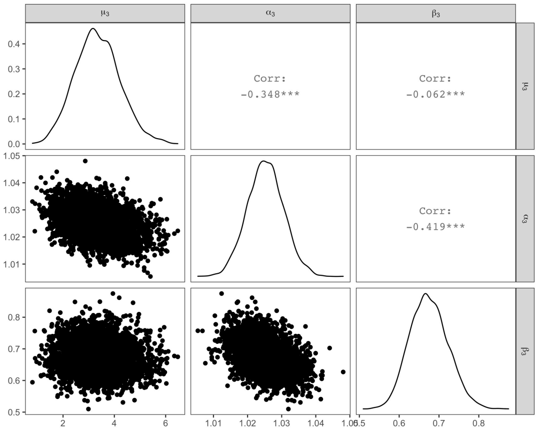

(b)

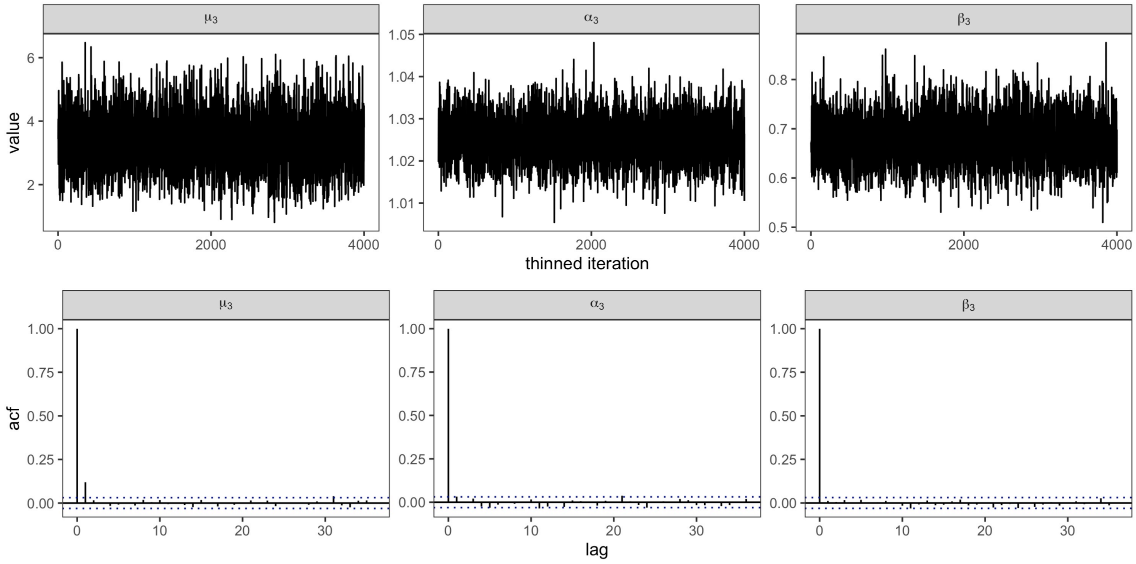

(c)

Figure 13: **Convergence and diagnostic plots for the U.K.** Top left hand panel: compares the observed number of deaths (black dots) with the 95% posterior interval for the estimated expected number of events (solid red ribbon). Top right hand panel: shows pairwise correlation between all parameters in the lower triangle, corresponding correlation values in the upper triangle, and the marginal posterior densities for each parameter on the diagonal. Bottom panel: shows trace plots on the top row and the autocorrelation function on the bottom row for each parameter. All figures were generated after thinning the posterior samples.

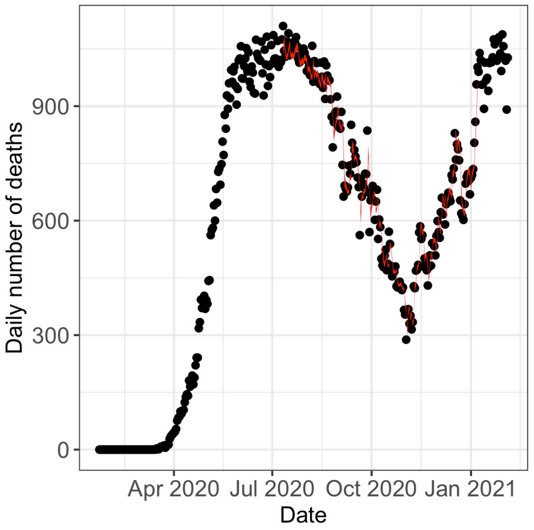

(a)

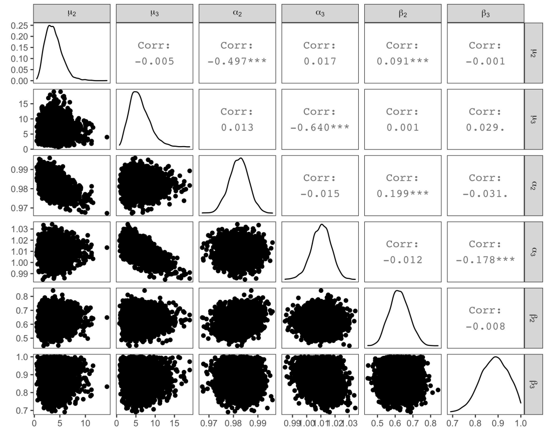

(b)

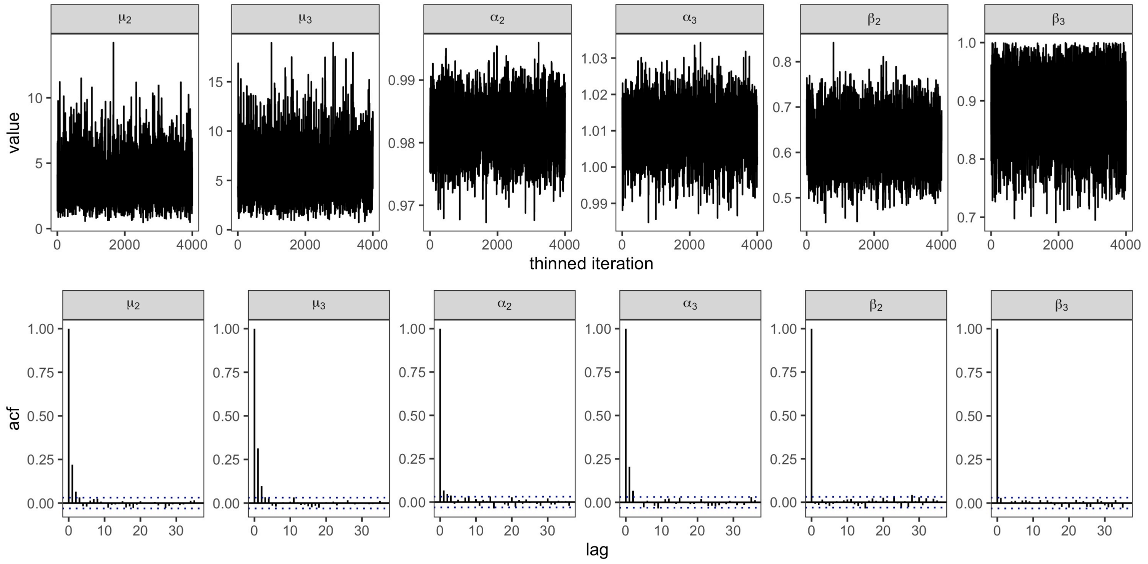

(c)

Figure 14: **Convergence and diagnostic plots for Brazil.** Top left hand panel: compares the observed number of deaths (black dots) with the 95% posterior interval for the estimated expected number of events (solid red ribbon). Top right hand panel: shows pairwise correlation between all parameters in the lower triangle, corresponding correlation values in the upper triangle, and the marginal posterior densities for each parameter on the diagonal. Bottom panel: shows trace plots on the top row and the autocorrelation function on the bottom row for each parameter. All figures were generated after thinning the posterior samples.

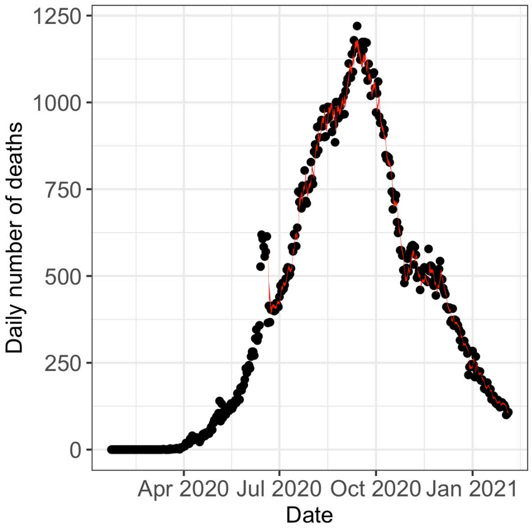

(a)

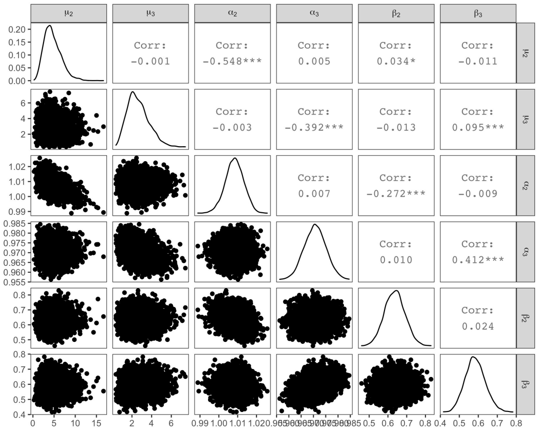

(b)

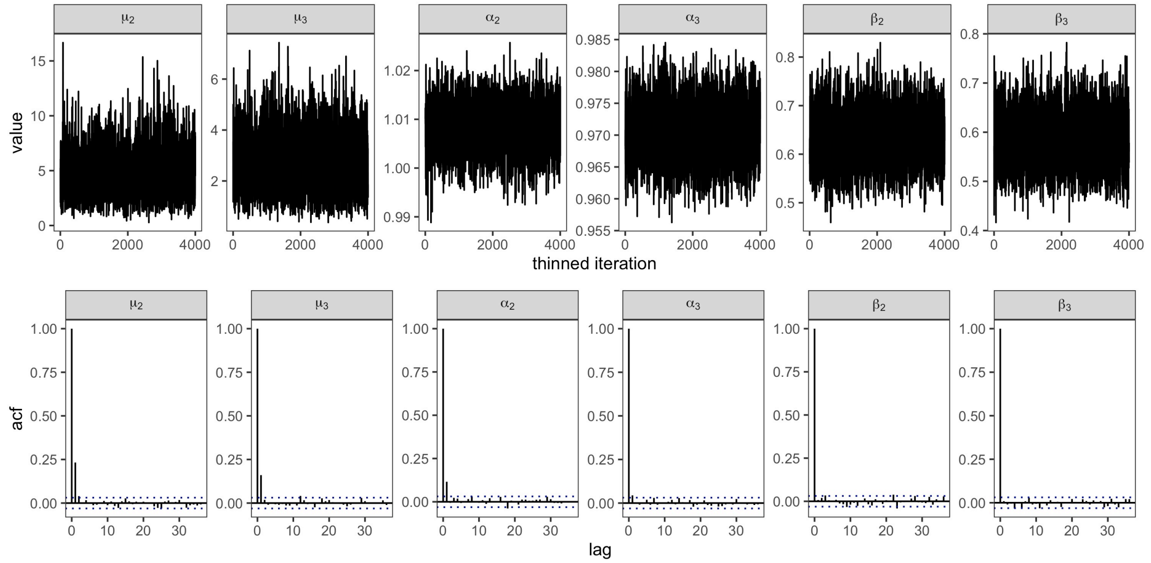

(c)

Figure 15: **Convergence and diagnostic plots for India.** Top left hand panel: compares the observed number of deaths (black dots) with the 95% posterior interval for the estimated expected number of events (solid red ribbon). Top right hand panel: shows pairwise correlation between all parameters in the lower triangle, corresponding correlation values in the upper triangle, and the marginal posterior densities for each parameter on the diagonal. Bottom panel: shows trace plots on the top row and the autocorrelation function on the bottom row for each parameter. All figures were generated after thinning the posterior samples.

S7 Appendix: Change point locations

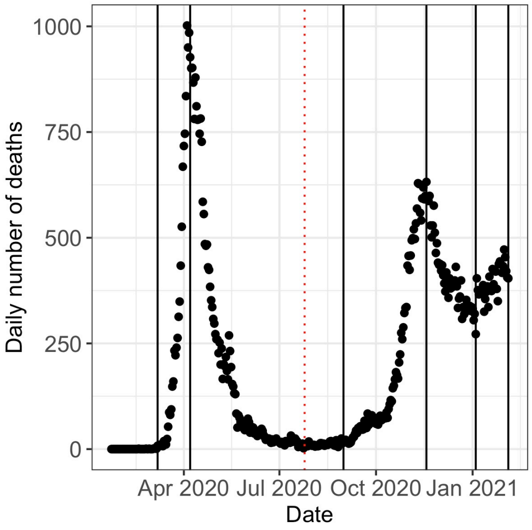

(a) France.

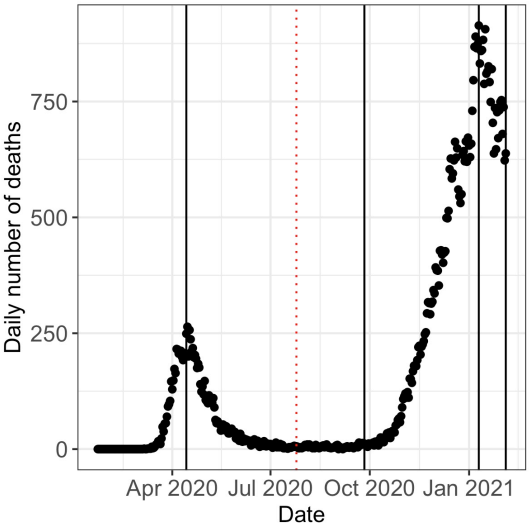

(b) Germany

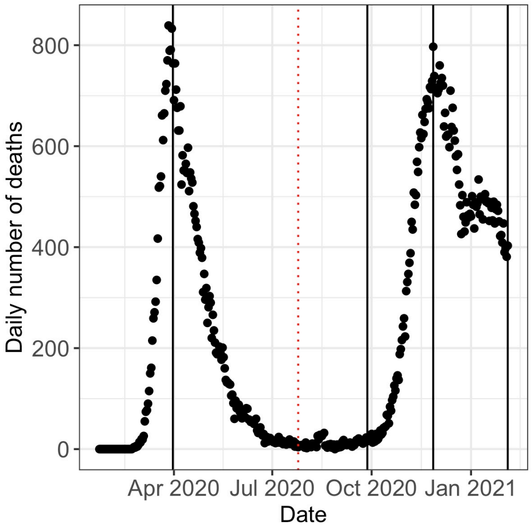

(c) Italy

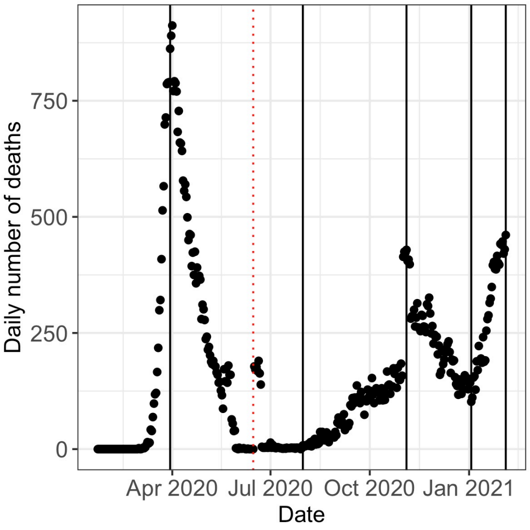

(d) Spain

Figure 16: **Change point locations.** Black vertical lines: change point locations. Black dots: observed data. Red vertical line (dotted): endpoint of initial analysis.

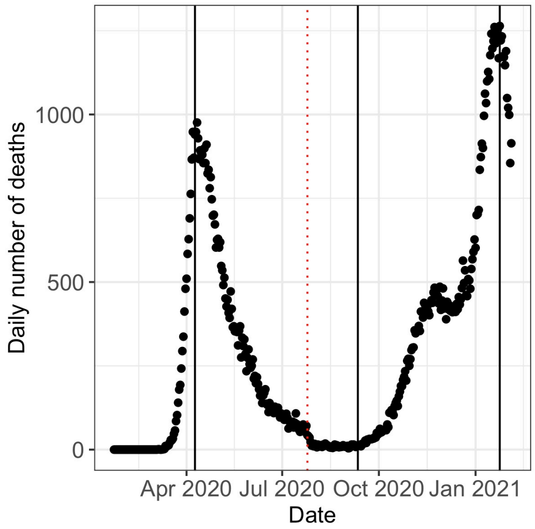

(e) U.K.

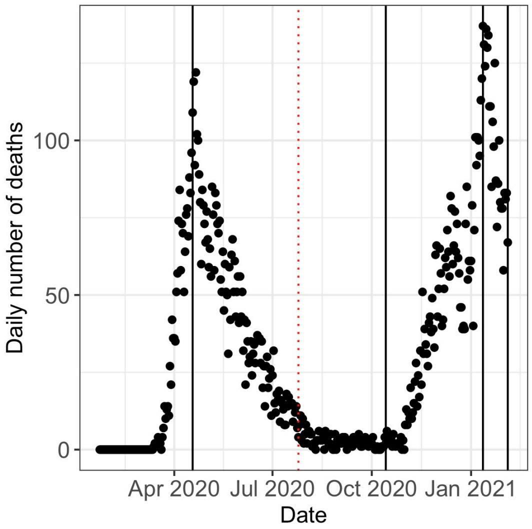

(f) Sweden

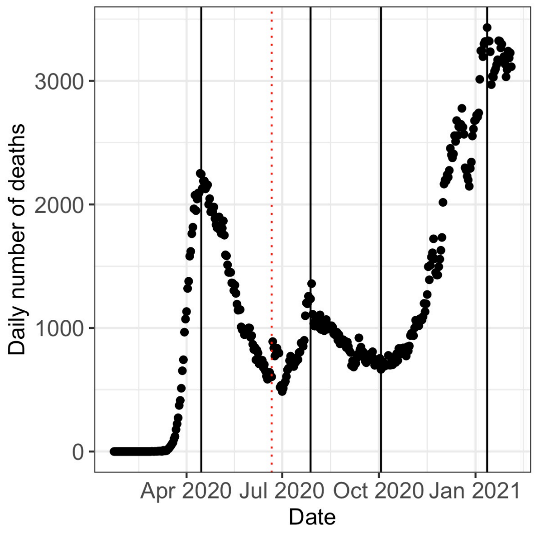

(g) U.S.

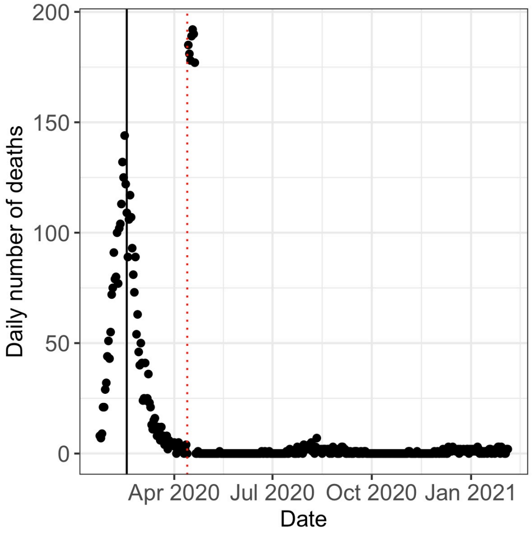

(h) China

Figure 16: (cont.)

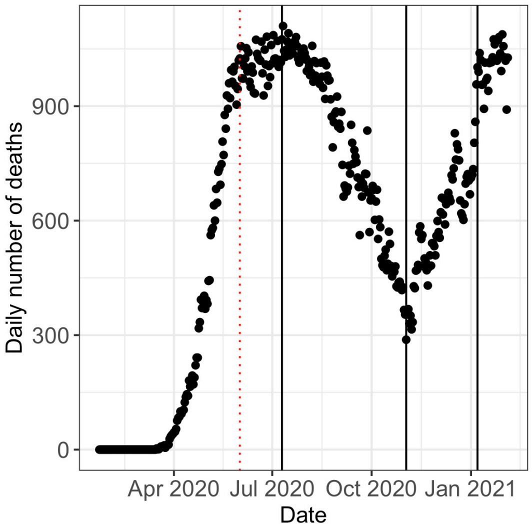

(i) Brazil

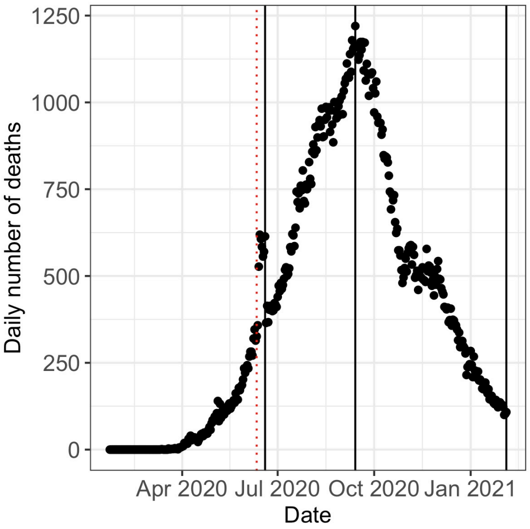

(j) India

Figure 16: (cont.)

Country	Phase	Start Date	End Date
Italy	1	26th Feb 2020	31st Mar 2020
	2	1st Apr 2020	25th July 2020
	3	28th Sep 2020	27th Nov 2020
	4	28th Nov 2020	4th Feb 20201
France	1	7th Mar 2020	7th Apr 2020
	2	8th Apr 2020	25th July 2020
	3	1st Sep 2020	18th Nov 2020
	4	19th Nov 2020	4th Jan 2021
	5	5th Jan 2021	4th Feb 2021
Spain	1	7th Mar 2020	30th Mar 2020
	2	31st Mar 2020	15th June
	3	1st Aug 2020	4th Nov 2020
	4	5th Nov 2020	3rd Jan 2021
	5	4th Jan 2021	4th Feb 2021
Germany	1	13th Mar 2020	14th Apr 2020
	2	15th Apr 2020	25th July 2020
	3	27th Sep 2020	10th Jan 2021
	4	11th Jan 2021	4th Feb 2021
Sweden	1	17th Mar 2020	18th Apr 2020
	2	19th Apr 2020	25th July 2020
	3	15th Oct 2020	12th Jan 2021
	4	13th Jan 2021	4th Feb 2021
UK	1	12th Mar 2020	9th Apr 2020
	2	10th Apr 2020	25th July 2020
	3	12th Sep 2020	24th Jan 2021
US	1	5th Mar 2020	15th Apr 2020
	2	16th Apr 2020	21st June 2020
	3	29th July 2020	3rd Oct 2020
	4	4th Oct 2020	12th Jan 2020
China	1	24th Jan 2020	17th Feb 2020
	2	18th Feb 2020	13th Apr 2020
Brazil	1	3rd Mar 2020	1st June
	2	11th July 2020	2nd Nov 2020
	3	3rd Nov	7th Jan 2021
India	1	23rd Mar 2020	11th June 2020
	2	20th June 2020	13th Sep 2020
	3	14th Sep 2020	4th Feb 2021

Table 2: Dates of phases

S8 Appendix: Plot of residuals

To calculate the residuals, we first determine the difference between the estimated conditional intensity function obtained for each of our posterior samples, and the observed number of deaths. We then take the median residual at each time point to produce the histograms in Fig 17. The residuals are generally centred around zero and higher values are less frequent. Cases where this is not necessarily the case generally arise from phases that have a shorter time series used to estimate the parameters, or where the observed data is not smooth. Additionally, cases where there is a single residual that is significantly larger than the rest are usually either at the peaks or where anomalies are present in the data.

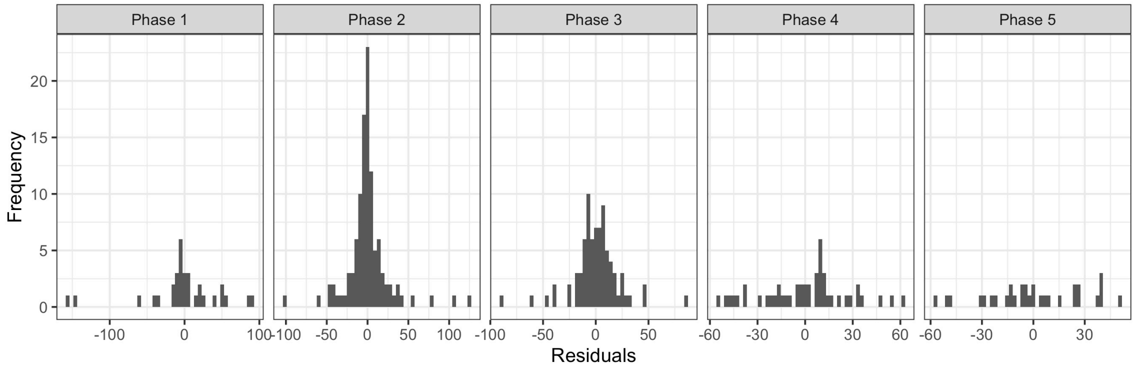

(a) France

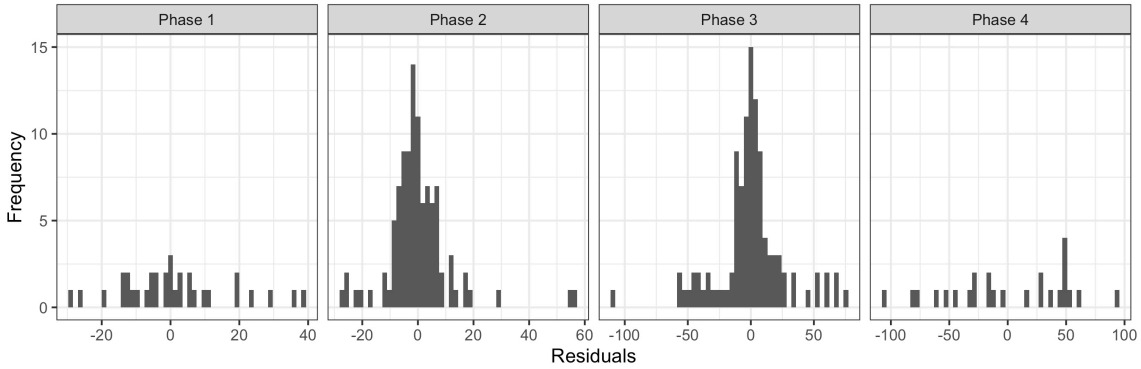

(b) Germany

Figure 17: **Plot of residuals** For each country and phase, we calculate the estimated expected intensity of the process (i.e. $\lambda(t)$) using the samples of the parameter estimates obtained through the estimation procedure. The histograms then represent the median residual value (median of the difference between the observed number of events and the estimated expected intensity).

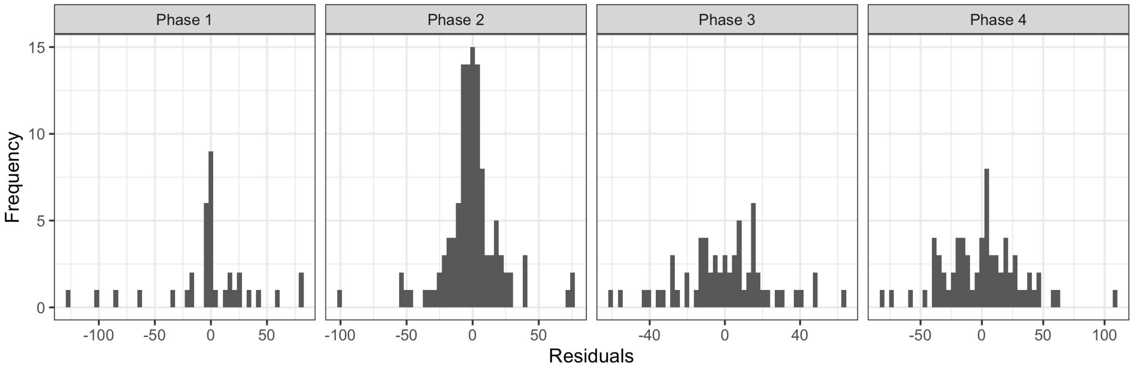

(c) Italy

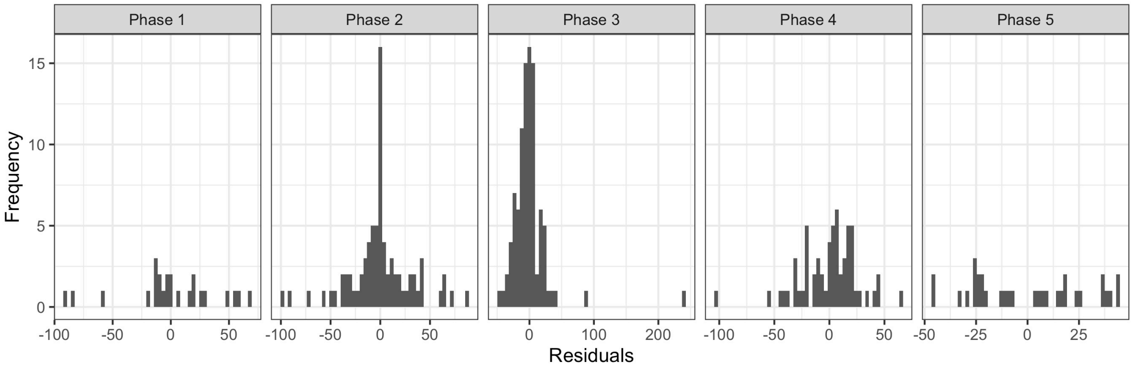

(d) Spain

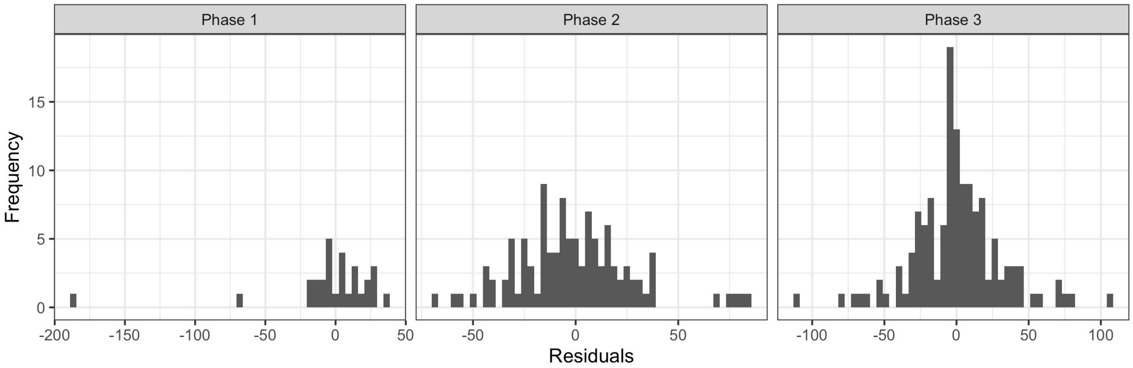

(e) U.K.

Figure 17: (cont.)

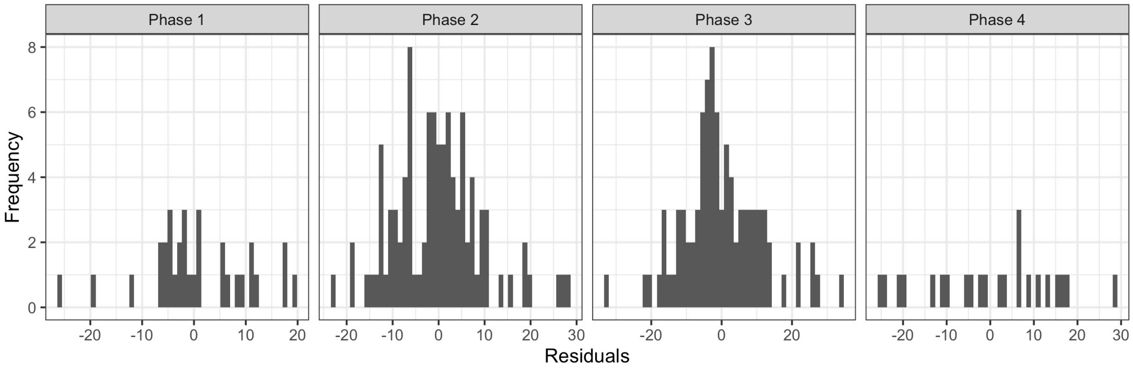

(f) Sweden

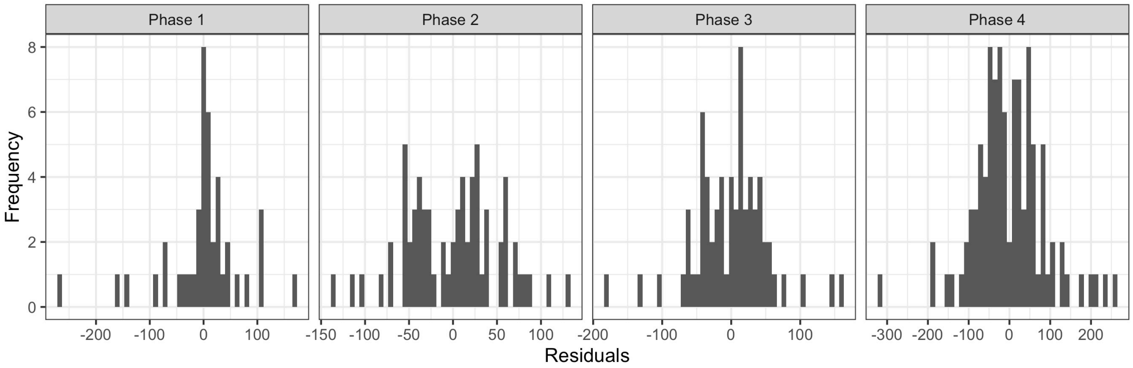

(g) U.S.

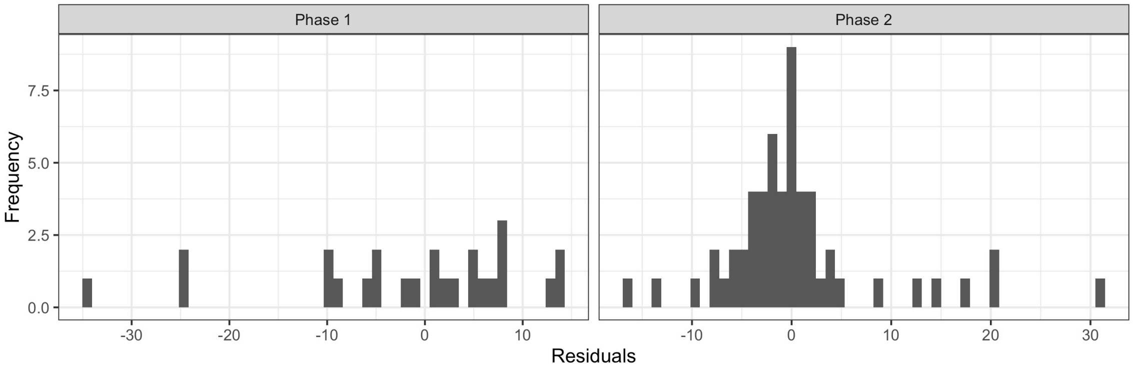

(h) China

Figure 17: (cont.)

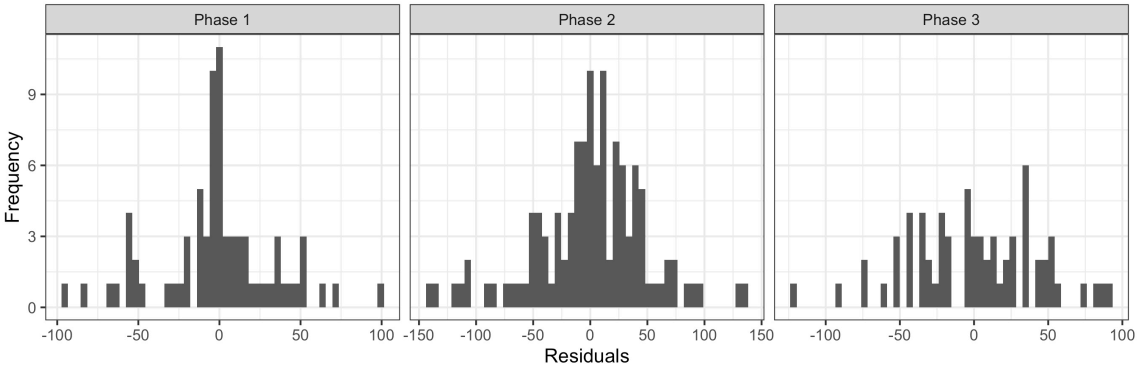

(i) Brazil

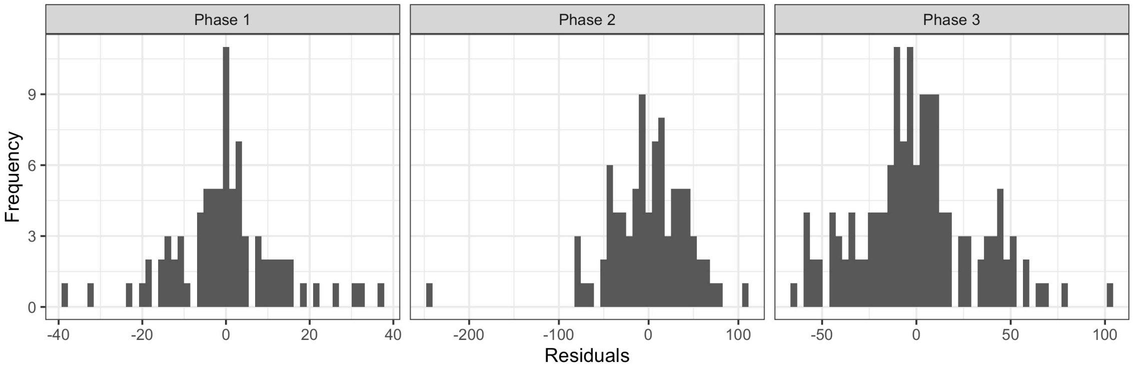

(j) India

Figure 17: (cont.)

S2 Table: Parameter estimates for original and subsequent analysis.

Country	Phase	μ	α	β
Italy	1	4.39 (3.18,5.71)	1.07 (1.05,1.09)	0.88 (0.8,0.95)
	2	1.17 (0.69,1.8)	0.94 (0.93,0.95)	0.55 (0.48,0.63)
	3	3.7 (2.41,5.1)	1.06 (1.05,1.08)	0.59 (0.52,0.66)
	4	5.14 (2.59,8.68)	0.97 (0.96,0.98)	0.52 (0.44,0.61)
France	1	4.57 (3.38,5.91)	1.1 (1.08,1.11)	0.97 (0.92,0.99)
	2	1.57 (0.97,2.28)	0.92 (0.91,0.93)	0.64 (0.58,0.7)
	3	2.58 (1.59,3.75)	1.04 (1.03,1.06)	0.72 (0.62,0.83)
	4	5.24 (2.73,8.82)	0.95 (0.93,0.96)	0.38 (0.29,0.5)
	5	4.71 (2.53,8.17)	1.05 (1.03,1.06)	0.09 (0.07,0.12)
Spain	1	5.78 (4.06,7.6)	1.11 (1.09,1.13)	0.96 (0.9,0.99)
	2	0.49 (0.28,0.76)	0.96 (0.95,0.97)	0.91 (0.85,0.95)
	3	2.08 (1.31,2.98)	1.06 (1.04,1.08)	0.6 (0.55,0.66)
	4	5.64 (3.04,9.1)	0.94 (0.92,0.96)	0.68 (0.59,0.79)
	5	6.46 (3.46,10.68)	1.05 (1.02,1.07)	0.52 (0.42,0.63)
Germany	1	4.17 (2.89,5.54)	1.06 (1.03,1.09)	0.65 (0.57,0.75)
	2	0.95 (0.59,1.39)	0.91 (0.89,0.93)	0.51 (0.45,0.59)
	3	2.39 (1.61,3.25)	1.03 (1.02,1.04)	0.75 (0.66,0.85)
	4	4.63 (2.4,8.02)	0.97 (0.95,0.97)	0.36 (0.29,0.44)
Sweden	1	4.05 (2.88,5.44)	1.07 (1.01,1.13)	0.42 (0.32,0.54)
	2	1.79 (1.05,2.68)	0.92 (0.89,0.95)	0.5 (0.39,0.62)
	3	1.49 (1.02,2.05)	1.05 (1.02,1.07)	0.41 (0.34,0.48)
	4	4.92 (2.61,8.16)	0.91 (0.87,0.94)	0.49 (0.37,0.65)
UK	1	4.51 (3.08,6)	1.14 (1.11,1.17)	0.79 (0.68,0.91)
	2	2.42 (1.32,3.75)	0.95 (0.95,0.96)	0.56 (0.5,0.62)
	3	3.3 (2.25,4.48)	1.03 (1.02,1.03)	0.67 (0.61,0.74)
US	1	4.08 (3.13,5.15)	1.07 (1.06,1.07)	0.99 (0.98,1)
	2	4.1 (2.16,7.12)	0.97 (0.97,0.98)	0.77 (0.66,0.89)
	3	5.28 (2.81,8.8)	0.98 (0.97,0.99)	0.62 (0.53,0.71)
	4	4.85 (2.62,7.93)	1.01 (1.01,1.02)	0.95 (0.9,0.99)
China	1	8.92 (6.29,11.73)	1.07 (1.01,1.15)	0.4 (0.28,0.56)
	2	0.82 (0.48,1.22)	0.8 (0.76,0.84)	0.43 (0.35,0.54)
Brazil	1	4.18 (2.98,5.52)	1.03 (1.02,1.04)	0.83 (0.73,0.93)
	2	3.62 (1.87,5.97)	0.98 (0.98,0.99)	0.62 (0.55,0.69)
	3	5.63 (2.96,9.35)	1.01 (1,1.02)	0.89 (0.8,0.96)
India	1	2.81 (2.02,3.72)	1.1 (1.07,1.13)	0.33 (0.26,0.41)
	2	4.34 (2.31,7.35)	1.01 (1,1.01)	0.64 (0.57,0.71)
	3	2.49 (1.33,4.11)	0.97 (0.97,0.98)	0.58 (0.52,0.65)

Table 3: Comparison of median and 80% intervals of parameters for all phases, using the Gamma$(5, 1)$ prior for μ.
